# The Amyloid Cascade Hypothesis 2.0 for Alzheimer’s Disease and Aging-Associated Cognitive Decline: From Molecular Basis to Effective Therapy

**DOI:** 10.3390/ijms241512246

**Published:** 2023-07-31

**Authors:** Vladimir Volloch, Sophia Rits-Volloch

**Affiliations:** 1 Department of Developmental Biology, Harvard School of Dental Medicine, Boston, MA 02115, USA; 2 Division of Molecular Medicine, Children’s Hospital, Boston, MA 02115, USA; 3 Department of Biological Chemistry and Molecular Pharmacology, Harvard Medical School, Boston, MA 02115, USA

**Keywords:** the amyloid cascade hypothesis 2.0 (ACH2.0), intraneuronal Aβ (*i*Aβ), Aβ protein precursor (AβPP)-independent generation of *i*Aβ, aging-related cognitive dysfunction (AACD), *i*Aβ depletion therapy for AD and AACD, BACE1 and BACE2 activators as AD and AACD drugs

## Abstract

With the long-standing amyloid cascade hypothesis (ACH) largely discredited, there is an acute need for a new all-encompassing interpretation of Alzheimer’s disease (AD). Whereas such a recently proposed theory of AD is designated ACH2.0, its commonality with the ACH is limited to the recognition of the centrality of amyloid-β (Aβ) in the disease, necessitated by the observation that *all* AD-causing mutations affect, in one way or another, Aβ. Yet, even this narrow commonality is superficial since AD-causing Aβ of the ACH differs distinctly from that specified in the ACH2.0: Whereas in the former, the disease is caused by secreted extracellular Aβ, in the latter, it is triggered by Aβ-protein-precursor (AβPP)-derived *intraneuronal* Aβ (*i*Aβ) and driven by *i*Aβ generated *independently* of AβPP. The ACH2.0 envisions AD as a two-stage disorder. The first, asymptomatic stage is a decades-long accumulation of AβPP-derived *i*Aβ, which occurs via internalization of secreted Aβ and through intracellular retention of a fraction of Aβ produced by AβPP proteolysis. When AβPP-derived *i*Aβ reaches critical levels, it activates a self-perpetuating AβPP-independent production of *i*Aβ that drives the second, devastating AD stage, a cascade that includes tau pathology and culminates in neuronal loss. The present study analyzes the dynamics of *i*Aβ accumulation in health and disease and concludes that it is the prime factor driving both AD and aging-associated cognitive decline (AACD). It discusses mechanisms potentially involved in AβPP-independent generation of *i*Aβ, provides mechanistic interpretations for all principal aspects of AD and AACD including the protective effect of the Icelandic AβPP mutation, the early onset of FAD and the sequential manifestation of AD pathology in defined regions of the affected brain, and explains why current mouse AD models are neither adequate nor suitable. It posits that while drugs affecting the accumulation of AβPP-derived *i*Aβ can be effective only protectively for AD, the targeted degradation of *i*Aβ is the best therapeutic strategy for both prevention and effective treatment of AD and AACD. It also proposes potential *i*Aβ-degrading drugs.

## 1. Introduction

The designation of the recently proposed interpretation of Alzheimer’s disease (AD) and aging-associated cognitive decline (AACD), amyloid cascade hypothesis 2.0 (ACH2.0), refers to its predecessor, the ACH. However, despite the similarity of names, the commonality between these two theories of AD is restricted to the recognition of the centrality of amyloid-beta (Aβ) in the disease. But even this narrow commonality is rather superficial: whereas in the ACH, AD is caused by extracellular Aβ produced and secreted in the Aβ protein precursor (AβPP)-proteolytic pathway, in the ACH2.0, the disease is triggered by AβPP-derived intraneuronal Aβ (*i*Aβ) accumulated to sufficient levels and is driven by *i*Aβ generated independently of AβPP. The rationale for the 

ACH2.0, as well as its various aspects, has been discussed in detail elsewhere [1,2,3]. The present discourse takes the preceding studies as the established point of departure. One of its main objectives is to analyze the kinetic parameters of the ACH.2.0. As detailed below, this analysis concludes that the dynamics of *i*Aβ accumulation plays the key role in the commencement and progression of both AD and AACD. This notion is best illustrated by the observations that *all* known AD-causing mutations elevate the rate of *i*Aβ accumulation, whereas the mutation that protects from both AD and AACD suppresses it [4,5]. The dynamics of *i*Aβ accumulation points to feasible options of interference with AD and AACD and its understanding is essential for the development of the efficient therapeutic strategies, which are discussed in detail below.

To better orient the reader, the following few sections (Section 2, Section 3, Section 4, Section 5 and Section 6), preceding the analysis of the dynamics of *i*Aβ accumulation, briefly summarize four main principles of the ACH 2.0 (for detailed description and analysis see [1,2]). They are: (1)—AD and AACD are caused and driven by intra- rather than extracellular Aβ; (2)—AD (but not AACD) is a two-stage disease; (3)—the symptomatic second stage of AD is driven by *i*Aβ produced in the AβPP-independent pathway; (4)—*i*Aβ generated independently of AβPP is retained intraneuronally, perpetuates its own production and renders AβPP-derived *i*Aβ irrelevant for the progression of AD due to its marginal (in comparison with *i*Aβ produced independently of AβPP) contribution into the cellular *i*Aβ pool.

## 2. Amyloid Cascade Hypothesis: A Proposition in Distress

In 1992, Hardy and Higgins proposed the long-standing amyloid cascade hypothesis, ACH, for AD [6]. They formulated it as follows: “Our hypothesis is that deposition of amyloid β protein, the main component of the plaques, is the causative agent of Alzheimer’s pathology and that the neurofibrillary tangles, cell loss, vascular damage, and dementia follow as the direct result of this deposition” [6]. The principal basis for this assertion was, in addition to the prominent occurrence of Aβ plaques, the then recent discovery of a mutation within AβPP [7] that affected the generation of Aβ in the AβPP proteolytic pathway and segregated with, and apparently caused, familial AD (FAD). At the time, the ACH appeared to be consistent with all preceding observations; it was widely accepted and formed the long-lasting foundation for basic and clinical research. Consequently, extracellular Aβ and its production in the AβPP proteolytic/secretory pathway became the major therapeutic targets of the disease. The research and development efforts resulted in numerous candidate AD drugs. Many of those drugs exhibited dramatic successes in preclinical studies and animal trials. As an example, suppression of BACE1 activity, and thus of Aβ production, by different means resulted in a significant improvement of neurophysiological functions and, moreover, in the dramatic reversal of AD symptoms in animal models where Aβ was overproduced exogenously [8,9,10]. 

However, with few exceptions (lecanemab and donanemab, which are marginally effective in very early symptomatic AD but apparently act preventively rather than curatively [3]), all AD candidate drugs showed no efficacy whatsoever in symptomatic AD clinical trials, which were consequently declared failures. On the other hand, the close analysis of the results of “failed” clinical trials indicates that many candidate AD drugs succeeded remarkably in their mechanistic mission. For example, verubecestat, a BACE1 inhibitor, effectively suppressed production of Aβ in the AβPP proteolytic pathway and substantially cleared extracellular Aβ in AD patients, as reflected in the up to 80% reduction, in a dose-dependent manner, of the levels of Aβ in CSF [11,12]. The observations that the drug effectively accomplished exactly what it was designed for without eliciting any clinical improvements in patients implied that the underlying theory of AD, the ACH, is incorrect and mandated different interpretation of the disease. Failures of clinical trials of ACH-based AD drugs were exacerbated by observations showing that there is no good correlation between extracellular Aβ deposit levels and the occurrence of the disease. Indeed, a significant subset of the general human population develops, with aging, excessive levels of extracellular Aβ, comparable with or greater than those typically seen in AD, yet shows no cognitive dysfunction or AD pathology [13,14,15,16,17,18,19]. In a diametrically opposite example, some individuals with cognitive AD symptoms and the occurrence of AD pathology show no excessive levels of extracellular Aβ [20]. Taken cumulatively, the above considerations apparently rule out the causative role of extracellular Aβ in AD.

## 3. The Centrality of Amyloid-β Is Requisite for Any Theory of AD: ACH2.0

Yet another set of observations powerfully attests to the centrality and the causative role of Aβ in AD. In the over three decades since the discovery of the first Aβ-affecting, AD-causing mutation [7], many more mutations that cause AD have been detected and characterized. *All* of them, with no exception, affect either the structure of Aβ or various aspects of its production in the AβPP proteolytic pathway. Moreover, the mutation known to confer on its carriers the protection from both AD and AACD replaces a single amino acid within Aβ [4,5]. In other words, introduce a single Aβ modification and cause AD; substitute a single Aβ residue and protect from both AD and AACD. It is hardly conceivable to make a more persuasive case for the centrality and causative role of Aβ in AD. It follows that these two attributes, i.e., the centrality and the causative role of Aβ are requisite for *any* theory of AD. At first glance, this statement seems to contradict the concluding remark of the preceding section. The two notions, however, are not mutually exclusive. Considerations of the preceding section indeed rule out the causative role of extracellular Aβ but not that of the another pool of amyloid-beta: *intraneuronal* Aβ, *i*Aβ. 

The causative role of *i*Aβ is the central tenet of the ACH2.0, which envisions AD as a two-stage disease. In the first, asymptomatic stage, AβPP-derived Aβ accumulates, in a decades-long process, to critical levels that cause the activation of the second, devastating AD stage that is anchored and driven by an agent which is independent of AβPP and which sustains and perpetuates its own production [1,2]. In terms of the ACH2.0, this agent is *i*Aβ generated in the AβPP-independent pathway [1]. Potentially, as discussed in [2], the agent driving the stage two of AD can be other than *i*Aβ. However, because all known AD-associated mutations affect Aβ, it is highly plausible that this agent is, in fact, *i*Aβ. It appears, therefore, that *i*Aβ, differentially produced in two distinct, albeit related, pathways, runs the entire course of the disease. Two key features of the *i*Aβ driving the second, symptomatic, AD stage are suggested by the following empirical data. (a) Since suppression of the production of AβPP-derived Aβ during the symptomatic stage of AD has no effect whatsoever on the progression of AD [11,12], this *i*Aβ pool must be produced in the AβPP-independent pathway. (b) Since the depletion of extracellular Aβ in human clinical trials showed no efficacy whatsoever [11,12], the bulk of Aβ produced in the AβPP-independent pathway must be retained intraneuronally. The physiological occurrence of cellular mechanisms capable of generating Aβ independently of AβPP (summarized in Section 22, Section 23 and Section 24 of the present study) provides additional support to the above notions.

The crucial role of the AβPP proteolytic/secretory pathway in only the first, pre-symptomatic stage of AD explains why drugs targeting extracellular AβPP-derived Aβ or its production by the AβPP proteolysis did not and indeed *could not* have any effect on the progression of the disease (driven at this stage by *i*Aβ produced independently of AβPP) in symptomatic AD patients despite effectively fulfilling their mechanistic purpose. By the same logic, the overall success of the same drugs in animal models suggests that no second AD stage occurs there, consistent with the inability to obtain full spectrum of AD pathology in those experimental systems (reviewed in [1] and further discussed below). On the other hand, the ACH2.0 predicts that, if administered pre-symptomatically, prior to the activation of the AβPP-independent *i*Aβ production pathway, these drugs could be effective preventively by precluding AβPP-derived Aβ from reaching the levels triggering the second AD stage [1,2]. The results obtained in clinical trials of lecanemab and donanemab substantiate this notion [3].

## 4. Two Sources of AβPP-Derived Intraneuronal Aβ

As discussed above, in the framework of the ACH2.0, it is assumed that in the second, symptomatic AD stage, the bulk, or the entire output, of the AβPP-independent *i*Aβ production pathway is not secreted but retained within the cell [1,2]; this stimulates and perpetuates its own production and thus drives the disease [1]. As for the sources of AβPP-derived *i*Aβ, crucial in the first, asymptomatic AD stage, those are well understood and are briefly summarized as follows.

### 4.1. Influx of iAβ via the Uptake of Extracellular Amyloid-β

Multiple studies of the role of *i*Aβ in AD indicated that it is the major trigger of AD pathology [21,22,23,24,25,26,27,28,29,30,31,32]. They also showed that the levels of *i*Aβ, rather than those of Aβ plaques, correlate with the loss of neurons in studied systems [33]. As for how AβPP-derived Aβ accumulates intraneuronally, there are two recognized venues. The first venue is the well-documented importation of extracellular Aβ by the cell. Soluble Aβ was shown to be taken up inside the cell by endocytosis [34]. Importantly, extracellular Aβ_42_ is imported twice as efficiently as the other species of extracellular soluble Aβ [35]. The more efficient uptake of Aβ_42_ leads to higher rates of its accumulation as *i*Aβ and, in combination with its augmented cytotoxicity (apparently due to its increase propensity to aggregate), appears to play a decisive role in the occurrence of FAD in carriers of mutations, both in AβPP and in presenilins (PSEN), resulting in the elevated production of Aβ_42_ versus other Aβ species [36]. Multiple studies suggested that oligomerization of extracellular Aβ is a precondition for its importation inside the cell [37,38,39], a notion consistent with the increased cytotoxicity of extracellular Aβ in oligomeric conformation [34]. Aβ_42_ was shown to form oligomeric structures and thus to enter the cell significantly more efficiently than other Aβ species [37,38]. The importation of extracellular Aβ is mediated by and was demonstrated to be dependent on the isoform of ApoE expressed by the cell [38]. Interestingly, ApoE4 appears to be significantly more effective in facilitating the importation of extracellular Aβ than other ApoE isoforms [26,38]. Importantly, this particular ApoE isoform is also the major risk factor for the occurrence of AD, consistent with the proposed role of *i*Aβ in the disease. Cellular uptake of extracellular Aβ was also shown to be facilitated by LRP [40], by the α7 nicotinic acetylcholine receptor [41,42,43], by the RAGE (receptor for advanced glycation) [44,45,46], by the FPRL1 (formyl peptide receptor-like1) [47], and by NMDA (N-methyl-d-aspartate) receptors [48]. While its importation occurs also in normal individuals [49], the relative efficiency of its cellular uptake appears, as discussed below, to play a significant role in the occurrence of AD.

### 4.2. A Fraction of the C99 Fragment of AβPP Undergoes the Gamma-Cleavage on Intracellular Membranes; the Resulting Aβ Is Retained Intraneuronally

The second venue for the occurrence of *i*Aβ is the intracellular retention of a fraction of Aβ generated by AβPP proteolysis. The occurrence of this phenomenon is dependent on the site where the gamma-cleavage of the C99 fragment of AβPP takes place. The bulk of these cleavages occur on the plasma membrane, with the resulting Aβ being exported into the extracellular space. On the other hand, gamma-secretase cleavages have been also documented on intracellular membranes [50,51,52], with the resulting Aβ being retained intraneuronally. The locations of such cleavages include the lysosomes, mitochondria, endosomes, Golgi apparatus as well as the TGN (trans-Golgi network), and the ER (endoplasmic reticulum). Cleavages on the ER and TGN membranes appear to be specific to neuronal cells [53,54]. Gamma-cleavages at these locations produce, apparently, different species of iAβ. Those occurring on the ER membranes generate mostly *i*Aβ_42_ while those taking place on the TGN membranes produce iAβ enriched in Aβ_40_ species [53,54,55,56,57,58].

Given that AD is caused by *i*Aβ, it could be predicted that the increase in the proportion of Aβ produced on intracellular membranes and thus retained as *i*Aβ would facilitate the occurrence of AD. This is exactly what occurs in carriers of the Swedish FAD mutation. This mutation significantly increases a fraction of AβPP/C99 processed at the intracellular membrane locations [59]. This results in the elevated levels of *i*Aβ and, consequently triggers FAD [59]. In another example where the above prediction is borne out, certain PSEN mutations facilitate gamma-cleavage on intracellular membranes and thus cause the elevation in *i*Aβ levels and the occurrence of FAD [60].

To summarize, a compendium of empirical data referenced above is presented below. It is either consistent with or is strongly indicative of the notion that AβPP-derived *i*Aβ causes AD and drives its first stage:

1. AD is caused by Aβ and not by tau protein. The mutations of the former lead to the pathological formation of tau tangles and the disease; the reverse does not occur [20,61].
2. The correlation between levels of extracellular Aβ and AD is poor:
  (a) The excessive deposition of extracellular Aβ is often not accompanied by AD [13,14,15,16,17,18,19].
  (b) The occurrence of AD (as judged by cognitive symptoms as well as by PET scan and postmortem analysis) is not always accompanied by excessive deposition of extracellular Aβ [20].
3. A fraction of Aβ produced in the AβPP proteolytic pathway was shown to physiologically accumulate within neurons via two defined mechanisms [21,22,23,24,25,26,27,28,29,30,31,32,33,34,35,36,37,38,39,40,41,42,43,44,45,46,47,48,49,50,51,52,53,54,55,56,57,58,59,60]; discussed above.
4. AD is associated with multiple factors that enable and promote the intraneuronal accumulation of *i*Aβ produced in the AβPP proteolytic pathway:
  (a) Cytotoxic Aβ_42_ is taken up by the cell twice as efficiently as other isoforms of Aβ [35];
  (b) ApoE4, a major AD risk factor, is significantly more efficient in the internalization of extracellular Aβ than other species of ApoE [26,38];
  (c) The “toxicity” of extracellular Aβ in oligomeric form is due to its efficient cellular uptake [34,37,38,39].
5. Mutations that either cause AD or that protect from AD were shown to interfere with accumulation of *i*Aβ:
  (a) The Swedish AβPP mutation that causes familial AD was shown to expedite AβPP processing on internal membranes and thus to increase the retention of AβPP-derived Aβ within neurons [59];
  (b) The Flemish AβPP, FAD-causing, mutation elevates levels of *i*Aβ by suppressing physiologically occurring BACE2-mediated *i*Aβ cleavage [62];
  (c) The Icelandic AβPP mutation that protects from both AD and AACD decreases levels of *i*Aβ by significantly increasing the efficiency of BACE1-mediated *i*Aβ cleavage [4,5];
  (d) Certain PSEN FAD-causing mutations enhance the cellular uptake of extracellular Aβ by shifting the gamma-cleavage to position 42 of Aβ, thus elevating the proportion of Aβ_42_ produced in the AβPP proteolytic/secretory pathway [36];
  (e) Some PSEN FAD-causing mutations increase the retention of Aβ produced in the AβPP proteolytic pathway within neuronal cells by facilitating the gamma-cleavage of C99 on internal (rather than on plasma) membranes [60].
6. The correlation between levels of *i*Aβ in AD-affected neurons and the incidence of AD biomarkers was shown to be good [33,49].
7. The results of preclinical and human clinical trials indicate that AβPP-derived *i*Aβ drives the first stage of AD but plays no significant role in the second stage of the disease [8,9,10,11,12].

## 5. Mechanistic Aspects of the ACH2.0

As was mentioned above, the Amyloid Cascade Hypothesis 2.0 posits that AD is a two-stage disease [1,2]. The first stage is a slow, decades-long accumulation of AβPP-derived *i*Aβ. Upon reaching a critical threshold, it mediates the activation of the second AD stage, which is relatively (to the first stage) fast. More precisely, it triggers the initiation of the pathway that generates an agent, which drives the second AD stage. This agent is presumed to be capable of (a) anchoring a cascade that includes tau pathology and leads to neuronal death and (b) sustaining its own production [2]. It could be argued that the second requirement is redundant in view of the continuous influx of AβPP-derived *i*Aβ. However, this is not the case, because suppression of the AβPP proteolytic pathway at symptomatic phase of the disease (i.e., at the stage two of AD) in human clinical trials had no effect whatsoever on the progression of AD [11,12], consistent with the autonomous operation of the pathway that produces an agent which drives the second AD stage. As argued above, it is highly plausible that the second AD stage-driving agent is *i*Aβ generated in the AβPP-independent pathway; it is at the heart of Alzheimer’s pathology, and it is of great interest how AβPP-derived *i*Aβ triggers its production. Plausibly, this occurs via the elicitation of the integrated stress response, ISR (although additional or alternative pathways cannot be excluded) [1,2].

### 5.1. Plausible Involvement of the Integrated Stress Response in Generation of an Agent Driving the Second Stage of AD

The integrated stress response, ISR, is a signaling cascade that takes place in eukaryotic cells and is triggered by a large variety of cellular stresses [63,64,65,66,67,68,69,70,71,72]. It is termed “integrated” because all events that initiate it lead to a common and central occurrence: the activation of eIF2α (a subunit of eukaryotic translation initiation factor 2) by its phosphorylation at a specific site (serine 51). Four kinases (comprising the family of eIF2α kinases) are capable of enacting this phosphorylation/activation event. They are PKR, PERK, GCN2, and HRI. Phosphorylation of eIF2α elicits the ISR. This manifests itself as an acute decline in the protein synthesis output. The reduction in the global cellular protein synthesis occurs via the suppression of the cap-dependent initiation of translation. Concurrently, the ISR promotes cap-independent translation of selected mRNA species; among those are mRNAs encoding specific transcription factors. The ISR-induced transcription factors, or translation products of the genes activated by these factors, may include components critical for the activation of the AβPP-independent *i*Aβ production or, alternatively, of the pathway generating the agent (if other than *i*Aβ) which drives the second stage of AD [1,2].

### 5.2. PKR Kinase Is Activated by iAβ and, in Turn, Elicits the ISR and Triggers the Second Stage of AD

In the majority of human population, AβPP-derived *i*Aβ does not reach the ISR-eliciting levels within the human lifespan and no AD occurs. On the other hand, when it does reach the ISR-eliciting levels, it mediates the elicitation of the integrated stress response. Elicitation of the ISR in AD can occur via two distinct pathways. In the first pathway, the ISR is triggered by the *i*Aβ-mediated activation of the PKR kinase. Indeed, experimental data obtained with both, established human cell lines and with human primary neuronal cells demonstrated the activation of PKR by Aβ cytotoxicity [73]. These results were corroborated in experiments with animal models exogenously overexpressing Aβ [74,75]. A linkage between PKR and AD in human patients was established by showing that degenerating neuronal cells are positive for both activated PKR and eIF2α (indicating that phosphorylated PKR has elicited the ISR in these cells and plausibly contributed to their degeneration via the ISR-triggered apoptotic pathway) [76,77]. As to how *i*Aβ triggers the phosphorylation and activation of PKR, experiments with animal models indicated that this might occur through TNFα [78]. Alternatively, PACT (PKR activator) could mediate the interaction between *i*Aβ and the kinase; this possibility was suggested by the observation of the co-localization of PACT and activated PKR in degenerating human neurons [79]. *i*Aβ-mediated activation of PKR through the elevation of PACT levels was also indicated by its observed suppression in human neuroblastoma cells following the cells’ exposure to PACT shRNA [79].

### 5.3. HRI Kinase Activation Is Triggered by iAβ-Mediated Mitochondrial Dysfunction; Elicitation of the ISR Follows 

The association of AD with mitochondrial distress is well established [80,81,82,83,84,85,86,87,88,89,90,91,92,93,94,95,96]. It is, in fact, one of the earliest observed events in the progression of the disease. Experimentally, the exogenous overexpression of Aβ in cell-based studies and in animal models was demonstrated to be sufficient to initiate mitochondrial distress and trigger cellular stress response. In this respect, a recent work by Brewer et al. [97] described a phenomenon whereby “long” Aβ species Aβ_45_ and Aβ_49_, generated due to incomplete activity of gamma-secretases, accumulate intraneuronally (rather than being exported). The levels of *i*Aβ_45_ in mitochondria, endosomes, and autophagosomes are significantly elevated with aging; this results in mitochondrial dysfunction [97]. For mitochondrial dysfunction to translate into the cellular stress response, the stress signal has to be transmitted from the organelle to the cytosol. Until recently, it was not entirely clear how it occurs. Studies by Guo et al. [98] and by Fessler et al. [99] provided the answer. They demonstrated that mitochondrial distress activates the mitochondrial protease OMA1. In turn, the activated OMA1 cleaves another mitochondrial protein, DELE1. One of the resulting fragments of DELE1 is exported into the cytosol. There, it binds to the HRI kinase; this results in the phosphorylation and activation of HRI, thus continuing the cascade and leading, through the phosphorylation eIF2α, to the elicitation of the ISR. Data indicate that this signaling pathway is operative in neuronal cells [99].

## 6. Self-Perpetuating, AβPP-Independent Generation of *i*Aβ: The Engine That Drives the Second Stage of AD

In light of the above considerations and to simplify further discussion, the present study provisionally assumes that the agent driving the second AD stage is *i*Aβ produced in the AβPP-independent manner. This is a “safe” assumption because (a) it is highly plausible that this is the case and (b) if the agent in question is not *i*Aβ, neither the logic of the thesis nor the underlying concepts would be affected [2]; in essence, any potential agent would have to be able to drive the AD pathology and to sustain and perpetuate its own production pathway. 

**Figure 1:**
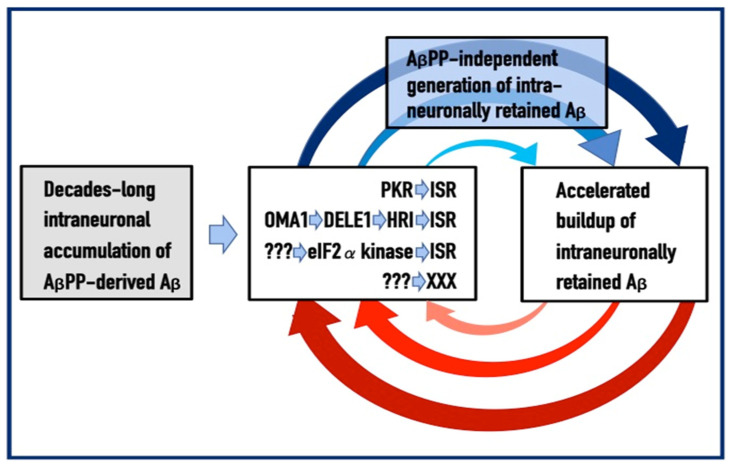
Mechanistic aspects of *i*Aβ dynamics in the ACH2.0 perspective: The AD Engine. *Left box*: The life-long accumulation of intraneuronal Aβ (*i*Aβ) produced in the AβPP proteolytic pathway. Two distinct processes contribute to this accumulation: importation of secreted extracellular Aβ inside the cell and retention within the neuron of a fraction of Aβ generated by gamma-cleavage of the C99 fragment of AβPP on intracellular rather than on plasma membranes. Such accumulation of *i*Aβ is a normal physiological process common to healthy individuals and future AD patients. It becomes detrimental if and when it reaches the critical threshold and activates the second, symptomatic stage of AD. In the majority of population this threshold is not reached within the lifespan of an individual and no AD occurs. *Middle box*: When *i*Aβ, accumulated in a life-long process, reaches the critical threshold invoked above, it mediates the elicitation of the integrated stress response, ISR (or of a yet undefined pathway marked XXX). This occurs via the documented activation of two eIF2α kinases, PKR and HRI (other eIF2α kinases, or yet unidentified mediators denoted “???” could be also involved). Activated PKR and/or HRI phosphorylate eIF2α and thus trigger the ISR. *Top box*: The ISR manifests itself as an acute decline in the protein synthesis output. The reduction in the global cellular protein synthesis occurs via the suppression of the cap-dependent initiation of translation. Concurrently, the ISR promotes cap-independent translation of selected mRNA species; among those are mRNAs encoding specific transcription factors. The ISR-induced transcriptions factors, or translation products of the genes activated by these factors, plausibly include components critical for the activation of the AβPP-independent *i*Aβ generation pathway. The bulk, if not the whole *i*Aβ output is retained within affected neurons. *Right box*: The increased influx of *i*Aβ generated in the AβPP-independent manner substantially elevates its steady-state levels. *Arched arrows*: As the result of a drastic augmentation of *i*Aβ levels, pathways leading to the elicitation of the integrated stress response are sustained, and the activity of the AβPP-independent *i*Aβ generation pathway and uninterrupted influx of *i*Aβ are perpetuated. These continuous cycles of *i*Aβ-stimulated propagation of its own production constitute an engine that drives AD, the AD Engine. Only when the AD Engine is activated does the disease commence. A possibility that the agent driving the second AD stage is not *i*Aβ cannot be excluded; this would not, however, change the logic of the thesis and is discussed in detail in [2].

To summarize the preceding sections, it has been proposed that in the first AD stage, AβPP-derived *i*Aβ accumulates, in a decades-long process, to the critical over-the threshold levels. This leads to the activation of the PKR kinase and, through mitochondrial distress, of the OMA1-DELE1-HRI signaling pathway. Consequently, eIF2α is phosphorylated and the integrated stress response elicited. The induction of certain genes’ expression within the framework of the ISR provides crucial component(s) required for the operation of the AβPP-independent Aβ generation pathway and causes its activation. The product of this pathway is, in fact, *i*Aβ (i.e., intraneuronal rather than extracellular Aβ) because the majority of it, if not the complete output of the pathway, is not secreted but, instead, is retained within the neurons. The substantially increased influx of *i*Aβ rapidly elevates its steady-state levels; pathways leading to the elicitation of the integrated stress response are sustained, and the activity of the AβPP-independent *i*Aβ generation pathway, and, consequently, uninterrupted influx of *i*Aβ, are perpetuated. These continuous cycles of the *i*Aβ-stimulated propagation of its own production constitute an engine that drives the disease, the AD Engine. Its operation is illustrated in Figure 1.

The decades-long accumulation of AβPP-derived *i*Aβ (left box in Figure 1) leading to the activation of the AβPP-independent *i*Aβ production and, consequently, of the AD Engine is referred hereafter as “the first stage of AD”. It should be emphasized, however, that it becomes such only post-factum, provided the disease actually occurs. Otherwise this is a normal physiological process common to healthy individuals and future AD patients. Only if and when AβPP-derived iAβ reaches the critical threshold and the AβPP-independent Aβ generation pathway and, consequently, the self-sustaining AD Engine (arched arrows-connected boxes in Figure 1) is activated (referred to henceforth as the symptomatic “second stage of AD”) does the disease commence, and “the first stage of AD” earns its name. In this terminology, therefore, “the second stage of AD” is synonymous with “AD”; both terms are used interchangeably below.

Since the symptomatic stage, i.e., stage two of AD, requires the activation of the AβPP-independent *i*Aβ production pathway and, consequently, of the AD Engine, factors determining the attainment of the critical AD Engine-activating threshold by AβPP-derived *i*Aβ play the key role in defining the susceptibility to AD. These factors and the dynamics of AβPP-derived iAβ accumulation are discussed in detail in the following sections below. 

## 7. The Dynamics of Aβ Accumulation and of the Disease in AD-Affected Human Population: Comparison of the ACH and ACH2.0 Perspectives

**Figure 2:**
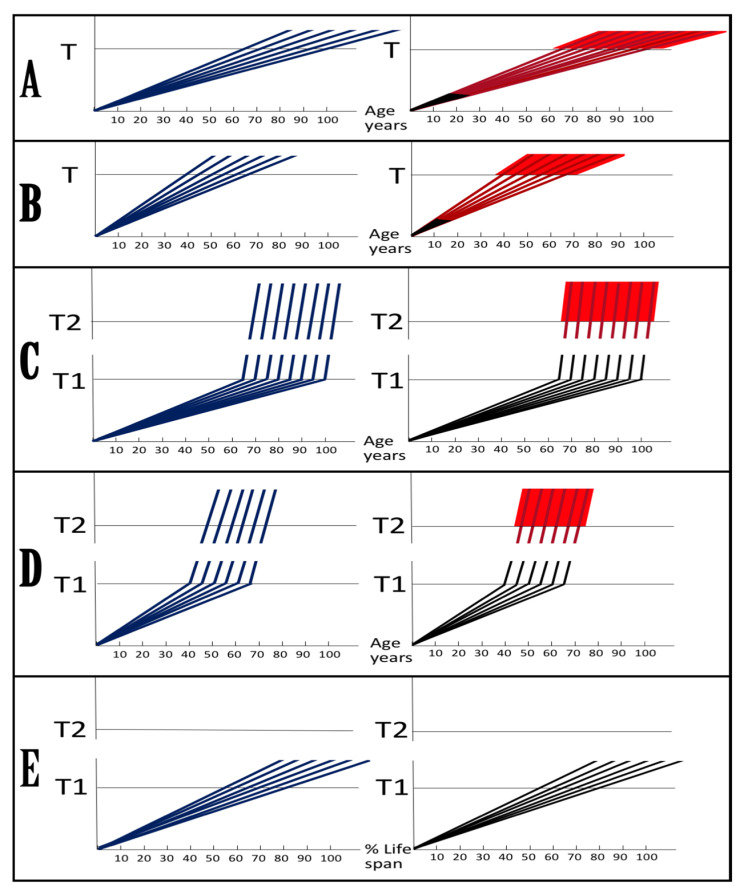
Dynamics of Aβ accumulation and the disease in AD-affected population: ACH and ACH2.0 perspectives. *Blue lines*: Levels of Aβ (panels **A**,**B**) or *i*Aβ (panels **C**–**E**). *Red lines*: Degree of neuronal damage. *Black lines*: Indicator lines; no noticeable neuronal damage. *Red blocks*: Apoptotic zone. Threshold **T**: The level of Aβ and the consequent level of neurodegeneration causing symptomatic manifestation of AD. Threshold **T1**: The level of AβPP-derived *i*Aβ triggering cellular processes leading to the activation of the AβPP-independent generation of *i*Aβ. Threshold **T2**: The level of *i*Aβ and the consequent degree of neurodegeneration causing cellular commitment to apoptosis and acute AD symptoms. Panels **A**,**B** (SAD, FAD respectively): Dynamics of AD in the ACH perspective. Extracellular Aβ accumulates and the degree of neuronal damage increases proportionally. When the **T** threshold is crossed, the symptomatic AD stage commences. Panels **C**,**D** (SAD, FAD respectively): Dynamics of AD in the ACH2.0 perspective. Following crossing of the **T1** threshold by *i*Aβ produced in the AβPP proteolytic pathway, its generation in the AβPP-independent pathway commences. Since the entire Aβ output of the AβPP-independent pathway is retained intraneuronally, the rate of *i*Aβ accumulation greatly accelerates and its levels substantially and rapidly increase, which causes, via the cascade involving tau pathology, significant neuronal damage and triggers initial AD symptoms. When *i*Aβ, and the consequent degree of neuronal damage reach and cross the **T2** threshold, the cell apoptotic pathway is triggered and acute AD symptoms manifest. (Panel **E**)*: iAβ dynamics in subjects (including non-human mammals) with an inoperative second stage.* AβPP-derived *i*Aβ reaches and crosses the **T1** threshold but the AβPP-independent *i*Aβ generation pathway remains inoperative. Neither levels of *i*Aβ causing AD-related damage nor the **T2** threshold are reached, no AD occurs.

As described in the preceding sections, in the framework of the ACH2.0 the kinetics and even the occurrence of AD depend on the accumulation of *i*Aβ. Only when it reaches the critical threshold and triggers the activation of its own production in the AβPP-independent mode does the symptomatic stage of the disease commence. If this threshold is not reached within the lifetime of an individual, no AD occurs; in fact, this is what happens in the majority of the human population. The following sections below are concerned with the dynamics of *i*Aβ accumulation and, consequently, of the disease. In this respect, it is instructive to compare these dynamics in two paradigms: the ACH and the ACH2.0. Both presume that the agent that drives AD is Aβ. The similarity, however, ends here. Whereas the ACH is centered on extracellular Aβ produced and secreted in the AβPP proteolytic pathway, in the ACH2.0, the disease is caused by *i*Aβ. No less importantly, the dynamics of *i*Aβ, or of extracellular Aβ in the case of the ACH, accumulation in the two paradigms are radically distinct. These dynamics are diagrammatically depicted in Figure 2 (each continuous line denotes an individual AD patient).

Panel **A** (sporadic AD) and panel **B** (FAD) illustrate the ACH-based interpretation of AD. Blue lines represent the kinetics of accumulation of secreted extracellular Aβ, whereas red lines represent the kinetics of neurodegeneration; both are single-phased. The kinetics of the neuronal damage can be further separated into two stages, asymptomatic (red lines) and symptomatic (red blocks). The latter commences when the levels of secreted Aβ and the corresponding extent of neurodegeneration reach and cross the threshold **T**. In this paradigm, Aβ is generated exclusively by AβPP proteolysis and is secreted outside the cell. As it accumulates, it causes neuronal damage commencing early in life; the extent of this damage is proportional to the extent of extracellular Aβ accumulation. Neuronal damages accrue with time and manifest as AD symptoms late in life in cases of SAD, starting at the mid-sixties, or much earlier in cases of FAD, where levels of extracellular Aβ reach the **T** threshold sooner. In its symptomatic stage, the disease is deemed untreatable. There are presently no preventive treatments for AD. In the ACH paradigm, were such treatments to exist, they would be fruitless late in life: even if the AD symptoms did not yet manifest, the irreversible neurodegeneration already occurred.

In the ACH2.0 paradigm, the principal feature of the dynamics of *i*Aβ accumulation and of the disease (SAD: panel **C**; FAD: panel **D**) is that both are biphasic. In the first stage, Aβ is produced exclusively by AβPP proteolysis. *i*Aβ is derived only from AβPP and accrues very slowly, over the bulk of individual’s lifespan. Its accumulation occurs through the importation of extracellular Aβ inside the cell and via the intraneuronal retention of Aβ generated by gamma-cleavage of C99 on intracellular membranes. Both processes, and the resulting accumulation of *i*Aβ, are normal physiological occurrences and take place not only in future AD patients but also in healthy individuals, as well as in non-human mammals. Until and unless the **T1** threshold is crossed, there is no significant neurodegeneration and no disease at this stage (black indicator lines). The second stage commences when levels of *i*Aβ produced in the AβPP proteolytic pathway reach and cross the **T1** threshold. This triggers cellular processes culminating in the initiation of AβPP-independent production of *i*Aβ, consequent activation of the AD Engine, commencement of the second AD stage, and symptomatic manifestation of AD. This stage appears to be exclusive to humans or at least species-specific, probably because the AβPP-independent *i*Aβ production pathway is (see below).

Since the entire Aβ output of the AβPP-independent pathway is retained intraneuronally, in the second AD stage the rate of *i*Aβ accumulation greatly accelerates and its levels substantially and rapidly increase, which causes, via the cascade involving tau pathology, significant neuronal damage and triggers the initial AD symptoms (red lines). When *i*Aβ, and the consequent degree of neuronal damage reach and cross the **T2** threshold, the irreversible apoptotic pathway is triggered (red blocks), and acute AD symptoms manifest. In contrast to the ACH-based interpretation of the disease, in the ACH2.0 paradigm preventive treatment, if and when available, would be successful at any time before the second phase is initiated. If the latter were precluded, e.g., via suppression of the AβPP-independent *i*Aβ generation pathway (panel E), no AD would occur; this is the scenario that plays out in some, or possibly in all, non-human mammals. 

## 8. The *i*Aβ Dynamics in the Affected Neuronal Population of an Individual Patient in the ACH2.0 Perspective

**Figure 3:**
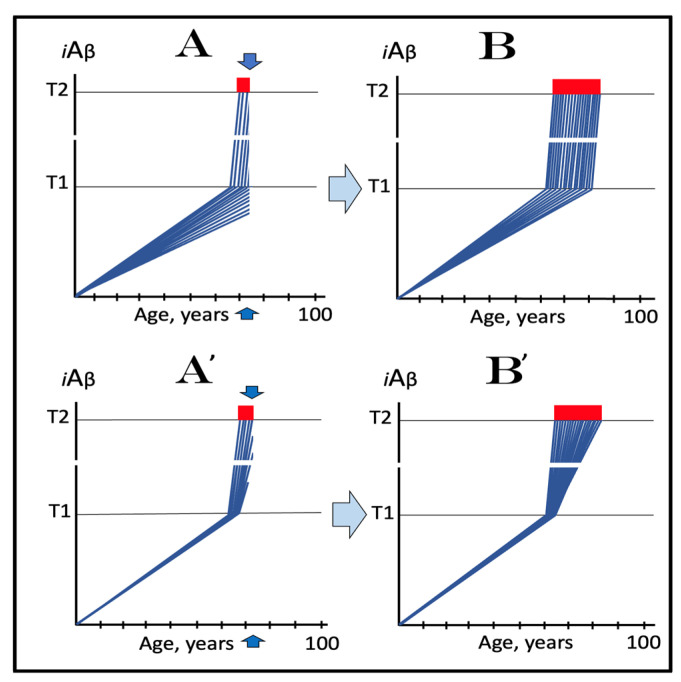
*i*Aβ dynamics in the affected neuronal population of an individual patient in the ACH2.0 perspective. *Blue lines*: Levels of *i*Aβ in individual AD-affected neurons. Threshold **T1**: The level of AβPP-derived *i*Aβ triggering cellular processes leading to the activation of the AβPP-independent generation of *i*Aβ. Threshold **T2**: The level of *i*Aβ and the consequent degree of neurodegeneration causing cellular commitment to apoptosis and acute AD symptoms. *Red blocks*: Apoptotic zone. *Vertical blue arrows*: Commencement of the occurrence of AD symptoms. *Panel* ***A***: Individual neurons reach and cross the **T1** threshold with a stochastic distribution within a broad time interval, which primarily determines the duration of the disease. Subsequent to the **T1** threshold crossing by AβPP-derived *i*Aβ, the AβPP-independent *i*Aβ generation pathway is activated, the rate of *i*Aβ accumulation and its cellular levels are sharply elevated, and neuronal damage rapidly increases. Following crossing of the **T2** threshold, neurons enter the apoptotic pathway and are ultimately lost. When sufficient fraction of neurons lose their functionality or die, AD symptoms manifest while a substantial proportion of affected neurons have not yet crossed the **T1** threshold. *Panel* ***B***: With the progression of the disease, additional neurons cross first the **T1** and then the **T2** thresholds and the disease reaches its end stage. *Panel* ***A’***: The neuronal crossing of the **T1** threshold occurs within relatively short time interval. Subsequent to the crossing of the **T1** threshold, the affected neurons advance toward and cross the **T2** threshold in a broad stochastic distribution; the temporal duration of this distribution determines the duration of the disease. When the neuronal damage or loss occurred to a degree sufficient for symptomatic manifestation of the disease, the majority, if not the entire population, of the affected neurons already crossed the **T1** threshold. *Panel* ***B’***: As the disease progresses, more neurons reach the **T2** threshold and enter the apoptotic pathway; eventually, the end stage is reached.

The dynamics of *i*Aβ accumulation and of the disease in AD-affected human population, discussed in the preceding section, presents a “coarse-grained” picture of the evolution of AD. To gain a better understanding, one should consider the *i*Aβ dynamics in the affected neuronal population of an individual AD patient; it’s understanding could be instrumental in defining therapeutic strategies applicable in the ACH2.0 framework. Two principal, conceptually distinct, versions of such dynamics could be envisaged in the ACH2.0 perspective. In one version, illustrated in Figure 3, panels **A** and **B** (every continuous blue line represents a single affected neuron), individual neurons reach and cross the **T1** threshold with a stochastic distribution within a broad time interval, which primarily determines the duration of the disease. Subsequent to the **T1** threshold crossing by AβPP-derived *i*Aβ, the AβPP-independent *i*Aβ generation pathway and, consequently, the AD Engine are activated, the rate of *i*Aβ accumulation and its cellular levels are sharply elevated, and neuronal damage rapidly increases. Following crossing of the **T2** threshold, neurons enter the apoptotic pathway and are ultimately lost. When a sufficient fraction of neurons lose their functionality or die, acute AD symptoms manifest, as shown in panel **A** of Figure 3. With the progression of the disease, additional neurons cross first the **T1** and then the **T2** thresholds, and the disease reaches its end stage, as shown in panel **B** of Figure 3.

However, in the face of the empirical data, this scenario is, apparently, unviable. Indeed, as shown in panel **A** of Figure 3, when a fraction of affected neurons reaches the **T2** threshold and AD symptoms manifest, a large proportion of affected neurons have not yet crossed the **T1** threshold. If, at this time, the generation of Aβ in the AβPP proteolytic pathway were repressed, for example, by BACE1 inhibitors shown capable of effectively achieving this [11,12], the accumulation AβPP-derived *i*Aβ should be slowed. Consequently, crossing of the **T1** threshold by the affected neurons should also be slowed or precluded, and the progression of AD should be, likewise, slowed or arrested. However, in human clinical trials of verubecestat, these effects were seen neither with mild to moderate [11] nor with prodromal patients [12] despite the observed strong inhibition of Aβ production in the AβPP proteolytic pathway.

Another version of *i*Aβ dynamics in the affected neuronal population of individual AD patient is illustrated in Figure 3, panels **A**’ and **B**’. The main feature, suggested by experimental data [11,12] and distinguishing this version from the above described version, is the duration of the neuronal crossing of the **T1** threshold: here, it occurs within relatively short time interval. Subsequent to the crossing of the **T1** threshold, the affected neurons advance toward and cross the **T2** threshold in a broad stochastic distribution; the temporal duration of this distribution determines the duration of the disease in this version. Importantly, when the neuronal damage and/or loss occurred to a degree sufficient for symptomatic manifestation of the disease, the majority, if not the entire population, of the affected neurons, have already crossed the **T1** threshold, as shown in Figure 3, panel **A**’. Since, subsequent to the **T1** threshold crossing, the AβPP-independent *i*Aβ generation pathway is activated in all or nearly all affected neurons, BACE1 inhibitors (or drugs targeting the accumulation of AβPP-derived *i*Aβ) would be rendered therapeutically ineffective, a notion that was indeed corroborated by the empirical data [11,12].

As the disease progresses, more neurons reach the **T2** threshold and enter the apoptotic pathway; eventually, the end stage is reached, as shown in panel **B**’ of Figure 3. The above two versions of *i*Aβ dynamics in the affected neuronal population are just basic outlines of the process. Intermediary versions that combine features of the two basic scenarios can also be envisioned. However, the essential requirement of any version is that by the time AD symptoms manifest, the levels of *i*Aβ produced in the AβPP proteolytic pathway have already crossed the **T1** threshold, and the second AD stage has commenced in the majority of affected neurons.

## 9. The Dynamics of AD in the ACH2.0 Perspective: Effect of the Rate of Accumulation of AβPP-Derived *i*Aβ in the Affected Neuronal Population of an AD Patient

As discussed above, in the framework of the ACH2.0, the “second stage of AD” is, de facto, symptomatic AD, i.e., the stage where AD-causing, *i*Aβ-mediated neurodegeneration actually takes place. Moreover, as argued in the preceding sections, the “first AD stage” becomes such only post-factum, if and when AβPP-derived *i*Aβ levels cross the **T1** threshold and the disease actually occurs. Otherwise, it is a normal physiological process shared by healthy individuals and (future) AD patients; if the stage two of AD, i.e., the self-perpetuating AβPP-independent *i*Aβ generation pathway, is “cancelled”, as depicted in the panel **E** of Figure 2 above, and only the AβPP proteolytic pathway remains operational, there could be no AD because AβPP-derived *i*Aβ would not reach AD neurodegeneration-causing levels within the lifespan of an individual. The second AD stage (synonymous with AD, as defined above) commences when the levels of AβPP-derived *i*Aβ reach and cross the **T1** threshold and trigger the activation of the AβPP-independent *i*Aβ production pathway and the consequent operation of the self-sustaining AD Engine. Accordingly, in the ACH2.0 perspective the susceptibility to AD is defined by two factors, which determine the timing of the **T1** crossing by AβPP-derived *i*Aβ: the rate of accumulation of AβPP-derived *i*Aβ and the extent of the **T1** threshold. In the present section, we consider the effect of the former in SAD (or prospective SAD) with a given, relatively low, extent of the **T1** threshold (and, consequently, insignificant extent of AβPP-derived *i*Aβ accumulation-related cell damage prior to the **T1** threshold’s crossing), within temporal boundaries of the symptomatic stage between 65 and 100 years of age (the former reflects a statistical age of the onset of SAD and the latter is a reasonable cut-off, i.e., the assumed lifespan; increasing this number would not change the logic but would make the figure more cumbersome). The present section analyzes only the effect of the rate of accumulation of AβPP-derived *i*Aβ on its crossing (or not crossing) the **T1** threshold and the consequent occurrence of AD. This analysis is not concerned with the rate of accumulation of *i*Aβ (mostly *i*Aβ generated independently of AβPP) in the second AD stage. The latter does not impact the former in any way, and its effect on the progression of the second AD stage is discussed below (Section 20).

**Figure 4:**
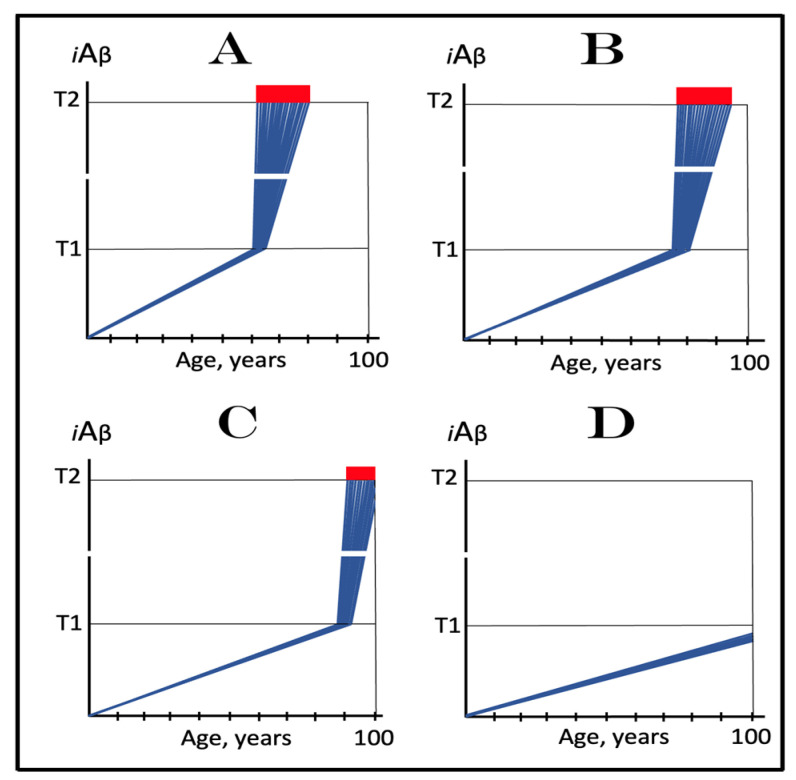
Dynamics of AD: Effect of the rate of accumulation of AβPP-derived *i*Aβ. *iAβ*: Intraneuronal Aβ levels; ***T1***: The level of *i*Aβ that triggers elicitation of the integrated stress response, initiation of AβPP-independent generation of *i*Aβ, and activation of the AD Engine. ***T2***: The level of *i*Aβ that triggers cell’s commitment to the apoptotic pathway. *Red blocks*: Fraction of affected neurons either committed to apoptosis or dead. The **T1** threshold is constant and is chosen deliberately low, so that the extent of AβPP-derived *i*Aβ-accumulation-related neuronal damage prior to the **T1** threshold’s crossing is insignificant and inconsequential. The Figure does not consider the effect of the rate of *i*Aβ (mostly *i*Aβ generated independently of AβPP) accumulation in the second AD stage, which is assumed constant for purposes of this analysis. The lifespan is assumed to end at 100 years of age. In *panel **A***, the rate of AβPP-derived *i*Aβ accumulation is such that AD symptoms manifest at about 65 years of age (statistical age of the commencement of AD). As the rate of AβPP-derived *i*Aβ accumulation decreases, the timing of its reaching and crossing the **T1** threshold, and consequently of the commencement of stage two of AD, increases. In *panel **B***, this timing is such that AD symptoms manifest at about 85 years of age. In *panel **C***, AβPP-derived *i*Aβ crosses the **T1** threshold and initiates the AβPP-independent *i*Aβ production pathway so late that, while the manifestation of AD symptoms commences, the disease does not run its complete course within the lifespan of an individual. In *panel* **D**, the rate of AβPP-derived *i*Aβ accumulation is sufficiently low for it not to reach the **T1** threshold within the lifespan of an individual. Therefore, the depicted process is, in contrast to the analogous process in panels **A** through **C**, *not* “the first stage of AD”. Note that given a sufficient lifespan, AβPP-derived *i*Aβ would eventually cross the **T1** threshold and AD would inevitably occur.

Figure 4 illustrates the effect of the rate of AβPP-derived *i*Aβ accumulation on the timing of the commencement of the second AD stage. In panel **A**, the rate is such that AD symptoms manifest at about 65 years of age. As the rate decreases, the timing of AβPP-derived *i*Aβ crossing of the **T1** threshold, and, consequently, of the commencement of stage two of AD, increases. In panel **B**, this timing is such that AD symptoms manifest at about 85 years of age. In panel **C**, AβPP-derived *i*Aβ crosses the **T1** threshold and initiates the AβPP-independent *i*Aβ production pathway so late that, while the manifestation of AD symptoms commences, the disease does not run its complete course within the lifespan of an individual. In panel **D**, the rate of AβPP-derived *i*Aβ accumulation is sufficiently low for it not to reach the **T1** threshold within the lifespan of an individual. Importantly, in contrast to the analogous process in panels **A** through **C**, the process depicted in panel **D** is *not* “the first AD stage” because the **T1** threshold is not crossed and the disease does not occur. This scenario (panel **D**) describes, in fact, individuals that do not develop AD in their lifetime, the current majority of the general population. These individuals do not develop the disease not because they are “resistant” to it but because they simply run out of time, that is, the *lifetime*, to do so. The “resistance to AD” is conferred by the low rate of AβPP-derived *i*Aβ accumulation, as shown in Figure 4, or by the high extent of the **T1** threshold, as discussed in the following section, or by the combination of both; in all cases, the **T1** threshold is not reached and the second AD stage is not triggered within the lifespan of an individual. 

It is of a substantial interest that *all* known mutation(s) or factors that protect from AD have the same underlying mode of operation: they all reduce the rate of AβPP-derived *i*Aβ accumulation and prevent (or delay) the crossing of the **T1** threshold by AβPP-derived *i*Aβ within individual’s lifespan, whereas, as described below, *all* known FAD-causing mutations increase the rate of AβPP-derived *i*Aβ accumulation and accelerate the crossing of the **T1** threshold. It follows, from the above considerations, that given a sufficiently long lifespan, AD is an inevitable disorder. As a case in point, if one extrapolates the kinetics of AβPP-derived *i*Aβ accumulation in panel **D** of Figure 4, the crossing of the **T1** threshold and the commencement of the stage two of AD would occur at about 120 years of age, provided an individual in question is still alive. Such longevity is, apparently, a currently improbable and/or infrequent occurrence, but it is, possibly, attainable in the not-so-distant future. The anticipated increase in longevity would inevitably be accompanied by a corresponding increase in the incidence of AD if the population were not treated preventively. 

## 10. The Dynamics of AD and AACD in the ACH2.0 Perspective: Effect of the Extent of the T1 Threshold in the Affected Neuronal Population of an AD Patient—AACD as an Extended Segment of the Stage One of AD

**Figure 5:**
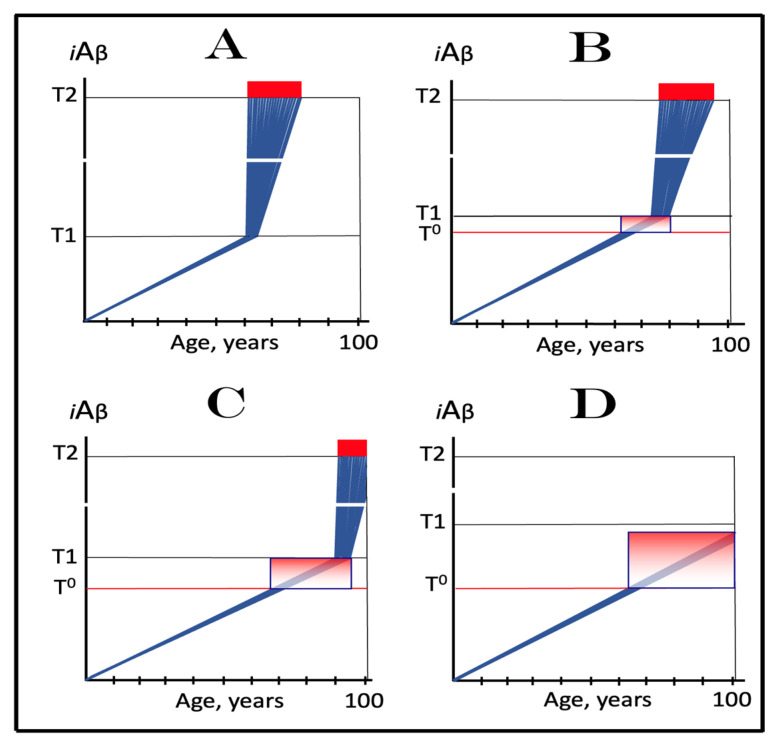
Dynamics of AD and AACD: Effect of the extent of the T1 threshold. *iA*β: Intraneuronal Aβ levels. ***T^0^***: *i*Aβ levels that trigger neuronal damage manifesting as AACD. ***T1***: *i*Aβ level that triggers elicitation of the integrated stress response, initiation of AβPP-independent generation of *i*Aβ and the activation of the AD Engine. ***T2***: *i*Aβ level that triggers cell’s commitment to the apoptotic pathway. *Red blocks*: Fraction of affected neurons either committed to apoptosis or dead. *Gradient-pink boxes*: “AACD Zone”, the differential between the **T^0^** and **T1** threshold levels of AβPP-derived *i*Aβ (more precisely, between the **T^0^** and the maximum level reached by AβPP-derived *i*Aβ short of the **T1** threshold). The rate of accumulation of AβPP-derived *i*Aβ and the extent of the **T^0^** threshold are constant, and the lifespan is assumed to terminate at 100 years of age; the only variable is the extent of the **T1** threshold. In *panel **A***, the **T1** threshold is chosen deliberately low, so that the accumulation of AβPP-derived *i*Aβ results in no significant neuronal damage. With the increase of the extent of the **T1** threshold, such damage would inevitably occur at the sub-**T1** levels of AβPP-derived *i*Aβ; to indicate the extent of *i*Aβ accumulation where such damage commences, another threshold, the **T^0^** is introduced in *panel **B*** and it is posited that it is this AβPP-derived *i*Aβ-inflicted neuronal damage, occurring between the thresholds **T^0^** and **T1** which causes AACD (on the more precise definition of the upper AACD boundary, see text). In *panel **C***, the extent of the **T1** threshold increases. With the rate of AβPP-derived *i*Aβ accumulation and the extent of the **T^0^** threshold remaining constant, the AACD Zone increases accordingly, as does the duration and the severity of the dysfunction. While the timing of the commencement of AACD does not change with the increasing extent of the **T1** threshold, the timing of the commencement of the second AD stage increases in a direct proportion, and the probability of developing AD within the remaining lifespan decreases in an inverse proportion to the increase in the extent of the **T1** threshold. In *panel **D***, the extent of the **T1** threshold is such that the level of AβPP-derived *i*Aβ does not reach the **T1** threshold within the lifespan of an individual. With the extent of the **T^0^** threshold and the rate of AβPP-derived *i*Aβ accumulation fixed, the timing of the commencement of AACD remains constant, but the AACD Zone further increases. On the other hand, since the **T1** threshold is not crossed, there is no activation of the AβPP-independent *i*Aβ production pathway, no stage two of AD ensues, no AD occurs. Note, however, that the **T1** threshold would be crossed and AD would certainly occur provided the lifespan is long enough.

The present section analyzes effects of the extent of the **T1** threshold and considers the nature of AACD. The mechanistic definition of AACD has been provided previously in [2]. It is based on the understanding of the nature of an AACD-causing agent. What is the cause of AACD? To answer this question, we would like to invoke again the principal basis for the formulation of the ACH. It was the discovery of a single mutation affecting Aβ and segregating with FAD [7]. This observation was deemed sufficient to deduce, by extrapolation, that Aβ causes FAD. In case of AACD, we have an observation of a similar nature and comparable caliber: a mutation affecting Aβ, in fact a mutation of Aβ, the Icelandic mutation [4,5], protects from AACD as well as from AD. Applying the same logic, one can, by inversion, infer that Aβ causes AACD. The nature of AACD, i.e., in what manner Aβ executes its causative action in AACD, is indicated by the analysis of the effect of the extent of the **T1** threshold on the dynamics of AD (and apparently of AACD) within the same temporal boundaries as those utilized in the preceding section. In this analysis, the rate of accumulation of AβPP-derived Aβ is presumed to be constant, a given. The extent of the **T1** threshold, however, is increasing in succeeding panels of Figure 5. As in the preceding section, this analysis is not concerned with the effects of changes in the rate of *i*Aβ accumulation above the **T1** threshold or in the extent of the **T2** threshold, the parameters discussed in Section 20 below.

In panel **A** of Figure 5, the **T1** threshold is chosen deliberately low, low enough that the accumulation of AβPP-derived *i*Aβ results in no significant cellular damage. It is logically reasonable, however, to assume that with the **T1** threshold of variable extent, this is not always the case. As the extent of **T1** increases, AβPP-derived *i*Aβ is bound to reach the sub-**T1** level, inadequate to trigger the second AD stage but sufficient to cause the consequential neuronal damage. To indicate the extent of *i*Aβ accumulation where such damage commences, another threshold, the **T^0^**, is introduced in panel **B** of Figure 5. We posit that it is this AβPP-derived *i*Aβ-inflicted neuronal damage, occurring between the thresholds **T^0^** and **T1** which causes AACD (when in such cases the **T1** threshold is crossed, AACD morphs, by definition, into AD; see the following section on the subject), that the differential in *i*Aβ levels between the extents of the **T^0^** and **T1** thresholds constitutes the “AACD Zone” (gradient-pink boxes in Figure 5), and that the duration (time period between the **T^0^** and **T1** threshold crossings by AβPP-derived *i*Aβ) and the consequent severity of AACD depend on the size of the AACD Zone, i.e., the **T1**/**T^0^** differential, and the rate of AβPP-derived *i*Aβ accumulation (the lower the rate, the longer it takes to traverse the differential and the longer the duration). 

In panel **C** of Figure 5, the extent of the **T1** threshold increases. With the rate of AβPP-derived *i*Aβ accumulation and the extent of the **T^0^** threshold remaining constant, the AACD Zone increases accordingly, as does the duration and the severity of the dysfunction (it is assumed that higher levels of AβPP-derived *i*Aβ over the **T^0^** threshold equals greater neuronal cells damage). Importantly, while the timing of the commencement of AACD does not change with the increasing extent of the **T1** threshold (it depends solely on the extent of the **T^0^** threshold and the rate of accumulation of AβPP-derived *i*Aβ, both parameters constant in the present narrative), the timing of the commencement of the second AD stage increases in a direct proportion, and the probability of developing AD within the remaining lifespan decreases in an inverse proportion of the increase in the extent of the **T1** threshold. Finally, in panel **D** of Figure 5, the extent of the **T1** threshold is such that the level of AβPP-derived *i*Aβ does not reach it within the lifespan of an individual. With the extent of the **T^0^** threshold and the rate of AβPP-derived *i*Aβ accumulation fixed, the timing of the commencement of AACD remains constant, but the AACD Zone, as well as the duration and the severity of the dysfunction further increase. On the other hand, since the **T1** threshold is not crossed, there is no activation of the AβPP-independent *i*Aβ production pathway, no stage two of AD ensues, no AD occurs (note, however, that the **T1** threshold would be crossed and AD would occur provided the lifespan is long enough). It appears, therefore, that a kind of a trade-off is in play: A decrease in the probability of AD (or its delay and, possibly, avoidance) due to the elevated **T1** threshold is paid for by the increased probability of the occurrence of AACD. Any cognitively functional individual would probably embrace such trade-off. But, potentially, there is no need to choose a lesser of two evils; as described elsewhere [1,2] and discussed below, *a single, once-in-a-lifetime-only administration of a preventive or curative treatment is apparently capable of protecting from both AD and AACD for the remaining lifespan of an individual regardless of the extent of the **T1** threshold.*

To summarize, in light of the above, in the ACH2.0 framework, AACD is defined as the symptomatic manifestation of the neuronal cell damage caused by AβPP-derived *i*Aβ accumulated to the concentration range between the **T^0^** and **T1** thresholds [2], a process that evolves into AD if and when *i*Aβ levels reach and cross the **T1** threshold and activate the AβPP-independent *i*Aβ generation pathway. Its occurrence requires a relatively high extent of the **T1** threshold, certainly in excess of that of the **T^0^** threshold. In this context, AACD can be considered an extended stage One of AD, or, more precisely, an extended segment of the first AD stage; “extended” in terms of the augmented (in comparison with AD that is not preceded by AACD) capacity to accumulate AβPP-derived *i*Aβ prior to the **T1** threshold crossing [2]. Importantly, statistical age of the onset of AACD is greater than that of the onset of SAD [100,101]. This observation is consistent with the notion that in the population that eventually develops AACD, *the **T^0^** threshold is higher than the **T1** threshold in the SAD-predisposed population*, i.e., that *the low extents of the **T1** threshold appear to be a predisposition contributing to the occurrence of SAD*. This notion is of a considerable importance for two reasons. First, drugs targeting the accumulation of AβPP-derived *i*Aβ, which are inapplicable at the second AD stage (symptomatic AD), could be nevertheless effective in the treatment of symptomatic AACD (see below). Second, any preventive treatment effective for AD would be equally effective in prevention of AACD.

## 11. Symptoms of AACD-Associated Cognitive Impairment May Overlap with and Could Be Indistinguishable from Those of AD-Associated Mild Cognitive Impairment

**Figure 6:**
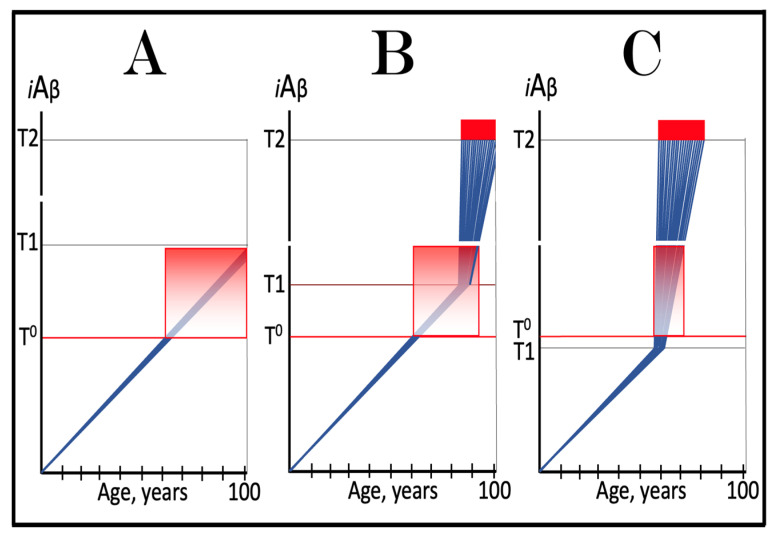
Symptoms of AACD-associated cognitive impairment may overlap with and could be indistinguishable from those of AD-associated mild cognitive impairment. *iA*β: Intraneuronal Aβ levels. ***T^0^***: *i*Aβ levels that trigger neuronal damage manifesting as AACD. ***T1***: *i*Aβ level that triggers elicitation of the integrated stress response, initiation of AβPP-independent generation of *i*Aβ, and the activation of the AD Engine. ***T2***: *i*Aβ level that triggers cell’s commitment to apoptosis. *Red blocks*: Fraction of affected neurons either committed to apoptosis or dead. *Gradient-pink boxes*: “AACD Zone”, the differential between the **T^0^** and **T1** threshold levels of AβPP-derived *i*Aβ. The rate of accumulation of AβPP-derived *i*Aβ and the extent of the **T^0^** threshold are constant, and the lifespan is assumed to terminate at 100 years of age; the only variable is the extent of the **T1** threshold. In panel **A** the **T1** is high and is not reached within the lifespan of an individual. AACD commences with the crossing of the **T^0^** threshold and continues for the remaining portion of the lifespan (gradient-pink box). In this case, *i*Aβ-caused cognitive impairment is clearly attributable to AACD. In panel **B,** the T1 threshold is lowered. The same range of *i*Aβ within the gradient-pink box as shown in panel **A** is divided in panel **B** into two portions: pre-**T1** crossing and post-**T1** crossing. Because the range of *i*Aβ within the gradient-pink boxes in panels **A** and **B** is the same, the symptoms are also the same. But pre-**T1** crossing, they comprise AACD-associated cognitive impairment, whereas post-**T1** crossing, they constitute AD-associated mild cognitive impairment. In panel **C**, the same *i*Aβ range within the gradient-pink box as in panels **A** and **B** occurs entirely post-**T1** crossing. Since the *i*Aβ range within the box is the same as in other panels, the symptoms also are, but now they constitute, in their entirety, AD-associated mild cognitive impairment. Note that since the rate of *i*Aβ accumulation is greater post-**T1** than pre-**T1** crossing, the duration of symptoms decreases in successive panels.

From the definition of AACD as the symptomatic manifestation of the neuronal cell damage caused by *i*Aβ accumulated within a certain concentration range [2], it follows that symptoms of AACD may overlap with and could be indistinguishable from those of AD-associated mild cognitive impairment. Indeed, consider the situation depicted in Figure 6. In panels **A** through **C** of Figure 6, all kinetic parameters are, with one exception, constant. These parameters include the extents of the **T^0^** and **T2** thresholds as well as the rate of *i*Aβ accumulation. The only exception is the extent of the **T1** threshold. In panel **A** of Figure 6 it is high and is not reached within the lifespan of an individual. AACD commences with the crossing of the **T^0^** threshold and continues for the remaining portion of the lifespan (gradient-pink box). In this case, *i*Aβ-caused cognitive impairment is clearly attributable solely to AACD. In panel **B** of Figure 6, the **T1** threshold is lowered. The same range of *i*Aβ within the gradient-pink box as shown in panel **A** is divided in panel **B** into two portions: pre-**T1** crossing and post-**T1** crossing. Because the range of *i*Aβ within gradient-pink boxes is the same in panels **A** and **B**, symptoms are also the same. But pre-**T1** crossing they are AACD-associated cognitive impairment (AACD-CI), whereas post-**T1** crossing they constitute AD-associated mild cognitive impairment (AD-MCI). In panel **C** of Figure 6, the same *i*Aβ range within the gradient-pink box as in panels **A** and **B** occurs entirely post-**T1** crossing. Since the *i*Aβ range within the box is the same as in panels **A** and **B**, the symptoms also are, but now they constitute, in their entirety, AD-associated mild cognitive impairment. Note that, since the rate of *i*Aβ accumulation is greater post-**T1** than pre-**T1** crossing, the duration of symptoms decreases in successive panels of Figure 6.

Conceivably, it could be argued that the above terminological distinctions are purely semantic; after all, the same symptoms are caused by *i*Aβ of any origin within a certain range of concentration, and the symptoms are the symptoms, regardless of what name you attach to them. This, however, is certainly not the case in the situation under discussion: here, the distinction is not semantic but functional because mechanisms underpinning the two conditions (AACD-CI and AD-MCI) are distinctly different. Indeed, due to its underlying mechanism (i.e., the accumulation of AβPP-derived *i*Aβ to a certain range), AACD-CI can be treated by drugs reducing the influx of AβPP-derived *i*Aβ and thus suppressing its rate of accumulation [1,2,3], whereas the same drugs would be completely ineffective in the treatment of AD-MCI, which is driven by *i*Aβ produced independently from AβPP [1,2,3]. On the other hand, drugs reducing *i*Aβ levels via its targeted degradation regardless of its origin (i.e., both AβPP-derived and produced independently of AβPP), such as the enhancers of *i*Aβ-cleaving activities of BACE1 and BACE2, would be equally effective in treatment of both AACD-CI and AD-MCI [1,2,3] (further discussed below). In light of the above, since the symptoms of AACD-CI and AD-MCI could be overlapping, the determination of their origin could be of substantial importance. To determine whether AβPP-derived *i*Aβ levels have crossed the **T1** threshold is, however, challenging. Since the **T1** threshold differs individually, the objective operational criterion to determine it’s crossing by AβPP-derived *i*Aβ is the detection of the activity of AβPP-independent *i*Aβ production pathway. The feasible means to assess the activity of this pathway are addressed below (Section 22 and Section 26).

## 12. Putative Principles of the AD and AACD Dynamics

The line of reasoning pursued in the preceding sections suggests the following putative principles of the AD and AACD dynamics. These principles are concerned with events occurring within the first stage of AD and do not address the processes taking place at the second AD stage, which are discussed in detail below. Moreover, the kinetics of stage Two of AD influences neither that of its stage One nor any of the principles formulated below.

1. At a given extent of the **T1** threshold, the timing of the commencement of the second AD stage (effectively the timing of AD, referred to below as such) is inversely proportional to the rate of accumulation of AβPP-derived *i*Aβ.
2. At a given rate of accumulation of AβPP-derived *i*Aβ, the timing of the commencement of AD is directly proportional to the extent of the **T1** threshold.
3. In the both cases mentioned above, either the rate of AβPP-derived *i*Aβ accumulation or the extent of the **T1** threshold or the combination of both could be such that the timing of the commencement of AD would exceed the lifespan of an individual.
4. Combination of the rate of accumulation of AβPP-derived *i*Aβ and the extent of the **T1** threshold determine the susceptibility of an individual to AD within a typical lifespan.
5. Regardless of the rate of AβPP-derived *i*Aβ accumulation and of the extent of the **T1** threshold, the occurrence of AD is inevitable given sufficient duration of the lifespan.
6. At a given extent of the **T1** threshold and regardless of the rate of AβPP-derived *i*Aβ accumulation, AACD would not occur if the **T1** threshold is sufficiently low (not exceeding the **T^0^** threshold), but AD may occur, subject to the rate of *i*Aβ accumulation.
7. When the extent of the **T1** threshold is sufficiently high, i.e., exceeds that of the **T^0^** threshold, and the extent of the **T^0^** threshold is constant, the timing of the commencement of AACD is inversely proportional to the rate of accumulation of AβPP-derived *i*Aβ.
8. With the extent of the **T^0^** threshold and the rate of AβPP-derived *i*Aβ accumulation fixed, the timing of the commencement of AACD remains constant, but the AACD Zone increases in direct proportion to the increasing extent of the **T1** threshold.
9. At a given rate of accumulation of AβPP-derived *i*Aβ, the duration and severity of AACD are directly proportional to the differential between the extents of the **T^0^** and **T1** thresholds (the “AACD Zone”).
10. Given the extent of the **T^0^** threshold is always lower than that of the **T1** threshold and regardless of the rate of AβPP-derived *i*Aβ accumulation and of the extent of the **T1** threshold, AACD would inevitably occur given sufficient duration of the lifespan.
11. The **T1** threshold is a demarcation line between AD and AACD; when it is crossed by the bulk (or sufficient fraction) of affected neurons, AACD evolves into AD.
12. Given a limited lifespan and sufficient AACD Zone, AACD may develop without AβPP-derived *i*Aβ reaching the **T1** threshold.
13. Given a limited lifespan, AACD is not always followed by AD (but AD will always follow if the lifespan is long enough).
14. Given the sufficiently high **T1** threshold, if AD occurs, it is always preceded by AACD.

## 13. Potential Fluidity of Kinetic Parameters Defining the Dynamics and Occurrence of AD and AACD

The preceding sections depicted the kinetic parameters that define the occurrence of AD and AACD as constant throughout the lifetime. This, however, is not necessarily the case. In principle, the incidence of both conditions, AD and AACD, is determined primarily by the following three parameters: the extents of the **T^0^** and **T1** thresholds and the rate of accumulation of AβPP-derived *i*Aβ. The crossing of the **T^0^** threshold by AβPP-derived *i*Aβ activates AACD; that of the **T1** threshold triggers AD. Each of these parameters is potentially variable and can modulate with age in both linear and non-linear manner. Since the crossing of the **T^0^** and **T1** thresholds triggers the commencement of AACD and of AD respectively, it can be assumed that increases in the extents of the **T^0^** and **T1** thresholds in a time-dependent manner during the lifetime would delay or prevent the occurrence of AACD and AD and the lowering of these thresholds would accelerate the commencement of both conditions. Similarly, the time-dependent reduction in the rate of accumulation of AβPP-derived *i*Aβ would delay or prevent the incidence of both conditions, whereas its increase as a function of time would accelerate the commencement of both AACD and AD. In any case it should be emphasized that, importantly, even with the time-dependent modulation of the extents of the relevant thresholds or the potential aging-related variability of the rate of accumulation of AβPP-derived *i*Aβ, the dynamic aspects of the **T^0^** and **T1** crossings would remain subjects to the same logic as applied above and would be fully consistent with the proposed therapeutic strategies for AD and AACD discussed below.

## 14. Protection from AD and AACD Conferred by the Icelandic AβPP Mutation Is Due to Dynamic Changes in *i*Aβ Accumulation: Mechanistic Interpretation in the ACH2.0 Perspective 

The validity of any novel concept demands that it is consistent with all prior observations and is capable of explaining all outstanding unexplained phenomena and making meaningful predictions. The ACH2.0 does all of this. It explains, as discussed above, why numerous candidate AD drugs showed no efficacy in human clinical trials (due to the occurrence of AβPP-independent production of *i*Aβ) yet were very effective in animal studies (due to the lack of the second AD stage, i.e., the absence of the generation of *i*Aβ independently of AβPP in animal models). It predicts that at least some of those drugs could be effective in prevention of AD and in the treatment of AACD but that conceptually different types of drugs are required for the treatment of symptomatic AD [1,2,3]. It also predicts the occurrence in human AD-affected neurons of C99 and Ab species that contain additional methionine residue at their N-terminus (discussed below). The present and several following sections consider the phenomena of protection from AD and AACD and of causation of the early onset of AD (FAD) and provide the mechanistic interpretation of both occurrences in terms of the ACH2.0 and, more specifically, in terms of the dynamics of accumulation of AβPP-derived *i*Aβ.

The Icelandic AβPP mutation A673T, also known as Aβ mutation A3T since it occurs *within* Aβ, was shown to protect its carriers from AD by augmenting the efficiency of BACE1-mediated cleavage at the β’ site within Aβ [1,2]. Remarkably, it also protects from the pervasive aging-associated cognitive decline, AACD [1,2]. This is a striking observation. It implies that Aβ is involved in AACD, and that its role in this dysfunction is apparently similar, i.e., causative, to the role it performs in AD. Considering the dynamics of *i*Aβ accumulation within the ACH2.0 framework, described above, it is clear how the Icelandic mutation protects from AD. It does so by increasing the rate of BACE1 cleavage at the β′ site within Aβ segment of the precursor molecule as well as within already formed *i*Aβ, thus lowering the rate of accumulation of AβPP-derived *i*Aβ. Indeed, in the mutation carriers less Aβ is produced in the AβPP proteolytic pathway and more *i*Aβ is cleaved at the β′ site, therefore the steady state influx of *i*Aβ is lowered and the rate of its accumulation is reduced. Consequently, AβPP-derived *i*Aβ levels do not reach the **T1** threshold within the lifespan of a mutation carrier (or reach it much later than in wild-type AβPP carriers), and the disease either does not occur or is delayed. As for AACD, its definition as the extended segment of the first AD stage, commencing with the crossing of the **T^0^** threshold by AβPP-derived *i*Aβ, provides the explanation for how the Icelandic mutation protects from aging-associated cognitive decline: in exactly the same manner that it renders protection from AD, namely by lowering the rate of AβPP-derived *i*Aβ accumulation with the result that *i*Aβ levels do not reach the **T^0^** threshold within the lifetime of a mutation carrier (or reach it at a substantially later age than in wild-type AβPP carriers). 

**Figure 7:**
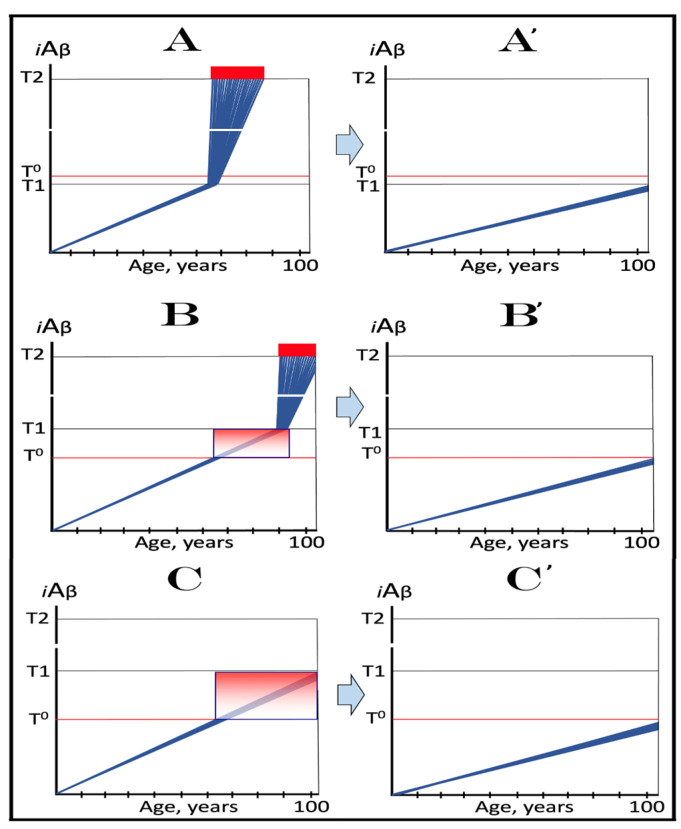
Protective effect of the Icelandic mutation for AD and AACD: Mechanistic interpretation in the ACH2.0 perspective. *iA*β: Intraneuronal Aβ levels. ***T^0^***: *i*Aβ levels that trigger neuronal damage manifesting as AACD. ***T1***: *i*Aβ level that triggers elicitation of the integrated stress response, initiation of AβPP-independent generation of *i*Aβ, and the activation of the AD Engine. ***T2***: *i*Aβ level that triggers cell’s commitment to the apoptotic pathway. *Red blocks*: Fraction of affected neurons either committed to apoptosis or dead. *Gradient-pink boxes*: “AACD Zone”, the differential between the **T^0^** and **T1** threshold levels of AβPP-derived *i*Aβ (more precisely, between the **T^0^** and the maximum level reached by AβPP-derived *i*Aβ short of the T1 threshold). *Panels **A**, **B**, and **C*** depict three principal variants of the *i*Aβ-caused disease occurring in wild-type AβPP carriers. The rate of AβPP-derived *i*Aβ accumulation is assumed constant and so is the extent of the **T^0^** threshold; the lifespan in each case is limited to 100 years of age. On the other hand, the extent of the **T1** threshold is variable and dictates whether AACD and AD do or do not occur. In *panel **A***, the **T1** threshold is below the AβPP-derived *i*Aβ level (**T^0^** threshold) required for the initiation of AACD. When the **T1** threshold is reached, the AβPP-independent *i*Aβ generation pathway is activated and AD commences. In *panel **B***, the **T^0^** threshold level is below that of the T1 threshold. When the levels of AβPP-derived *i*Aβ reach the former, AACD commences and persists until AβPP-derived *i*Aβ crosses the latter, i.e., for the duration of the AACD Zone (gradient-pink box), whereupon it evolves into AD. In *panel **C***, the extent of the **T1** threshold is such that at a given rate of accumulation of AβPP-derived *i*Aβ, the **T1** threshold cannot be reached, the AβPP-independent *i*Aβ generation pathway cannot be activated, and AD cannot occur within the lifetime of an individual. When AβPP-derived *i*Aβ levels cross the **T^0^** threshold, AACD commences and continues for the remaining part of the lifespan. *Panels **A’**, **B’**, and **C’*** depict mechanistic interpretation of the protective effect of the Icelandic AβPP mutation within the framework of the ACH2.0. In all three variants of potential AD/AACD, the rate of accumulation of AβPP-derived *i*Aβ is lowered. In *panel **A’***, it is such that levels of AβPP-derived *i*Aβ do not reach the T1 threshold within the lifespan of an individual. In *panels **B’** and **C’,*** the rate of accumulation of AβPP-derived iAβ is rendered such that its levels do not reach the **T^0^** (and **T1**) threshold within the individual’s lifetime. Accordingly, in all three variants, neither AACD nor AD occurs within the lifespan of the Icelandic mutation carriers (or occurs substantially later than in wild-type AβPP carriers).

The above mechanistic interpretation of the protective effect of the Icelandic mutation in AD and AACD is illustrated in Figure 7. Panels **A**, **B**, and **C** of Figure 7 depict diagrammatically three principal variants of the *i*Aβ-caused diseases, i.e., AD and AACD, occurring in wild-type AβPP carriers. In these panels, the rate of AβPP-derived *i*Aβ accumulation is constant, a given, and so is the extent of the **T^0^** threshold; the lifespan in each case is assumed to end at 100 years of age. On the other hand, the extent of the **T1** threshold is variable and dictates whether AACD and AD do or do not occur. In panel **A** of Figure 7, the **T1** threshold is below the AβPP-derived *i*Aβ level required for the initiation of AACD (**T^0^** threshold). When the **T1** threshold is reached, the AβPP-independent *i*Aβ generation pathway is activated and AD commences. When *i*Aβ (mostly produced at this point independently of AβPP) levels reach the **T^0^** threshold, AD-MCI symptoms would occur, as discussed above, and morph rapidly into AD. 

Panel **B** of Figure 7 depicts a scenario where the **T^0^** threshold level is below that of the **T1** threshold. When the levels of AβPP-derived *i*Aβ reach the former, AACD commences and persists until AβPP-derived *i*Aβ crosses the latter, i.e., for the duration of the AACD Zone (shown as gradient-pink boxes in Figure 7), whereupon it evolves into AD. In panel **C** of Figure 7, the extent of the **T1** threshold is such that at a given rate of accumulation of AβPP-derived *i*Aβ, the **T1** threshold cannot be reached, the AβPP-independent *i*Aβ generation pathway cannot be activated and AD cannot occur within the lifetime of an individual. When AβPP-derived *i*Aβ levels cross the **T^0^** threshold, AACD commences and continues (increasing in severity with elevating levels of *i*Aβ) for the remaining part of the lifespan.

Panels **A**’, **B**’, and **C**’ of Figure 7 depict mechanistic interpretation of the protective effect of the Icelandic mutation within the framework of the ACH2.0. In all three variants of potential AD/AACD, the rate of accumulation of AβPP-derived *i*Aβ is lowered. In panel **A**’ of Figure 7, it is such that neither the levels of AβPP-derived *i*Aβ do reach the **T1** threshold within the lifespan of an individual nor AD occurs. In panels **B**’ and **C**’ of Figure 7, the rate of accumulation of AβPP-derived iAβ is rendered such that neither levels of AβPP-derived *i*Aβ reach the **T^0^** (and, of course, **T1**) threshold within the individual’s lifetime, nor AACD (and, of course, AD) ensues. Thus, in all three variants discussed above, neither AACD nor AD occur within the lifespan of the Icelandic mutation carriers (or occur substantially later than in wild type AβPP carriers). 

## 15. Confirmation of the Concept: Effect of the Flemish Aβ Mutation as the Ultimate Empirical Test in Nature-Conducted Experiment

Conceptually, the notion that the persistent suppression of the rate of *i*Aβ accumulation protects from AD could be assessed empirically. An experiment can be envisioned where a physiologically occurring cleavage within Aβ (which indeed takes place as discussed below) is suppressed, a scenario which is diametrically opposite to that happening in Icelandic mutation carriers. The suppression of Aβ cleavage would result in its increased production, in the elevated steady-state influx of *i*Aβ, and, consequently, in the augmented rate of *i*Aβ accumulation, the accelerated crossing of the **T1** threshold, and the early activation of the AβPP-independent *i*Aβ production and of the second AD stage. Thus, if the concept, linking the rate of *i*Aβ accumulation to the occurrence of AD, were correct, early-onset AD should result. *Just this experiment was, in fact, carried out by nature in the form of the Flemish A692G AβPP mutation*. It is also known as the A21G Aβ Aβ mutation because it affects the residue 21 of Aβ, which contiguously follows cleaving sites of BACE2 at residues 19 and 20 within Aβ. A21G substitution suppresses the physiologically operating cleaving activity of BACE2 within Aβ segment of the precursor molecule as well as within already formed *i*Aβ. This increases the production of Aβ in the AβPP proteolytic pathway and decreases cleavages of already formed *i*Aβ, thus elevating the steady-state influx of *i*Aβ and augmenting the rate of *i*Aβ accumulation (further discussed in the following section). The result is the early-onset FAD that manifests symptomatically at the mid-forties [62,102,103]. *The outcome of this “natural” experiment, therefore, constitutes a confirmation of the concept connecting the rate of iAβ accumulation and the occurrence of AD.*

## 16. Dynamics of the Early Onset of FAD: Mechanistic Interpretation in the ACH2.0 Perspective

### 16.1. Category One of FAD: Mutations Causing the Elevation in the Rate of Accumulation of AβPP-Derived iAβ 

The Flemish FAD mutation is one of many causing the early onset of the disease. In the ACH2.0 perspective, the modus operandi of every such mutation is essentially the same: Acceleration of the crossing of the **T1** threshold by AβPP-derived *i*Aβ. As for how this is achieved, FAD mutations can be separated into two categories. One category is exemplified by Flemish [62] and Swedish [59] AβPP mutations, as well as by presenilins mutations that facilitate gamma-cleavage of C99 on the internal membranes, thus increasing the intraneuronal retention of AβPP-derived Aβ [60]. In these cases, the core causative event is the increase in the steady-state influx of AβPP-derived *i*Aβ and consequent elevation of the rate of its accumulation. In Flemish FAD mutation case, this is due to suppression of physiologically operating activity of BACE2 that cleaves Aβ segment within AβPP and the C99 fragment as well as within *i*Aβ. In case of the Swedish FAD [59] and certain PSEN [60] mutants, the increase in the steady-state influx of *i*Aβ is due to the augmented gamma-cleavage of the C99 fragment on the intracellular membranes rather than on the plasma membrane, which results in the increased intraneuronal retention of AβPP-derived *i*Aβ. Consequently, in either case, the rate of AβPP-derived *i*Aβ accumulation increases in comparison with wild-type AβPP carriers. 

**Figure 8:**
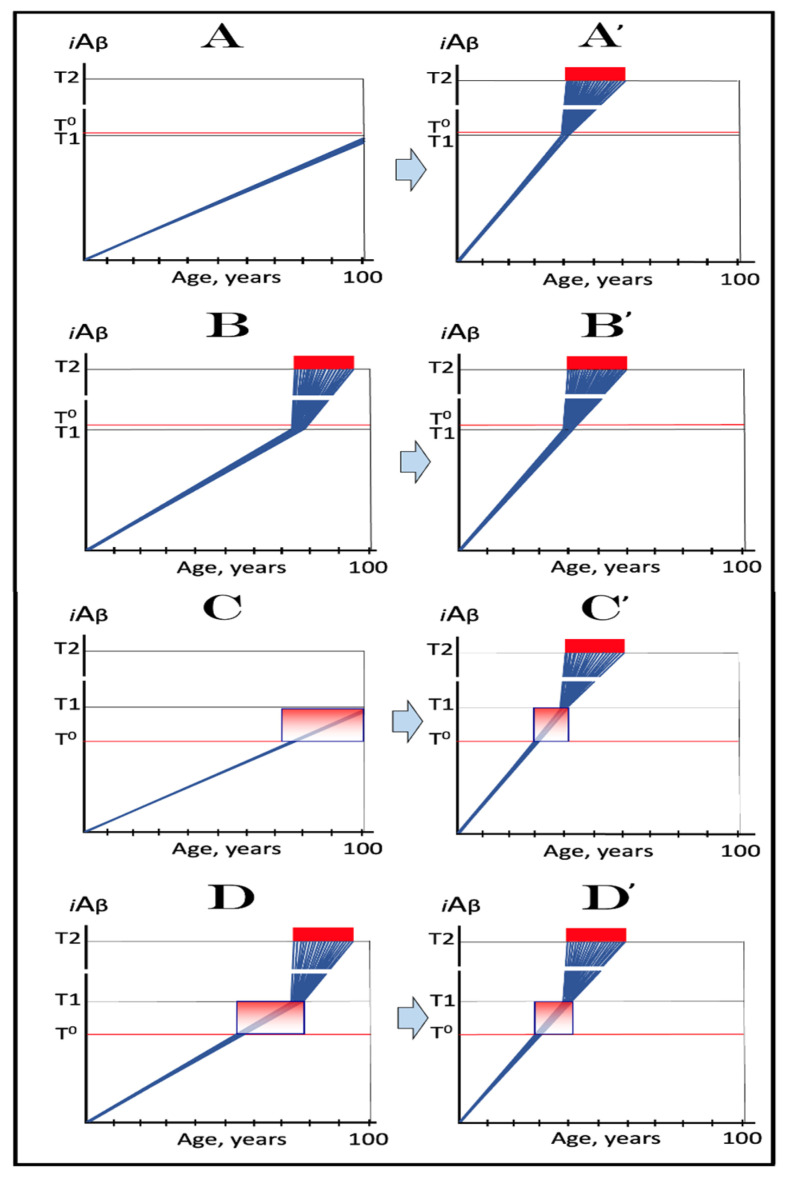
Early onset of AD in carriers of category One FAD mutations: Mechanistic interpretation in the ACH2.0 perspective. *iA*β: Intraneuronal Aβ levels. ***T^0^***: *i*Aβ levels that trigger neuronal damage manifesting as AACD. ***T1***: *i*Aβ level that triggers elicitation of the integrated stress response, initiation of AβPP-independent generation of *i*Aβ, and the activation of the AD Engine. ***T2***: *i*Aβ level that triggers cell’s commitment to the apoptotic pathway. *Red blocks*: Fraction of affected neurons either committed to apoptosis or dead. *Gradient-pink boxes*: “AACD Zone”, the differential between the **T^0^** and **T1** threshold levels of AβPP-derived *i*Aβ (more precisely, between the **T^0^** and the maximum level reached by AβPP-derived *i*Aβ short of the **T1** threshold). Assumed lifespan: 100 years. *Panels **A**, **B**, **C**, **D***: Kinetics of *i*Aβ accumulation and progression of disease in wild-type AβPP carriers. Panels **A** and **B**: The extent of the **T^0^** threshold exceed that of the **T1**; no AACD occurs. Panel **A**: The **T1** threshold is not crossed; no AD occurs. Panel **B**: The **T1** threshold is reached and crossed; the late onset AD ensues. Panels **C** and **D**: The **T^0^** threshold is lower than the **T1**. Panel **C**: The **T1** threshold is not crossed; AACD commences upon crossing of the **T^0^** threshold and continues for the remaining lifespan. Panel **D**: Both the **T^0^** and **T1** thresholds are crossed; AACD commences upon the crossing of the former and evolves into late onset AD when the latter is reached. *Panels **A’**, **B’**, **C’**, **D’***: Dynamics of *i*Aβ accumulation and progression of disease in carriers of category One FAD mutations. The steady-state influx of AβPP-derived *i*Aβ is increased and its rate of accumulation augmented. Consequently, the **T1** threshold is reached earlier, AβPP-independent production of *i*Aβ is activated sooner, and the early-onset AD ensues. Panels **A’** and **B’**: The **T^0^** levels exceed those of **T1**; no AACD occurs. Panels **C’** and **D’**: The extent of the **T^0^** threshold is lower than that of the **T1** threshold, and the early-onset AD is preceded by the AACD phase. Due to the steepness of AβPP-derived *i*Aβ accumulation, the duration of the AACD phase is much shorter in mutants than in wild-type AβPP carriers; it rapidly evolves (upon crossing of the **T1** threshold by AβPP-derived *i*Aβ) into early onset AD and therefore could be hard to diagnose as a separate condition. Note that the only dynamic alteration caused by category One FAD mutations is the augmentation of the rate of accumulation of AβPP-derived *i*Aβ.

In wild-type AβPP carriers, two possible scenarios can play out. In the first scenario, which occurs in the majority of the population, the **T1** threshold is not crossed within the lifespan of an individual, and no AD occurs (Figure 8, panel **A**). In the other scenario (in both scenarios the extent of the **T1** is assumed to be lower than that of **T^0^** threshold), *i*Aβ levels reach and cross the **T1** threshold and late-onset AD ensues (Figure 8, panel **B**). In carriers of the first category of FAD mutations with the extent of the **T1** lower than that of **T^0^** threshold, AβPP-derived *i*Aβ accumulates faster, the **T1** threshold is reached sooner, and the early onset of AD results (Figure 8, panels **A**’ and **B**’). 

In wild-type AβPP cases where the extent of the **T^0^** threshold is lower than that of the **T1**, there are also several possibilities. If the **T^0^** and **T1** thresholds are not crossed, neither AACD nor AD occurs (not shown). If the **T^0^** threshold is crossed but the **T1** is not, AACD would commence and continue for the remaining part of the lifespan (Figure 8, panel **C**). If both, the **T^0^** and **T1** thresholds were crossed, AACD would be followed by the late onset AD (Figure 8, panel **D**). In carriers of the first category of FAD mutations with the **T^0^** threshold is lower than the **T1**, due to the augmented accumulation of AβPP-derived *i*Aβ the early onset AD would occur and would be preceded by the AACD stage (Figure 8, panels **C**’ and **D**’). However, due to the steep rate of *i*Aβ accumulation in FAD cases, the AACD stage would be of substantially shorter duration in FAD mutation carriers (Figure 8, panels **C**’ and **D**’) than in their wild-type AβPP counterparts (Figure 8, panels **C** and **D**). In the former, unlike in the latter, AACD would rapidly evolve into AD upon crossing of the **T1** threshold, and could be hard to diagnose as a separate condition.

### 16.2. Category Two of FAD: Mutations That Both Accelerate the Rate of AβPP-Derived iAβ Accumulation and Lower the **T1** Threshold

Another category of FAD mutations includes those, both in AβPP and in presenilins, which cause the increased production of Aβ_42_ [36]. Two factors are at play in the attainment of the **T1** threshold by AβPP-derived *i*Aβ in this category. (a) The first factor is the accelerated cellular uptake of secreted soluble Aβ_42_, which was shown to oligomerize and be taken up by cells twice more efficiently than Aβ_40_ [35]. This results in the accelerated (in comparison with the wild-type) steady-state influx of *i*Aβ, which, in turn, leads to the augmented rate of *i*Aβ accumulation. (b) The second factor is the reduction of the extent of the **T1** threshold due to the documented increased (in comparison with other Aβ species) toxicity of intracellular Aβ_42_. The increased toxicity translates into increased cellular stress and in the reduction in the levels of *i*Aβ_42_ required for elicitation of the ISR, i.e., in the lowering of the **T1** threshold (the **T^0^** threshold is, probably, also lowered). Cumulatively, these two factors substantially accelerate, in comparison with wild-type AβPP carriers, the crossing of the **T1** threshold by AβPP-derived *i*Aβ and the consequent commencement of the second AD stage in carriers of the second category of FAD mutations. 

**Figure 9:**
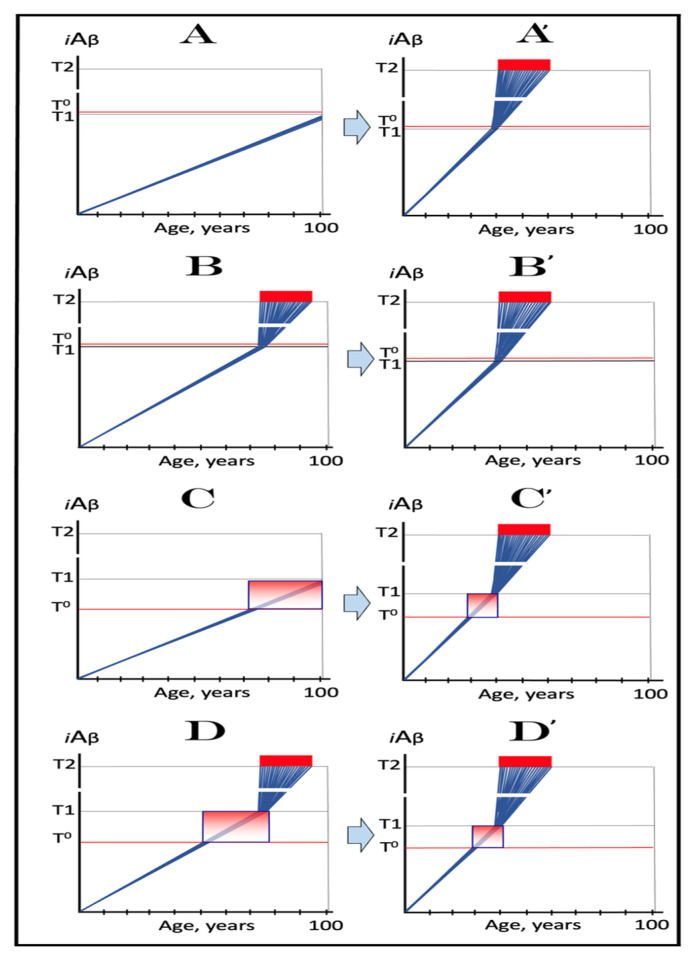
Early onset of AD in carriers of category Two FAD mutations: Mechanistic interpretation in the ACH2.0 perspective. *iA*β: Intraneuronal Aβ levels. ***T^0^***: *i*Aβ levels that trigger neuronal damage manifesting as AACD. *T1*: *i*Aβ level that triggers elicitation of the integrated stress response, initiation of AβPP-independent generation of *i*Aβ and the activation of the AD Engine. ***T2***: *i*Aβ level that triggers cell’s commitment to the apoptotic pathway. *Red blocks*: Fraction of affected neurons either committed to apoptosis or dead. *Gradient-pink boxes*: “AACD Zone”, the differential between the **T^0^** and **T1** threshold levels of AβPP-derived *i*Aβ (more precisely, between the **T^0^** and the maximum level reached by AβPP-derived *i*Aβ short of the **T1** threshold). Assumed lifespan: 100 years. *Panels **A**, **B**, **C**, **D***: Kinetics of *i*Aβ accumulation and progression of disease in wild-type AβPP carriers. Panels **A** and **B**: The **T^0^** levels exceed those of T1; no AACD occurs. Panel **A**: The **T1** threshold is not crossed; no AD occurs. Panel **B**: The **T1** threshold is reached and crossed; the late onset AD ensues. Panels **C** and **D**: The **T^0^** threshold is lower than **T1**. Panel **C**: The **T1** threshold is not crossed; AACD commences upon crossing of the **T^0^** threshold and continues for the remaining lifespan. Panel **D**: Both the **T^0^** and **T1** thresholds are crossed; AACD commences upon crossing of the former and evolves into late onset AD when the latter is reached. *Panels **A’**, **B’**, **C’**, **D’***: Dynamics of *i*Aβ accumulation and progression of disease in carriers of category two FAD mutations, which cause not only the augmentation of steady-state influx of AβPP-derived *i*Aβ and the increase in its rate of accumulation but also the reduction in the extent of the **T1** threshold. The **T1** threshold is reached earlier, AβPP-independent production of *i*Aβ is activated sooner, and the early-onset AD ensues. Panels **A’** and **B’**: The **T^0^** levels exceed those of T1; no AACD occurs. Panels **C’** and **D’**: The extent of the **T^0^** threshold is lower than that of the **T1** threshold, and the early-onset AD is preceded by the AACD phase. Due to the steepness of AβPP-derived *i*Aβ accumulation, the duration of the AACD phase is much shorter than in wild-type AβPP carriers; it rapidly evolves (upon crossing of the **T1** threshold by AβPP-derived *i*Aβ) into early onset AD and therefore could be hard to diagnose as a separate condition. Note that dynamic changes caused by category two FAD mutations are not only the increase in the rate of accumulation of AβPP-derived *i*Aβ but also the reduction in the extent of the **T1** and, probably, **T^0^** thresholds.

This brings about the early onset of AD as shown in Figure 9. The scenarios playing out in wild-type AβPP carriers are similar to those described in the preceding section: In cases where the **T^0^** exceeds the **T1** threshold, no crossing of the **T1** threshold within an individual’s lifetime and no AD (Figure 9, panel **A**) or the crossing of the **T1** threshold, followed by the late onset AD (Figure 9, panel **B**). In carriers of the second category of FAD mutations, both the rate of accumulation of AβPP-derived *i*Aβ is augmented and the T1 threshold is lowered. Consequently in cases with the extent of **T1** lower than that of **T^0^**, the **T1** threshold is reached and crossed substantially sooner and the early onset of AD ensues (Figure 9, panels **A**’ and **B**’). 

In wild-type AβPP cases where the extent of the **T^0^** threshold is lower than that of the **T1**, the possible scenarios are also similar to those discussed in the preceding section, namely (a) no **T^0^** and **T1** threshold crossing and, consequently, no AD and AACD (not shown); (b) crossing of the **T^0^** but not of the **T1** threshold, resulting in in AACD continuing for the remaining lifespan and no AD (Figure 9, panel **C**); and (c) crossing of both the **T^0^** and **T1** thresholds leading to AACD that would be followed by the late onset AD (Figure 9, panel **D**). 

In carriers of the second category of FAD mutations, in cases where the **T^0^** threshold is lower than the **T1**, both the **T^0^** and **T1** thresholds are crossed, and the early onset AD is preceded by the AACD stage (Figure 9, panels **C**’ and **D**’). Due to the steep rate of *i*Aβ accumulation, however, the AACD phase in mutation carriers would be of substantially shorter duration than in wild-type counterparts; would relatively rapidly morph, upon crossing of the **T1** threshold by AβPP-derived *i*Aβ, into AD; and would, possibly, be unnoticeable or hard to diagnose as a separate condition (Figure 9, panels **D/D’**). Note that in panels **A’**, **B’**, **C’** and **D**’, not only is the rate of *i*Aβ accumulation augmented, but the extent of the **T1** threshold is also lowered.

## 17. Protective Icelandic AβPP Mutation as the Ultimate Guide for AD and AACD Therapy

Consider the following: Not only do multiple mutations affecting, apparently solely, Aβ cause AD, but also the Icelandic mutation *within* Aβ protects from both AD and AACD. This is the ultimate argument for the causative role of Aβ in AD and AACD, and a persuasive and defining guide for therapeutic strategies for both conditions. How to prevent and treat AD and AACD according to this guide? The answer is straightforward: Emulate what the Icelandic mutation does. Then, what exactly does the Icelandic mutation do? It simply augments the efficiency of BACE1 cleavage at the β′ site within *i*Aβ and thus reduces the steady-state influx of AβPP-derived *i*Aβ and lowers the rate of its accumulation; ultimately, it delays or prevents (within limits of the lifespan) the crossing of the **T^0^** and/or **T1** thresholds and, consequently, the occurrence of AD and AACD. How to imitate this? This can be accomplished in two ways.

### 17.1. Prevention of AD and AACD by Simulation of the Mode of Operation of the Icelandic AβPP Mutation 

One way is literally. The Icelandic mutation persistently, from birth, reduces the steady-state influx of AβPP-derived *i*Aβ. To imitate this literally, drugs, possibly the existing ones, can be used to accomplish the same. BACE1 inhibitors appear capable of accomplishing this by suppressing the overall AβPP-based production of Aβ; so do, albeit to a lesser extent, antibodies targeting extracellular Aβ (e.g., lecanemab and donanemab [3]) by suppressing its cellular uptake, i.e., its conversion to *i*Aβ. This would reduce the steady-state influx of AβPP-derived *i*Aβ, lower the rate of its accumulation, and delay or prevent the crossing of the **T1** and **T^0^** thresholds and the occurrence of AD and AACD. To literally imitate the effect of the Icelandic mutation, such drugs would have to be administered unremittingly for the entire life, starting early. On the other hand, the outcomes of clinical trials of lecanemab and donanemab (analyzed in [3]) indicate that the type of drug, which targets the influx of AβPP-derived *i*Aβ, can be employed preventively relatively late in life, provided that their administration commences prior to the **T^0^** and **T1** crossings and that their effect is sufficiently potent to preclude further accumulation of AβPP-derived *i*Aβ for the duration of the treatment [3].

**Figure 10:**
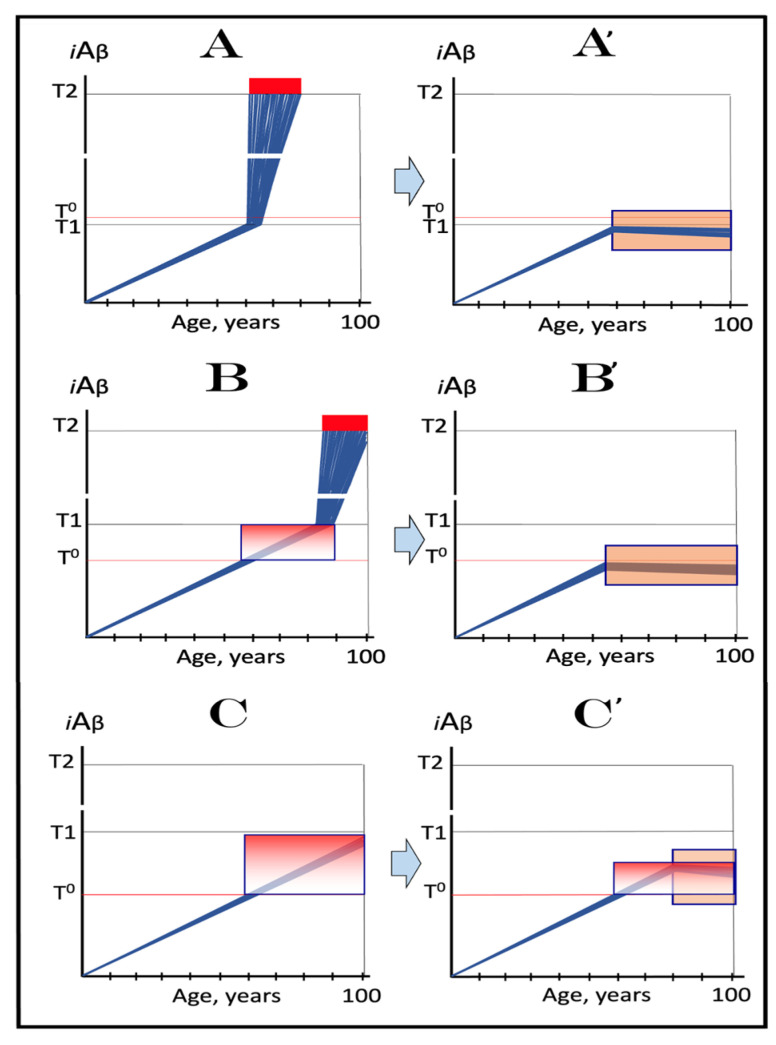
The Icelandic AβPP mutation as the ultimate guide for AD and AACD therapy: Effect of the imitation of the mode of mutation’s operation. *iA*β: Intraneuronal Aβ levels. ***T^0^***: *i*Aβ levels that trigger neuronal damage manifesting as AACD. ***T1***: *i*Aβ level that triggers elicitation of the integrated stress response, initiation of AβPP-independent generation of *i*Aβ and the activation of the AD Engine. ***T2***: *i*Aβ level that triggers cell’s commitment to the apoptotic pathway. *Red blocks*: Fraction of affected neurons either committed to apoptosis or dead. *Gradient-pink Boxes*: “AACD Zone”, the differential between the **T^0^** and **T1** threshold levels of AβPP-derived *i*Aβ (more precisely, between the **T^0^** and the maximum level reached by AβPP-derived *i*Aβ short of the **T1** threshold). *Orange boxes:* Duration of treatment’s administration. The lifespan is assumed to terminate at 100 years of age. *Panels **A***, ***B****,* ***C****:* Dynamics of *i*Aβ accumulation in AD-affected neurons and the progression of the disease in the wild-type AβPP carriers in the absence of treatment. In panel ***A***, the **T^0^** levels exceed those of **T1** and no AACD occurs. In panels ***B*** and ***C***, the **T^0^** threshold is lower than **T1**. AACD commences upon crossing of the **T^0^** threshold and evolves into AD when the **T1** threshold is crossed (panel **B**) or continues for the remaining lifespan if the **T1** threshold is not crossed (panel **C**). *Panels **A’***, ***B’***: Dynamics of AβPP-derived *i*Aβ accumulation under a drug that suppresses its steady-state influx and precludes its further accumulation. The crossing of the **T1** and **T^0^** thresholds is prevented and no disease ensues for the duration of the treatment. *Panel **C’***: The same drug is administered *after* the **T^0^** crossing. It precludes further accumulation of AβPP-derived *i*Aβ and stops or slows the progression of AACD for the duration of the treatment. *Thus, a drug, which suppresses the accumulation of AβPP-derived iAβ, can be only preventive for AD but may constitute a valid treatment for AACD*.

The potential outcomes of treatment with such drugs, initiated late in life, are illustrated in Figure 10. *Panels* **A, B**, and **C** depict the accumulation of *i*Aβ and progression of disease in untreated patients with different relative extents of the **T^0^** and **T1** thresholds in three principal variants of AD/AACD discussed above (see for comparison Figure 7 above). In panel **A** of Figure 10, the **T1** threshold is lower than **T^0^** and its crossing triggers AD. In panel A’, a drug is administered, prior to the **T1** crossing, that precludes further accumulation of AβPP-derived *i*Aβ and prevents AD for the duration of the treatment (orange box). In panel **B**, the **T^0^** is lower than **T1** and AD is preceded by AACD. In panel **B**’, a drug is administered prior to the **T^0^** crossing. It stops further accumulation of AβPP-derived *i*Aβ and prevents both AACD and AD for the duration of the treatment. In panel **C,** the **T1** threshold is not crossed and the **T^0^** crossing triggers AACD that continues for the remaining lifespan of an individual. In panel **C**’, a drug is administered *after* the **T^0^** crossing. It precludes further accumulation of AβPP-derived *i*Aβ and stops or slows the progression of AACD for the duration of the treatment. *Thus a drug, which suppresses the accumulation of AβPP-derived iAβ, can be only preventive for AD but may constitute a valid treatment for AACD*. This is because, whereas AACD is caused by AβPP-derived *i*Aβ, AD is driven by *i*Aβ produced independently of AβPP and is, therefore, insensitive to drugs targeting AβPP-derived *i*Aβ [1,2].

### 17.2. The Mode of Operation of the Protective Icelandic AβPP Mutation Is Physiologically Constrained and Can Be Substantially Improved Upon: Transient, Short-Duration iAβ Depletion Therapy for AD and AACD

Another way to emulate the Icelandic mutation is to follow the essential logic (i.e., the spirit rather than the letter) of its operation but do one better. We can achieve the same outcome, namely the extension of the duration of time required for AβPP-derived *i*Aβ to reach the **T1** and/or **T^0^** thresholds, in a stepwise manner by transiently depleting the levels of *i*Aβ before they cross the **T^0^** and **T1** thresholds, thus collapsing its population and forcing the resumption of its accumulation from a lower baseline, an objective that can be accomplished by just a few, possibly a single, strategically timed transient *i*Aβ depletion treatments. 

**Figure 11:**
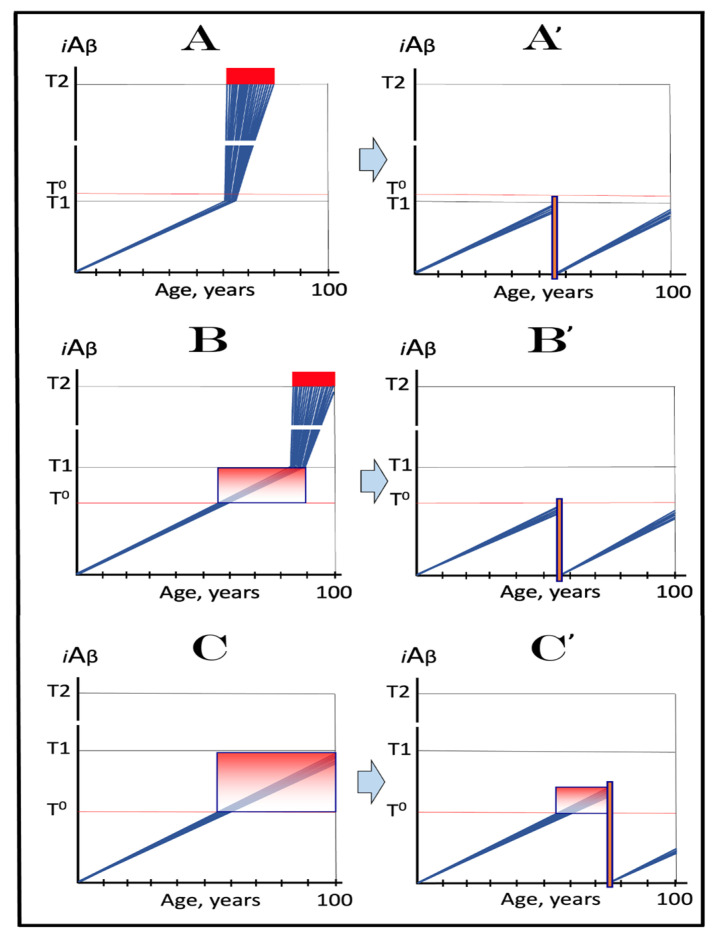
Protective action of the Icelandic AβPP mutation can be improved upon: effect of the transient depletion of *i*Aβ. *iA*β: Intraneuronal Aβ levels. ***T^0^***: *i*Aβ levels that trigger neuronal damage manifesting as AACD. ***T1***: *i*Aβ level that triggers elicitation of the integrated stress response, initiation of AβPP-independent generation of *i*Aβ and the activation of the AD Engine. ***T2***: *i*Aβ level that triggers cell’s commitment to the apoptotic pathway. *Red blocks*: Fraction of affected neurons either committed to apoptosis or dead. *Gradient-pink boxes*: “AACD Zone”, the differential between the **T^0^** and **T1** threshold levels of AβPP-derived *i*Aβ (more precisely, between the **T^0^** and the maximum level reached by AβPP-derived *i*Aβ short of the **T1** threshold). *Orange boxes:* Duration of treatment’s administration. The lifespan is assumed to terminate at 100 years of age. *Panels **A***, ***B****,* ***C****:* Dynamics of *i*Aβ accumulation in AD-affected neurons and the progression of the disease in the wild-type AβPP carriers in the absence of treatment. In panel ***A***, the **T^0^** levels exceed those of **T1** and no AACD occurs. In panels ***B*** and ***C***, the **T^0^** threshold is lower than **T1**. AACD commences upon crossing of the **T^0^** threshold and evolves into AD when the **T1** threshold is crossed (panel **B**) or continues for the remaining lifespan if the **T1** threshold is not crossed (panel **C**). *Panels **A’***, ***B’***: Dynamics of AβPP-derived *i*Aβ accumulation following a transient *i*Aβ depletion treatment administered prior to the crossing of the T1 and T^0^ thresholds. The *i*Aβ population is collapsed and its accumulation is resumed from a low baseline. The duration of the treatment is defined by the desired extent of *i*Aβ depletion and could be as short as few days, akin to an antibiotic treatment’s regimen. As shown, *i*Aβ is completely (or nearly completely) depleted and its build-up to the **T1** and **T^0^** levels would exceed an individual’s lifespan; no disease would occur. *Panel **C’***: A transient *i*Aβ depletion treatment is applied to AACD patient after the **T^0^** crossing. *Following the depletion, iAβ levels are well below the **T^0^** threshold and the patient is technically cured of AACD* (subject to complete recovery of the affected neurons following the *i*Aβ depletion treatment). As shown, de novo accumulating AβPP-derived *i*Aβ does not reach the **T^0^** threshold and AACD does not recur within the remaining lifetime of the treated patient. Note that whereas complete or nearly complete *i*Aβ depletion is shown in panels **A’**–**C’**, any reduction in its baseline would be therapeutically beneficial in proportion to the extent of the depletion.

This strategy is illustrated in panels **A/A’, B/B’** and **C/C’** of Figure 11. Panels **A, B** and **C** show the dynamics of accumulation of *i*Aβ and progression of disease in untreated patients with different relative extents of the **T^0^** and **T1** thresholds in three principal variants of AD/AACD discussed above. In panel **A** of Figure 11, the crossing of the **T1** threshold triggers the commencement of AD. In panel **A**’ a transient treatment is administered prior to the **T1** crossing, which depletes *i*Aβ. Following the depletion, AβPP-derived *i*Aβ accumulation resumes from a low baseline and its levels, as shown, would not reach the **T1** threshold within the lifetime of the treated individual. No **T1** crossing would take place, no AβPP-independent *i*Aβ production would be activated, no AD would occur. 

In panel **B** of Figure 11, AD is preceded by AACD. In panel **B**’, a transient *i*Aβ depletion treatment is implemented before the **T^0^** threshold crossing. The de novo accumulation of AβPP-derived *i*Aβ resumes from a low baseline. The **T^0^** (and **T1**) threshold is not crossed within the remaining lifetime of the treated individual, nor does AACD (and AD) occur. 

In panel **C** of Figure 11, the **T^0^** threshold is crossed and AACD is triggered but the **T1** threshold is not reached within an individual’s lifespan. In panel **C**’, a transient *i*Aβ depletion treatment is applied to AACD patient *after* the **T^0^** but prior to the **T1** threshold crossing. *Following the depletion, iAβ levels are well below the **T^0^** threshold and the patient is technically cured of AACD* (subject to complete recovery of the affected neurons following the *i*Aβ depletion treatment). As shown, de novo accumulating AβPP-derived *i*Aβ does not reach the **T^0^** threshold, and AACD does not recur within the remaining lifetime of the treated patient.

The transient *i*Aβ depletion treatment option is clearly the preferable as well as the enactable one: Just find a way, any way, to transiently deplete *i*Aβ. It is a “one better” scenario versus the mode of operation of the Icelandic AβPP mutation. Indeed, the latter is physiologically constrained because the physiology, being inertial and limited to “continuous” processes, is incapable of sharply discontinuous transient actions available to us as illustrated in panels **A’, B’** and **C**’ of Figure 11. The identical end-results, in terms of the prevention of the **T1** and **T^0^** thresholds crossing and, consequently, of the occurrence of AD and AACD, are reached in panels **A’** and **B**’ of Figure 11 versus panels **A’** and **B’** of Figure 10, but these results are achieved with the drastic disparity in the duration of the treatment. 

The duration of the *i*Aβ depletion treatment is defined by the desired extent of depletion and potentially could be as short as few days, a regimen possibly akin to that of an antibiotic treatment. Importantly, the *i*Aβ depletion does not need to be complete to be effective; any reduction in its baseline would be therapeutically meaningful and beneficial (in proportion to the extent of the depletion) because it would increase the duration of time required for the crossing of the **T^0^** and **T1** thresholds and for the occurrence of AACD and AD, thus causing, if not the prevention, then at least a delay in the commencement of a disease. Moreover, the same therapeutic strategy, i.e., transient *i*Aβ depletion treatment, is also applicable to symptomatic stages of both AD and is discussed and illustrated in following sections below. 

As for the treatment of AACD patients (panels **C**’ of Figure 10 and Figure 11), whereas the suppression of the rate of accumulation of AβPP-derived *i*Aβ (Figure 10) can at best stop the progression of the disease, sufficient *i*Aβ depletion (Figure 11) is potentially capable of curing the condition. Drugs suppressing AβPP-derived *i*Aβ accumulation are likely incapable of its sufficiently deep depletion [3] and a principally different type of drugs could be necessitated. Such type of AD/AACD drugs, capable of targeted degradation of *i*Aβ, as well as potential outcomes of its implementation, is discussed in the following sections below.

## 18. ACH2.0-Based Therapeutic Strategy for Treatment of AD at Its Symptomatic Stages

The preceding section described therapeutic strategies for the prevention of AD and AACD and for the treatment of AACD in symptomatic patients. These strategies are based on the ACH2.0 interpretation of both conditions and guided by our understanding of the mechanism of action of the protective Icelandic Aβ mutation. As discussed in the present section, the same guiding principles can be also applied for achieving the, arguably, ultimate goal: Effective treatment of AD at its symptomatic stages. To better orient the reader, a compendium of potential therapeutic options for symptomatic AD is briefly analyzed as follows.

### 18.1. Therapeutic Options for the Symptomatic Stages of AD 

#### 18.1.1. Approaches That Failed or Are Impractical

As was mentioned above, a number of ACH-based candidate AD drugs were developed and performed spectacularly in preclinical studies. They all failed as spectacularly in human clinical trials when administered at various symptomatic stages of AD. In the framework of the ACH2.0, this failure was inevitable because, in this paradigm, the occurrence of AD symptoms signifies that the AβPP-independent, self-sustaining *i*Aβ generation pathway had been activated in most or in all affected neurons (the recently observed effect of lecanemab and donanemag in symptomatic AD appears to be due to the early timing of its administration; the drugs target preventively a marginal subset of affected neurons that did not yet reach and cross the **T1** threshold, hence their marginal effect [3]). At this point the contribution of the AβPP proteolytic pathway into neuronal *i*Aβ pool is rendered negligible and insignificant (in comparison with the contribution of the AβPP-independent *i*Aβ generation pathway) and any attempted interference with this pathway or with secreted Aβ produced in this pathway would be futile [1,2,3]. 

The consequence of the above considerations is that the only potentially successful therapeutic strategy subsequent to symptomatic manifestation of AD is targeting the AD Engine or the components thereof. The components of the AD Engine are lucidly depicted in Figure 1 above. They include *i*Aβ, a mediator in the self-perpetuating cycle that constitutes the AD Engine; the compounds and processes necessary for the initiation of the AβPP-independent *i*Aβ production, such as the *i*Aβ-mediated activation of eIF2α kinases; the integrated stress response; and, finally, the AβPP-independent pathway of *i*Aβ generation. The rationale for the AD-Engine-targeting strategy is obvious. The principal product of the AD Engine is *i*Aβ. It not only propagates the activity of the Engine but also drives the AD pathology. A successful interference with the operation of the AD Engine would, therefore, not only cease the *i*Aβ production in the AβPP-independent pathway but would also interrupt the progression of the disease.

Implementing this strategy, however, is not simple. Inhibiting cellular pathways that are required for and result in the initiation of the AβPP-independent production of *i*Aβ would be quite demanding either because of the built-in redundancies or due to the principal function of a putative target in normal cellular physiology. For example, the ISR was shown to be elicited in neuronal cells by the activated PKR and HRI kinases via the phosphorylation of eIF2α. To suppress the latter, both PKR and HRI need to be inhibited. However, even if the concurrent inhibition of both kinases were feasible, it would be unproductive in preventing phosphorylation of eIF2α because such inhibition would result in a compensatory activation of alternative eIF2α kinases [63]. Interfering with the eIF2α to P-eIF2α conversion upstream of the ISR via the manipulation of PP1 phosphatase, CReP, or GADD34 [63] is not feasible because of the principal role of eIF2α in cellular functioning. Likewise, and for the same reason not feasible is the interference with the downstream ISR targets such as ATF4, ATF5 and CHOP transcription factors [63].

Targeting the AβPP-independent *i*Aβ generation pathway is also problematic for more than one reason. First, the nature of the mechanism enabling this pathway is not understood with sufficient certainty; whereas the asymmetric amplification of AβPP mRNA is the most likely possibility (see below), the three other mechanisms described below are also optionally valid [1]. The second reason is that the underlying mechanism could be physiologically vital and cannot be interfered with by a broad approach. Thus, for example, mRNA amplification in mammalian cells was shown to be crucial for multiple fundamental cellular functions [104,105,106,107] and, as such, cannot be manipulated without a probable detrimental effect. One plausible way to interfere narrowly and *specifically* with the AβPP-independent *i*Aβ production (regardless of the nature of the underlying mechanism) is via site-specific intervention at the ATG encoding Met671 of AβPP and/or surrounding nucleotides [1]. If the initiation of translation from this position were disabled, no AβPP-independent *i*Aβ production would occur (see details below). This approach, presumably through genome editing, is, however, currently unfeasible in humans (but can be used in experimental models). It should be also noted that even if the disabling of the AβPP-independent *i*Aβ production pathway were feasible, it would not interfere in any way with the occurrence of AACD, which is driven solely by AβPP-derived *i*Aβ because in such a case, as described above, AACD would commence when AβPP-derived *i*Aβ levels reach the **T^0^** threshold and would continue for the remaining lifespan of an individual.

Therefore, the sole remaining therapeutic option for symptomatic stages of AD is lowering the levels of *i*Aβ below those needed for the activation of the AβPP-independent *i*Aβ production pathway and, consequently, for the operation of the AD Engine. An apparent logical approach toward this goal is the suppression of the activity of gamma-secretase. This would alter the generation of *i*Aβ not only by AβPP proteolysis but also in the AβPP-independent pathway (see details below) and would eventually lower its levels. This strategy was tried, with detrimental results, in multiple studies, including clinical trials [108,109,110,111], and was eventually forsaken. The reasons for this failure eventually become clear: gamma secretase is an important member of the Notch pathway with many C99-unrelated substrates and, therefore, cannot be interfered with without deleterious consequences. Modulating gamma-secretase activity with the goal of producing shorter, more benign isoforms of Aβ has also proven so far less than satisfactory [110,111,112,113].

#### 18.1.2. Potentially Feasible Therapeutic Strategy: Activation of Alpha-Secretase

Another strategy to lower levels of *i*Aβ is the activation of alpha-secretase. This would increase the cleavage within Aβ, at its lysine 16, and, consequently, would both reduce the rate of *i*Aβ production and deplete its preexisting pool. This possibility was addressed [114,115,116,117,118,119] and the evidence of therapeutic benefits of such an approach has been obtained in multiple studies [119,120,121,122]. This strategy, however, is burdened with potential complications of the same type that invalidated therapeutic application of gamma-secretase inhibitors. This is because alpha-secretase belongs to the ADAM family of proteases. Its exogenous overexpression in cellular models (i.e., the increase in its activity) affected more than three hundred genes [123]. Moreover, it appears that alpha-secretase is involved in certain Notch-controlled pathways [124]. These considerations explain the well-justified prudence with advancing the utilization of alpha-secretase-activating agents as potential AD therapy.

### 18.2. Activation of Aβ-Cleaving Activities of BACE1 and/or BACE2: A Rational, Intuitive and Feasible Therapeutic Option in the ACH2.0 Perspective

There is, however, one potential therapeutic option that offers all benefits described above but without associated disadvantages. In this approach, the stated goal of lowering *i*Aβ level, and potentially substantially depleting it, is achieved by its targeted degradation via the activation of Aβ-cleaving capabilities of one or both variants of beta-secretase, BACE1 and BACE2. In view of the previously attempted extensive utilization of BACE1 inhibitors as potential AD drugs, a project that required tremendous investment of funds, research, and development efforts, the proposed use of the diametrically opposite strategy, namely the employment of BACE1 and BACE2 activators, may appear radical and counterintuitive. It is, however, neither. *The proposed utilization of BACE1/BACE2 activators (or, in fact, of any other suitable iAβ-depleting agent) is a no less justifiable, rational, logical, intuitive and feasible AD therapy in the ACH2.0 paradigm than the attempted employment of BACE inhibitors was (when it was proposed and implemented) in the ACH perspective.*

To appreciate the above assertion, consider the following brief summary of the relevant empirical data [4,5,125,126,127,128]:

(**a**) It was demonstrated that BACE1 cleaves not only at the β site, thus generating the N-terminus of C99 and of Aβ, but also at the β′ site ten residues downstream. (**b**) BACE1 cleavage at the β′ site occurs equally efficiently within AβPP, C99 and Aβ. (**c**) Exogenous overexpression of BACE1 in mouse models increased the rate of cleavage at the β′ site as well as the ratio of the N-truncated versus full-size Aβ. (**d**) Exogenous overexpression of human BACE1 substantially decreased Aβ deposition in mouse brain. (**e**) As described above, the protection conferred by the Icelandic AβPP mutation upon its carriers is apparently due to the elevated rate of BACE1 cleavage at the β′ site. 

In addition to the β′ site cleavage, multiple studies [129,130,131,132] demonstrated that BACE1 cleaves also at residues 34/35 of human Aβ; the rate of this cleavage increased significantly when BACE1 was overproduced exogenously. The BACE1 cleavage at residues 34/35 of Aβ produces Aβ_34_, an intermediate in Aβ clearing. It appears, therefore, that a sufficient elevation of Aβ-cleaving activities of BACE1 is capable of depleting neurons of iAβ, thus ceasing the progression of AD if implemented at symptomatic stages or preventing its occurrence if employed prior to manifestation of AD symptoms.

BACE2 activation could be equally, if not more effective in depleting *i*Aβ in affected neurons. Whereas it is capable of cleavage at the β site of AβPP, its *main* activity is to cleave *within* Aβ in two positions, at residues 19 and 20 (both phenylalanines) [102]. It appears that the physiological role of BACE2 is to limit the generation of Aβ. When BACE2 is inhibited in model systems, the production of Aβ substantially increases [103]. This BACE2 function appears to be a naturally occurring protective mechanism, a notion that is strongly supported by the effect of the Flemish FAD mutation at the residue 21 of Aβ. The Flemish FAD mutation suppresses the capacity of BACE2 to cleave within Aβ. This results in the elevation of *i*Aβ levels in mutation carriers and, consequently in the early onset of AD [62]. 

Therapeutically, the activation of BACE2 at the symptomatic stages of AD would deplete *i*Aβ and stop the progression of the disease. When implemented prior to manifestation of the symptoms, it would prevent the disease. This strategy constitutes, in fact, the augmentation of the physiological protective function of BACE2. Since the cleavage within Aβ (rather than at the β site) is the predominant activity of BACE2 and because it appears to be employed physiologically in the protective role, the employment of BACE2 activators is, apparently, physiologically more suitable than that of BACE1 enhancers. If only one, BACE1 or BACE2, protease can be manipulated in the therapeutic application, the utilization of BACE2 activation seems more advantageous. On the other hand, it would be most efficient in concert with the activation of BACE1. This is because the two not only target discrete Aβ sites but are also situated in distinct subcellular locations [133].

Potentially, a treatment, which activates Aβ-cleaving capabilities of BACE1- and/or BACE2 and is administered for only limited duration, could accomplish a sufficient depletion of *i*Aβ and thus open the possibility of once-in-a-lifetime-only curative or preventive therapy for AD (as described in Section 17.2 above, it would be no less efficient in the prevention and treatment of AACD). This possibility is further discussed and illustrated in the following section below.

## 19. AD Therapy at Symptomatic Stages: Once-in-a Lifetime Transient *i*Aβ Depletion Therapy via Its Targeted Degradation Would Potentially Stop the Progression of the Disease

As described above, the AD Engine, i.e., the AβPP-independent *i*Aβ production pathway, which drives the disease, requires certain levels of *i*Aβ for its activation and operation. These levels are maintained by the continuous influx of *i*Aβ generated in the AβPP-independent pathway and, in turn, sustain and perpetuate the operation of the pathway and of the Engine. The goal of the proposed *i*Aβ depletion therapy at the symptomatic stages of AD is to bring *i*Aβ levels below the **T1** threshold, the *i*Aβ level required for the activation of the AβPP-independent *i*Aβ production pathway. When this happens, the AβPP-independent *i*Aβ production would be switched off, the influx of *i*Aβ generated in this pathway would cease, the AD Engine would be rendered inoperative, the progression of the disease would stop and AD-affected neurons that remained viable would be allowed to recover and reconnect. The depletion of *i*Aβ would not affect, however, the AβPP proteolytic pathway; it would remain operational regardless of the *i*Aβ levels. If the *i*Aβ depletion treatment were transient, the duration of its effect would be identical to the time interval required for the restoration of *i*Aβ (produced at this point solely in the AβPP proteolytic pathway) to the **T1** threshold levels, for the consequent re-activation of the AβPP-independent *i*Aβ production pathway and the AD Engine, and for the recurrence of the disease. The therapeutic efficiency of the transient *i*Aβ depletion treatment would, therefore, directly depend on the degree of depletion: the “deeper” it is, the more time is required for the restoration of the levels of AβPP-derived Aβ to the **T1** threshold and consequent activation of the AβPP-independent *i*Aβ production pathway and the recurrence of the disease. Ultimately, with the *i*Aβ depletion sufficiently “deep”, the duration required for the de novo accumulation of AβPP-derived *i*Aβ to the **T1** threshold would exceed the remaining lifespan of a patient and the disease would not recur.

**Figure 12:**
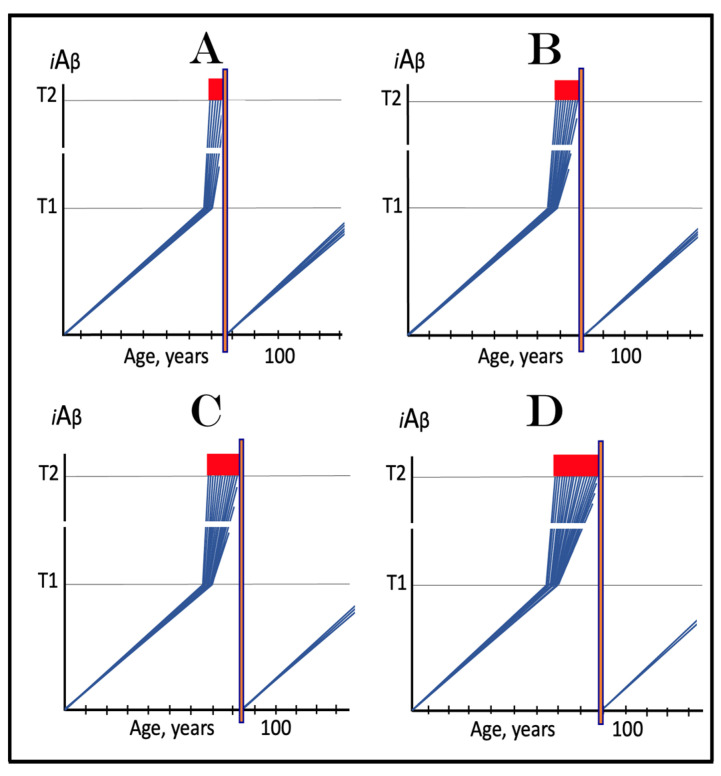
Effect of transient *i*Aβ depletion therapy via its targeted degradation at various symptomatic stages of AD. Blue lines: affected neurons. *iA*β: Level of intraneuronal Aβ. ***T1*** *threshold*: Levels of *i*Aβ triggering elicitation of the ISR, initiation of AβPP-independent production of *i*Aβ, activation of the AD Engine, and the commencement of the second stage of AD. ***T2*** *threshold*: Levels of *i*Aβ triggering neurons’ entrance into the apoptotic pathway. *Red blocks*: Apoptotic zone. *Orange boxes:* Active transient *i*Aβ depletion via its targeted degradation by Aβ-cleaving activities of BACE1 and/or BACE2 or by any other suitable agent; levels of *i*Aβ are reset and the accumulation of AβPP-derived *i*Aβ resumes from a low baseline. *Panel **A***: The transient *i*Aβ depletion therapy is implemented at the early symptomatic stage of AD, when the bulk of the affected neurons are still viable. Following the reset of *i*Aβ levels, its build-up starts de novo, supported only by the AβPP proteolytic pathway. It is anticipated that *i*Aβ levels will not reach the **T1** threshold and AD will not recur within the remaining lifetime of an SAD patient. *Panels **B, C**, and **D***: The transient *i*Aβ depletion treatment is implemented at progressively advanced stages of AD. The results are analogous to those depicted in panel **A**. However, at this AD stages increasing number of affected neurons cross the **T2** threshold and commit apoptosis. This leaves a progressively smaller number of affected neurons that retained their viability and can be redeemed.

Figure 12 illustrates the effects of *i*Aβ depletion therapy administered at various symptomatic stages of AD. In this Figure, it is presumed that the transient elevation of Aβ-cleaving activities of BACE1 and/or BACE2, or the limited-duration employment of any other appropriate *i*Aβ depletion agent, results in complete or nearly complete (sufficiently “deep”, as discussed above) depletion of *i*Aβ. It is also envisioned that the rate of accumulation of *i*Aβ produced in the AβPP proteolytic pathway and the extent of the **T1** threshold following *i*Aβ depletion therapy are similar to the same values prior to the depletion treatment. As depicted in Figure 12, the *i*Aβ depletion treatment is implemented either when symptomatic manifestation of the disease has just commenced (panel **A**) or at more and more advanced stages of AD (panels **B** through **D**). At each stage depicted in the figure, the transient administration of the *i*Aβ depletion therapy results in a “deep” reset of the level of *i*Aβ in neurons that survived and remained viable. 

At the early symptomatic stage of AD (panel **A** of Figure 12), this category includes the majority of the affected neurons. Following the reset of the *i*Aβ levels, the activity of the AβPP-independent *i*Aβ production pathway ceases, operation of the AD Engine stops, and viable affected neurons are allowed to recover and reconnect. The production of *i*Aβ at this stage occurs only in the AβPP proteolytic pathway. The accumulation of newly produced *i*Aβ commences from a low baseline, and its build-up to the **T1** threshold would be of a long duration, exceeding that of the remaining lifespan of a patient. Consequently, the AβPP-independent *i*Aβ generation pathway would not be activated, the AD Engine would remain inoperative and the AD would not recur within patient’s lifetime. Since the majority of AD-affected neurons would be redeemed, the prognosis for patient stabilization and, possibly, a significant cognitive recovery would be good.

With the progression of AD, at its more advanced stages, increasing number of the affected neurons cross the **T2** threshold and commit apoptosis. This leaves progressively smaller number of the affected neurons that retained their viability and can be redeemed. This progression is depicted in panels **B, C**, and **D**. The administration of transient *i*Aβ depletion therapy and the following reset of the *i*Aβ baseline would result in inactivation of the AβPP-independent *i*Aβ production pathway, and would allow the ever decreasing number of viable AD-affected neurons to recover and restore their functionality. At this point, the prospect of stopping the progression of the disease is, apparently, as good as at the early stages of the disease, but cognitive functions would be increasingly unlikely to be significantly restored; the probability of such an occurrence would be proportional to the fraction of the affected neurons that were redeemed by *i*Aβ depletion via its targeted degradation by the Aβ-cleaving activities of BACE1 and/or BACE2 or by any other suitable agent. As reasoned above, following the *i*Aβ depletion, the disease would not recur within the remaining patient’s lifespan. 

## 20. Dynamics of *i*Aβ Accumulation and of the Disease at Symptomatic AD Stage

Above, we analyzed the dynamics of accumulation of AβPP-derived *i*Aβ and the role of the extents of the **T^0^** and **T1** thresholds in the commencement of AACD and of the second, symptomatic, stage of AD. The event that signifies and defines the second AD stage is the activation of the self-perpetuating AβPP-independent *i*Aβ production pathway, which drives the disease [1,2,3]. At this stage, the accumulation of *i*Aβ produced by AβPP proteolysis continues, presumably at the same rate as prior to the crossing of the **T1** threshold, but now it is rendered marginal and inconsequential (due to its now marginal contribution into the *i*Aβ pool) because the entire output of Aβ produced independently of AβPP is retained intraneuronally and perpetuates the operation of AβPP-independent *i*Aβ generation pathway, i.e., its own production [1,2,3]. Whereas at the first, asymptomatic, AD stage, the AβPP-derived *i*Aβ-initiated cascade is relatively benign and involves the activation of eIF2α kinases and elicitation of the ISR, at the second AD stage *i*Aβ produced independently of AβPP drives a much more perilous cascade that involves the formation, presumably through a chain of events, including the *i*Aβ-mediated inhibition of the ubiquitin–proteasome system [134,135,136,137], of tau tangles, and ultimately results in neuronal loss. 

The factors determining the dynamics of *i*Aβ accumulation and of the disease at the second AD stage are superficially similar to those operating at the first stage of AD: a rate of accumulation and the extent of a threshold. But at the second AD stage, these parameters are the rate of accumulation of *i*Aβ produced independently of AβPP (rather than of AβPP-derived *i*Aβ) and the extent of the **T2** (rather than of **T1**) threshold, which is a “point of no return” since its crossing triggers the apoptotic pathway. Accordingly, the timing of the end stage of the disease is inversely proportional to the rate of *i*Aβ accumulation and directly proportional to the extent of the **T2** threshold. Indeed, the higher the rate of *i*Aβ accumulation, the faster the progression of AD and the sooner would the end stage be reached; the higher the extent of the **T2** threshold, the greater the timing of the occurrence of the end stage of the disease.

**Figure 13:**
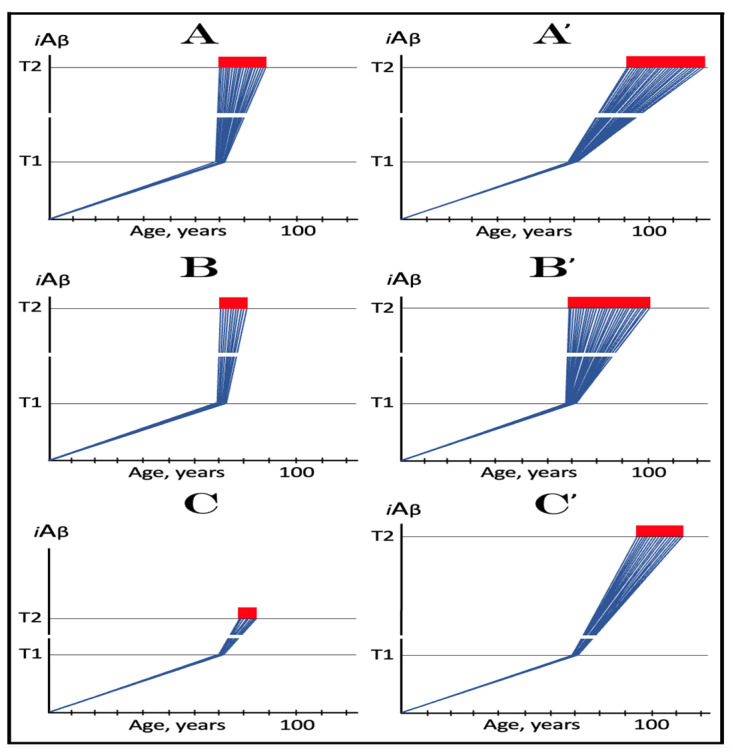
Dynamics of *i*Aβ accumulation and of the disease at the second, symptomatic stage of AD. *Blue lines*: Affected neurons. *iA*β: Levels of intraneuronal Aβ. ***T1*** *threshold*: Levels of *i*Aβ triggering elicitation of the ISR, initiation of AβPP-independent production of *i*Aβ, activation of the AD Engine and the commencement of the second stage of AD. ***T2*** *threshold*: Levels of *i*Aβ triggering neurons’ entrance into the apoptotic pathway. *Red blocks*: Apoptotic zone. All kinetic parameters up to and including the crossing of the **T1** threshold are identical in all panels whereas the kinetic parameters following the **T1** crossing and the commencement of the second AD stage are different. In panels **A** and **A’,** the extent of the **T2** threshold is the same but the rates of accumulation of *i*Aβ produced in the AβPP-independent *i*Aβ production pathway are different. It is much greater in panel **A** than in panel **A’**. Accordingly, the rate of progression of the disease is much slower, the timing of its symptomatic manifestation is significantly greater, and its duration is substantially longer in panel **A’** than in panel **A.** In panels **B** and **B’,** both the extent of the **T2** threshold and the initial (fastest) rate of accumulation of *i*Aβ produced independently of AβPP are identical but the stochastic distribution of the latter in the affected neurons is much wider in panel **B’** than in panel **B**. Accordingly, the duration of the disease is significantly longer in panel **B’** than in panel **B**. In panels **C** and **C’,** the rate of accumulation of *i*Aβ produced in the AβPP-independent *i*Aβ production pathway and it’s stochastic distribution in the affected neurons are the same but the extents of the **T2** threshold differ. In panel **C’,** it is substantially higher than in panel **C**. Consequently, the timing of the symptomatic manifestation of the disease is greater and the duration of the disease is significantly longer in panel **C’** than in panel **C**.

These relationships are presented diagrammatically in Figure 13. In this Figure, the kinetic parameters of AβPP-derived *i*Aβ accumulation up to and including the crossing of the **T1** threshold are identical in all panels whereas the kinetic parameters following the **T1** crossing and the commencement of the second AD stage are different. In panels **A** and **A**’, the extent of the **T2** threshold is the same, but the rates of accumulation of *i*Aβ produced in the AβPP-independent *i*Aβ production pathway are different. It is much greater in panel **A** than in panel **A**’. Accordingly, as discussed above, the rate of progression of the disease is much slower, the timing of its symptomatic manifestation is significantly greater, and its duration is substantially longer in panel **A**’ than in panel **A**. 

In panels **B** and **B’**, both the extent of the **T2** threshold and the initial (fastest) rate of accumulation of *i*Aβ produced independently of AβPP are identical but the stochastic distribution of the latter in the affected neurons is much wider in panel **B’** than in panel **B**. Accordingly, the duration of the disease is significantly longer in panel **B**’ than in panel **B**. In panels **C** and **C**’, the rate of accumulation of *i*Aβ produced in the AβPP-independent *i*Aβ production pathway and it stochastic distribution in the affected neurons are the same, but the extents of the **T2** threshold differ. In panel **C’**, it is substantially higher than in panel **C**. Consequently, the timing of the symptomatic manifestation of the disease is greater and the duration of the disease is significantly longer in panel **C**’ than in panel **C**. 

None of the kinetic parameters discussed in the present section and illustrated in Figure 13 have a conceptual impact on the proposed therapeutic strategy at symptomatic stages of AD, namely the *i*Aβ depletion via its targeted degradation by the Aβ-cleaving activities of BACE1 and/or BACE2 or by any other suitable agent. On the other hand, their combined variability offers a plausible explanation for a well-documented phenomenon: a sequential manifestation of AD pathology in the defined rejoins of the affected brain. This aspect of the disease is discussed in the following section below.

## 21. Sequential Manifestation of the AD Pathology in Defined Brain Compartments. Implications for the *i*Aβ Depletion Therapy at the Early Symptomatic Stages of AD

### 21.1. Rate of Accumulation of iAβ Produced Independently of AβPP May Differ in Diverse Regions of the Affected Brain

It could be presumed that the stochastically distributed (in individual AD-affected neurons) rate of accumulation of *i*Aβ produced in the AβPP-independent *i*Aβ generation pathway in the second, symptomatic stage of AD and the extent of the **T2** threshold are patient-specific and identical throughout the affected brain. This assumption would imply that the rate of temporal progression of the AD pathology is also the same throughout the AD-affected brain. This implication, however, is patently invalid because one of the principal documented features of AD is the temporally sequential nature of the occurrence of the AD pathology in the various defined regions of the affected brain.

Above, we concluded, on the basis of the available empirical data, that in the majority of, if not in all, AD-affected neurons, *i*Aβ levels reach and cross the **T1** threshold within a narrow time window. This means that the second stage of AD commences within close temporal proximity in all neurons affected by the disease. Yet, it is the basic knowledge that the AD pathology occurs in different defined parts of the brain as a widely distributed temporal function. Indeed, anatomical and histological studies of AD-affected brains concluded that the neurodegeneration begins within the second layer of the entorhinal cortex followed by its occurrence in the hippocampus, temporal cortex, frontoparietal cortex, and subcortical nuclei [138]. Moreover, each compartment of the brain exhibits the neuropathology in the gradual and defined manner. For example, in the hippocampus, the CA1 area is affected first, followed sequentially by the areas CA2, CA3 and DG [139].

The question is then, how to reconcile the relatively concurrent **T1** crossings and the commencement of the second AD stage in all affected neurons, throughout the entire brain, with the widely spread, over many years, effects of the disease in various compartments of the brain? One plausible answer lies in the variably wide stochastic distribution of the rate of accumulation of *i*Aβ produced independently of AβPP in and, consequently, of the distribution of the **T2** threshold (assumed in this interpretation to be the same throughout the affected brain) crossings by the affected neurons, postulated in the preceding section. It necessitates that different (yet overlapping) segments of the overall stochastic distribution spectrum represent affected neurons from different, distinctly defined parts of the brain; i.e., *i*Aβ crosses the **T2** threshold sequentially in diverse regions of the AD-affected brain. For this to occur, the average rate of *i*Aβ accumulation should differ in different parts of the brain due to either diverse, brain compartment-specific efficiencies of the AβPP-independent *i*Aβ generation pathway or varied rates of *i*Aβ clearing. In each defined brain compartment, the stochastic nature of the **T2** threshold crossings would be local yet overlapping to form the overall spectrum that defines the duration of AD.

**Figure 14:**
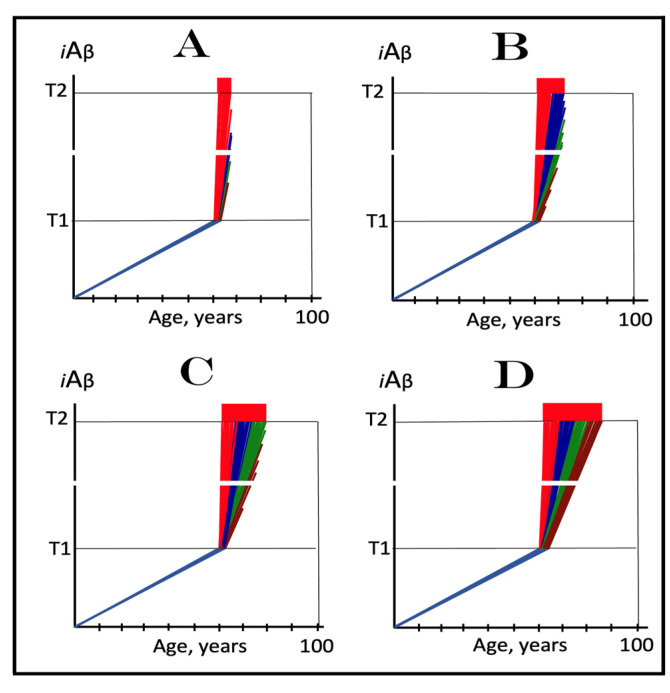
Sequential manifestation of the AD pathology in defined brain compartments: The rate of accumulation of *i*Aβ produced independently of AβPP differs in diverse regions of the affected brain. *iA*β: Level of intraneuronal Aβ. ***T1*** *threshold*: Level of *i*Aβ level triggering elicitation of the ISR, initiation of AβPP-independent production of *i*Aβ, activation of the AD Engine and the commencement of the second stage of AD. ***T2*** *threshold*: Levels of *i*Aβ triggering neurons’ entrance into the apoptotic pathway. *Red blocks*: Apoptotic zone. Each line represents individual affected neurons. *Lines of different colors above the **T1** threshold*: The affected neurons in various defined parts of the AD-afflicted brain. The rate of *i*Aβ accumulation differs in different parts of the brain, due to either diverse, brain compartment-specific, efficiencies of the AβPP-independent *i*Aβ generation pathway or varied rates of *i*Aβ clearing. *Panel **A***: The early symptomatic stage of AD. Only the entorhinal cortex and possibly the hippocampus are affected; neither significant accumulation of *i*Aβ produced in the AβPP-independent pathway, nor AD neuropathology yet occurred in other brain compartments. *Panels **B**, **C**, **D***: With the progression of AD toward the end stage (panel **D**), *i*Aβ produced in the AβPP-independent pathway accumulates and the AD pathology commences and expends in temporally sequential manner in other defined compartments of the affected brain. Note that if the therapeutic intervention, via transient administration of BACE1 and/or BACE2 activators or of other *i*Aβ-depleting agents, were implemented at an early symptomatic stage of AD (panel **A**), the progression of the disease in the brain compartment already affected at this stage would cease, and the AD pathology would not commence, due to *i*Aβ depletion, in other brain compartments, which would remain largely intact. The progression of AD in the affected brain compartment would not resume and other brain compartments would stay pathology-free for the remaining lifespan of a patient.

This notion is illustrated in Figure 14. In this Figure, the lines of different colors above the **T1** threshold represent *i*Aβ levels in the affected neurons in defined regions of the afflicted brain whereas panels **A** through **D** of Figure 14 provide diagrammatic snapshots of progressive stages of AD. Panel **A** of Figure 14 illustrates an early AD stage. Only a fraction of the affected neurons in only a single defined brain compartment reached and crossed the **T2** threshold and committed apoptosis. In panels **B** and **C**, as the disease progresses, all affected neurons in the first (red) compartment cross the **T2** threshold and commit apoptosis, and similar accession toward and crossing of the **T2** threshold occurs sequentially in the defined compartments of the affected brain. In panel **D**, all affected neurons reached and crossed the **T2** threshold in all defined compartments of the afflicted brain; this is the end stage of the disease.

The above interpretation of the sequential manifestation of AD pathology in the defined regions of the affected brain has an important implication for treatment of the disease. If the transient *i*Aβ depletion therapy via its targeted degradation by Aβ-cleaving activities of BACE1 and/or BACE2 or by any other suitable *i*Aβ-depleting agent were implemented at an early symptomatic stage of AD, the progression of the disease in the early-affected brain compartment (e.g., panel **A** of Figure 14) would cease, and the AD pathology *would not* occur, due to *i*Aβ depletion, in other brain compartments where it did not yet commence or progressed only insignificantly. *In the early-affected brain region, the disease would not recur (for the reasons discussed in* Section 19*), and other brain compartments would stay pathology-free for the remaining lifespan of a patient.*

### 21.2. An Alternative Interpretation of Sequential Manifestation of AD Pathology: The Extent of the **T2** Threshold May Differ in Diverse Defined Regions of the Affected Brain 

**Figure 15:**
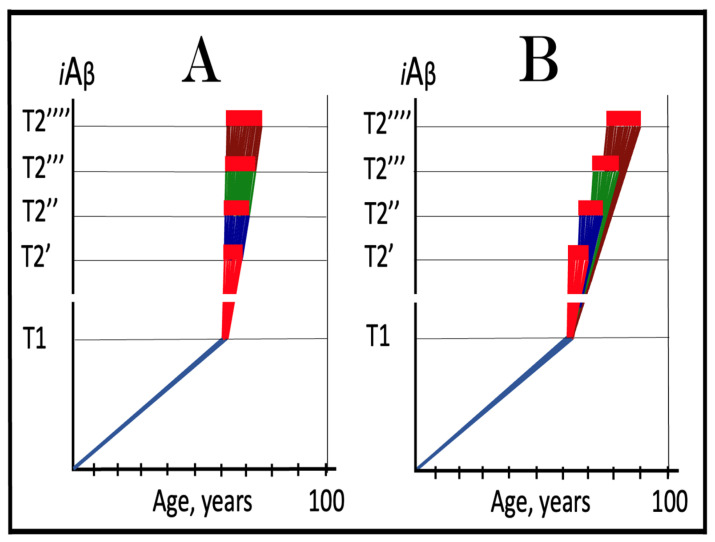
Sequential manifestation of the AD pathology in defined brain compartments: The extent of the **T2** threshold is variable, either separately or simultaneously with the rate of AβPP-independent *i*Aβ accumulation, in distinct defined regions of the affected brain. *iA*β: Level of intraneuronal Aβ. ***T1*** *threshold*: Level of *i*Aβ level triggering elicitation of the ISR, initiation of AβPP-independent production of *i*Aβ, activation of the AD Engine, and the commencement of the second stage of AD. ***T2*** *threshold*: Levels of *i*Aβ triggering neurons’ entrance into the apoptotic pathway. *Red blocks*: Apoptotic zone. Each line represents individual affected neurons. ***T2’*** *through **T2’’’’***: Extents of the **T2** threshold in separate define brain compartments. *Lines of different colors above the **T1** threshold*: The affected neurons in various defined parts (signified by different colors) of the AD-afflicted brain. Dynamics of *i*Aβ accumulation and the disease in separate defined brain regions are superimposed. Levels of AβPP-derived *i*Aβ reach and cross the T1 threshold in a narrow temporal window in all affected neurons throughout the entire brain. Following the T1 crossing, the bulk of *i*Aβ is produced in the AβPP-independent pathway. *Panel **A***: The rate of AβPP-independent *i*Aβ accumulation and its stochastic distribution are identical throughout the entire AD-affected brain, but extents of the **T2** threshold are different in diverse defined brain compartments. The **T2** threshold is reached and the affected neurons commit to apoptosis and die at different times in different brain regions; consequently, the AD pathology manifests in a sequential temporal order. *Panel **B***: Both the rate of AβPP-independent *i*Aβ accumulation and the extent of the **T2** threshold are variable in separate defined regions of the affected brain and both contribute to sequential temporal manifestation of the AD pathology by determining the timing of its occurrence. Note that the extents of temporal shifts (e.g., in the **T2** threshold crossings) could be significantly greater when both parameters are variable in defined regions of the brain. The depicted inverse proportionality between rates of AβPP-independent *i*Aβ accumulation and extents of the **T2** threshold (panel **B**) is shown for purposes of comparison and graphic convenience only; it is just one of multiple possible combinations of these two parameters in various defined regions of the AD-affected brain.

Assigning different segments of the overall stochastic distribution of the rate of *i*Aβ accumulation in individual affected neurons at the second AD stage to different defined compartments of the brain is not the only scenario capable of explaining temporally sequential occurrence of the AD pathology in the affected brain. Another potential scenario is that in symptomatic AD *i*Aβ levels in affected neurons increase toward the **T2** threshold with the same rate and same stochastic distribution in all brain regions, but the extent of the **T2** threshold differs in different brain compartments. This scenario is illustrated in panel **A** of Figure 15, which shows the dynamics of *i*Aβ accumulation toward the **T2** threshold in different regions in the AD-affected brain (different brain compartments are signified by different colors). The rate of AβPP-independent *i*Aβ accumulation and its stochastic distribution are identical throughout the entire AD-affected brain, but the extents of the **T2** threshold are different in diverse defined brain compartments. Consequently, the **T2** threshold is reached and the affected neurons commit to apoptosis and die at different times in different brain regions. With the variable extents of the **T2** threshold, the affected neurons commit to the apoptotic pathway in a defined brain region-specific mode, and the AD pathology manifests in a sequential temporal order in defined brain compartments. This scenario explains the sequential appearance of lesions associated with the neuronal death and is consistent with the observed sequential appearance of tau tangles (formed in live cells presumably prior to the **T2** threshold crossing) in various defined regions of the AD-affected brain [138]. 

### 21.3. A Combination of Two Variable Kinetic Parameters Could Be Responsible for Sequential Manifestation of AD Pathology in Defined Brain Regions

On the other hand, it is distinctly possible that the observed sequential temporal occurrence of the AD pathology in defined brain regions involves a combination of variable kinetic parameters, for example, differential rates of AβPP-independent accumulation of *i*Aβ and differential extents of the **T2** threshold in various defined compartments of the AD-affected brain. This combined scenario is shown in panel **B** of Figure 15. In this scenario, both the rate of AβPP-independent *i*Aβ accumulation and the extent of the **T2** threshold are variable in separate defined regions (signified by different colors) of the affected brain, and both contribute to sequential temporal manifestation of the AD pathology by determining the timing of its occurrence. Note that the extents of temporal shifts (e.g., in the **T2** threshold crossings) could be significantly greater when both parameters (rather than only one as shown in panel **A** of Figure 15) are variable in defined regions of the brain. The depicted inverse proportionality between rates of AβPP-independent *i*Aβ accumulation and extents of the **T2** threshold in panel **B** is shown for purposes of comparison and graphic convenience only; it is just one of multiple possible combinations of these two parameters in various defined regions of the AD-affected brain. Importantly, the proposed transient *i*Aβ depletion therapy via its targeted degradation by Aβ-cleaving activities of BACE1 and/or BACE2 activators or by any other suitable *i*Aβ-depleting agent is equally applicable in all scenarios discussed above.

## 22. Cellular Mechanisms Capable of the iAβ Generation Independently of AβPP

Generation of *i*Aβ in the AβPP-independent manner is one of the central tenets of the ACH2.0. Indeed, in this theory of AD, the activation of the self-perpetuating AβPP-independent *i*Aβ production pathway is the pivotal event in the etiology of AD, which marks the commencement of the disease. Because of its presumably vast output, this is the process that, when active, renders the contribution of the AβPP proteolytic pathway to the cellular *i*Aβ pool insignificant for progression of the disease and its targeting for therapeutic purposes futile. This is why the understanding of the mechanism that generates *i*Aβ independently from AβPP is of crucial importance for elucidating the disease. Moreover, as discussed above, because of its presumed role, this mechanism and its components constitute the prime therapeutic targets in AD.

### 22.1. The Centrality of the AUG Codon for Met671 of AβPP in the Presumed AβPP-Independent Production of iAβ 

At least four known cellular mechanisms (see below) are capable of producing Aβ independently of AβPP. As elaborated below, they all share the key common feature: In every conceivable mechanism of AβPP-independent generation of Aβ, translation initiates at the AUG encoding methionine 671 of AβPP. The possibility that, in AD, Aβ is produced independently from AβPP by translation initiating at this AUG codon was first posited by Breimer and Danny in 1987 [140], the year when human AβPP cDNA was synthesized, cloned and sequenced simultaneously by several groups [141,142,143]. In their study [140], Breimer and Danny noted that C99 and Aβ-encoding portion of human AβPP DNA is preceded immediately, contiguously and in-frame by the ATG which encodes methionine 671 of AβPP. Importantly, this ATG is embedded in the optimal translation initiation nucleotide context (Kozak motif), the arrangement exceptional in the human AβPP gene, where of 20 in-frame Met-encoding ATG codons (including the ATG encoding the translation-initiating Met), only the ATG encoding Met671 is found in the nucleotide context optimal for the initiation of translation [140]. 

Breimer and Denny argued [140] that such a favorable and unique positioning of the ATG encoding Met671 of the AβPP may be not random but rather reveals the underlying physiological function. They proposed that this function could be the initiation of translation of AβPP mRNA within its coding region, a mechanism that could be *inducible and operative in AD* (discussed in more detail below). They also argued that translation initiated at the AUG encoding Met671 of AβPP would result in C99 because the initiating Met would be removed co-translationally by N-terminal methionine aminopeptidases 1 and 2 (MAP1 and MAP2); the gamma-cleavage of C99 would, in turn, generate Aβ. Thus, in the Breimer and Danny’s version of events, C99 and Aβ could be produced independently of AβPP but would be identical in all respects to and indistinguishable from their counterparts produced by the AβPP proteolysis [140]. 

### 22.2. C100 (Met-C99) and Met-iAβ Produced Independently of AβPP Can Be Distinguished from Their Counterparts Resulting from the AβPP Proteolysis: The Key to Evaluating the Validity of the ACH2.0

Breimer and Danny’s presumption that C99 and Aβ generated independently of AβPP via the initiation of translation from the AUG encoding Met671 would be indistinguishable from their counterparts produced in the AβPP proteolytic pathway [140] was, however, subsequently proven to be incorrect. Indeed, as detailed below, the ensuing studies of the processing of the N-terminal translation-initiating methionine in eukaryotic cells have shown that MAP1 and MAP2 would be incapable of removing (co-translationally) the translation-initiating methionine preceding C99 and Aβ and that, in this case, the primary translation product would be C100 (i.e., N-terminal Met-C99), which can be processed by gamma-cleavage into Met-Aβ, both readily distinguishable from C99 and Aβ produced in the AβPP proteolytic pathway; eventually, the N-terminal methionine would be removed, but, importantly, *post*- rather than co-translationally and by aminopeptidases other than MAP1/MAP2.

Translation of the bulk of cellular proteins is initiated from N-terminal methionine. This methionine, however, is not always cleaved-off co-translationally by MAP1 and/or MAP2. For this to occur, both N-terminal Met and the residue that follows it should be accommodated within the active site of the enzyme. This is, therefore, a geometric problem and, with the invariable N-terminal Met, the feasibility of cleavage is strictly a function of the size of a residue that follows it [144,145,146]. The size of a residue is directly defined by the radius of gyration (RG) of its side chain. The smallest RG, zero, is in glycine, which does not have a side chain. The RG is 0.77 Angstroms in alanine, 1.08 Angstroms in serine, 1.22 Angstroms in cysteine, 1.24 Angstroms in threonine, 1.25 Angstroms in proline and 1.29 Angstroms in valine. It steadily increases and reaches its highest value (2.38 Angstroms) in arginine. 

The N-terminal Met can be cleaved-off co-translationally by MAP1 and/or MAP2 *only* if it is followed by one of the seven smallest residues listed above [147]. The position following methionine 671 of AβPP is occupied by aspartate (RG 1.43 Angstroms). Consequently, if translation initiates from the AUG codon for methionine 671 of AβPP, this methionine would not be removed co-translationally and the resulting primary product would be not C99 but C100, i.e., Met-C99. In cases such as this, where the translation-initiating methionine is not removed by MAP1/MAP2, it is ultimately cleaved-off by one of numerous aminopeptidases with a broad specificity [148], as happens, for example, with γ-actin where the penultimate residue is glutamate, with RG of 1.77 Angstroms, and where the N-terminal methionine is cleaved–off by an aminopeptidase distinct from MAP1/MAP2 [149]. It should be emphasized that the cleavage of translation-initiating methionine by an aminopeptidase that is not MAP1/MAP2 does invariably occur post-translationally.

It follows, in light of the above considerations, that if *i*Aβ is generated in AD independently of AβPP by initiation of translation from the AUG encoding methionine 671 of AβPP, pools of Met-C99 (i.e., C100) and, potentially, of Met-Aβ would occur in the affected human neurons. These pools would represent the equilibrium of several dynamic processes, namely gamma-cleavage of C100 yielding Met-Aβ, conversion of C100 into C99 via the removal on the N-terminal methionine by aminopeptidases other than MAP1/MAP2, and conversion of Met-Aβ into Aβ trough the same mechanism. Relative rates of these dynamic processes would define sizes of the Met-C99 and Met-Aβ pools, but their steady-state populations would certainly occur and could be detected in live neuronal cells (see below on human neuronal cells-based AD model, Section 26). These populations, however, would not occur in post-mortem samples since in dying cells, the production of C100 would stop long before the operation of aminopeptidases terminates; consequently, with the influx of C100 and Met-Aβ ceased and aminopeptidases active, the N-terminal Met in question would be removed in its entirety. The presumed ability to distinguish between C100 and *i*Aβ generated independently of AβPP and their counterparts produced by AβPP proteolysis is the key to assaying the validity of the ACH2.0 (see Section 26 below).

### 22.3. Potential Mechanisms of AβPP-Independent Generation of iAβ: Internal Initiation of Translation within the Intact Human AβPP mRNA from the AUG Encoding Met671

Potentially, there are two categories of the mechanisms of AβPP-independent generation of iAβ. One consists of the proposal of Breimer and Danny [140] that in AD, C99 and Aβ could be produced independently of AβPP by translation of the intact human AβPP mRNA initiating within its coding region with Met671. Two research groups attempted to test this proposal. The rationale in both studies was that a manipulation of AβPP DNA (and, consequently, AβPP mRNA) upstream from the ATG encoding Met671 should be inconsequential for translation initiating at this site, and would not interfere with it. In one study, various frame-shift mutations, introduced at the upstream positions, were utilized [150]. Another group inserted a translational stop codon upstream of the ATG in question [151]. In both cases the reasoning was simple: if the internal initiation of translation from the AUG encoding Met671 of AβPP does occur, C99 (and Aβ) would be produced from mutation-carrying AβPP mRNAs; if it does not, neither C99 nor Aβ would be generated. In both cases, C99 and Aβ were not detected, and the postulated phenomenon was declared as “ruled out” [150,151].

But did the above referenced studies really rule out this possibility? The answer is a resolute “NO”. The experimental approaches that both studies have employed to assess the validity of the proposed mechanism resulted in apparently typical cases of comparing the proverbial apples and oranges. The proposal by Breimer and Danny [140] postulated the AβPP-independent production of C99 and Aβ via internal initiation of translation in AD-affected neurons in a *disease-inducible* manner. However, in both studies [150,151] referred to above, the determination ruling out this phenomenon was made in non-neuronal cells and certainly not under the AD conditions. It is, therefore, patently inapplicable to the processes taking place in AD. Consequently, the proposal by Breimer and Danny [140] remains potentially valid and should be re-assessed, along with other possible mechanisms discussed below in the present and following sections, in the suitable AD model (see Section 26 on this subject).

### 22.4. Potential Mechanisms of AβPP-Independent Production of iAβ: Utilization of 5′-Truncated AβPP mRNA Where the AUG Encoding Met671 Is the First Translation Initiation Codon

#### 22.4.1. Internal Initiation of Transcription Upstream from the ATG Encoding Met671 of AβPP

The other category of potential mechanisms of AβPP-independent *i*Aβ generation includes the processes utilizing 5′-truncated AβPP mRNA. It is not difficult to envision that if AβPP mRNA were 5′-truncated in such a way that the first functional in-frame translation initiation codon would be the AUG encoding Met671, a conventional (rather than internally initiated) translation would result in the C100 fragment of AβPP as its primary product. One mechanism capable of generating such truncated mRNA is the internal initiation of transcription. This should take place well within the AβPP coding region but upstream of the AβPP gene segment encoding C99, in such a position that in the resulting mRNA the AUG encoding Met671 of AβPP would be the first functional translation initiation codon. The occurrence of such process would necessitate expression of a suitable transcription factor, or a cofactor, and could be induced upon the elicitation of the integrated stress response.

#### 22.4.2. Targeted Site-Specific Cleavage of AβPP mRNA Upstream from Its C99-Encoding Segment

Another mechanism possibly responsible for the production of suitably 5′-truncated AβPP mRNA is the targeted site-specific cleavage of the intact AβPP mRNA at an appropriate position within its coding region, i.e., upstream from the C99-encoding segment of mRNA. The activation of such a mechanism would require the ISR-enabled expression of a suitable nuclease activity. mRNA produced by the above mentioned two mechanisms would be similar in that they would be encoding the same primary product, namely the C100 fragment of AβPP. On the other hand, they would be distinguishably different: whereas the mRNA product of the internal initiation of transcription would terminate with the cap structure at its 5′ end, the cleavage-resulting mRNA would be cap-less. 

#### 22.4.3. Potential Generation of C100-Encoding mRNA by the Asymmetric Amplification of Human AβPP mRNA

The third and, arguably, most plausible potential mechanism underlying AβPP-independent generation of *i*Aβ in AD is asymmetric RNA-dependent AβPP mRNA amplification. This mechanism is of significant interest because it also offers mechanistic explanation as to why AD is species-specific and possibly human-specific and why it certainly cannot occur in mice and mouse models, even upon an acute exogenous overexpression of human Aβ. Importantly, human AβPP mRNA appears to be its eligible template. If indeed operational in AD, this mechanism would, as detailed below, produce mRNA where the AUG encoding Met671 of AβPP is the first translation initiation codon; it is briefly discussed in the following section.

## 23. RNA-Dependent Amplification of Mammalian mRNA: Human AβPP mRNA Is Uniquely Eligible for the Process That Would Generate mRNA Encoding the C100 Fragment of AβPP

### 23.1. The Chimeric Pathway of Mammalian RNA-Dependent mRNA Amplification

RNA-dependent amplification of mammalian mRNA can occur in two consecutive stages, a “chimeric” pathway that potentially could be followed by a PCR-like mRNA amplification (for detailed discussion see [104,105,106,107,152,153,154,155,156,157]). Only the former is relevant to the subject of the present study. The mRNA amplification pathway of interest is “chimeric” because the resulting product of amplification contains covalently attached sense and antisense RNA segments. The amplified mRNA may be identical to its gene-transcribed progenitor in that it retains the intact protein coding capacity. On the other hand, it is of great potential relevance for AD because the amplification process can also produce mRNA molecules with 5′-truncated coding regions. In fact, the amplification of human AβPP mRNA resulting in 5′-truncated mRNA encoding the C100 fragment of AβPP is indeed plausible and is discussed below.

**Figure 16:**
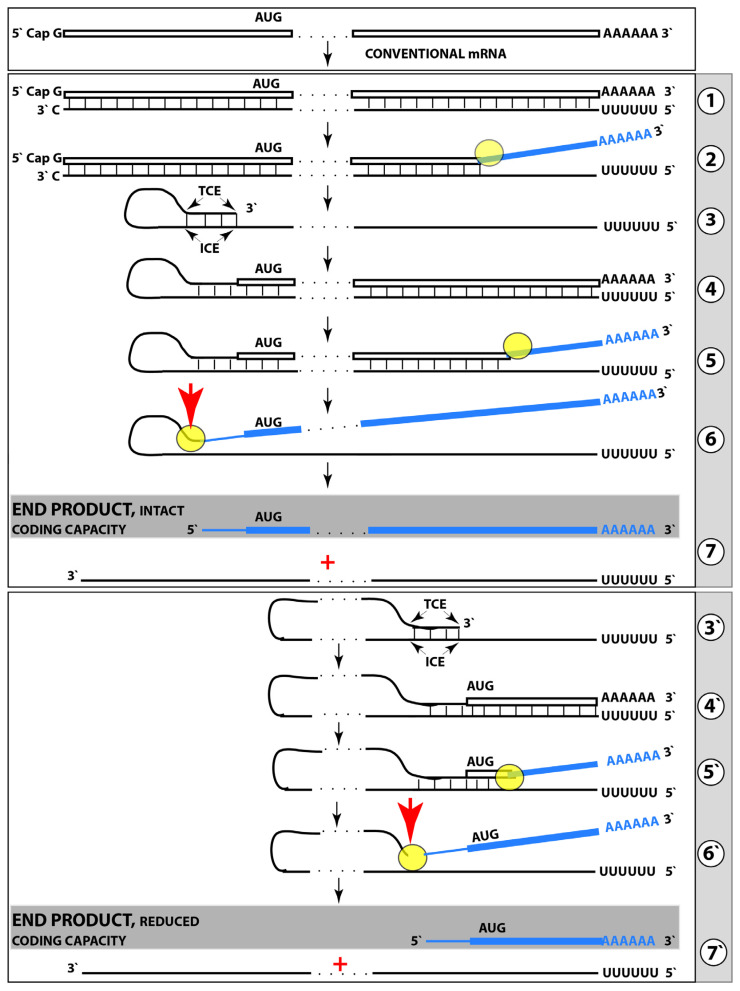
Principal stages of mammalian RNA-dependent mRNA amplification. *Boxed line*: Sense RNA. *Single line*: Antisense RNA. “*AUG*”: Codon for translation-initiating methionine. “*TCE*”: 3′-terminal complementary element of the antisense RNA; “*ICE*”: Internal complementary element of the antisense RNA. *Filled yellow circle*: Helicase/nucleotide-modifying activity complex. *Blue lines* (single and boxed): RNA strands following their separation by a helicase/modifying activity. *Red arrows*: Site of cleavage of the chimeric intermediate. ***Top*** *panel*: The progenitor of the mRNA amplification pathway: conventional, genome-transcribed mRNA. ***Middle*** *panel*: Projected stages of the chimeric pathway of mammalian mRNA amplification. The internal complementary element (ICE) is situated within a portion of antisense RNA corresponding to the 5′UTR of conventional mRNA progenitor; consequently, the chimeric RNA end product contains the entire coding region of conventional mRNA. Stage **1**: RdRp-mediated transcription of the antisense RNA from the gene-transcribed sense RNA progenitor. Stage **2**: Strand separation; helicase activity mounts 3′ poly(A) of the sense RNA and moves along it. Stage **3**: TCE/ICE-facilitated folding of antisense RNA into self-priming conformation. Stage **4**: 3’ terminus of the antisense RNA is extended into the sense RNA. Stage **5**: Double-stranded portion of the hairpin structure is separated into sense and antisense RNA by helicase activity. Stage **6**: When helicase reaches single-stranded portion of hairpin structure, it (or associated activity) cleaves the chimeric intermediate. Stage **7**: 3′-trucated antisense RNA and chimeric RNA end products of the chimeric mRNA amplification pathway. ***Bottom*** *panel*: The ICE element is situated within a segment of antisense RNA corresponding to the coding region of conventional mRNA. Consequently, the amplified chimeric RNA end-product contains a 5′-truncated coding region of conventional mRNA. The translational outcome is decided by the location of the 5′-most translation initiation codon; if it is in-frame, translation would yield the C-terminal fragment of conventionally encoded polypeptide. Stages **3**′ through **7**′ correspond to stages **3** through **7**.

The chimeric pathway of mammalian mRNA amplification is illustrated diagrammatically in Figure 16 and can be briefly summarized as follows. The process is initiated by synthesis of the antisense complement of gene-transcribed mRNA progenitor by the RNA-dependent RNA polymerase (RdRp). It results in a double-stranded structure containing both sense and antisense RNA strands. Strands are then separated by a helicase complex that starts at the poly(A) segment of the 3′-terminal poly(A)-containing strand and moves along it. When separated, sense RNA can be reused as amplification template. Antisense RNA, on the other hand, folds into a self-priming configuration and is extended into the sense RNA. Such folding requires the occurrence of two complementary and topologically compatible elements (nucleotide sequences) within the antisense RNA [158]. One element must be 3’-terminal (the terminal complementary element, TCE), the other (the internal complementary element, ICE) can be located potentially anywhere within antisense RNA. 

When the extension of self-primed antisense into sense RNA strand is completed, it results in a hairpin structure. Complementary strands in this structure are separated by a helicase activity invoked above. When helicase complex reaches the single-stranded portion (loop) of the hairpin structure, it cleaves the RNA molecule at the 3′ end of the loop or at a TCE/ISE mismatch. The cleavage produces two end products of the chimeric amplification pathway. One product is 3’-truncated antisense RNA missing either a part of or the entire TCE element. Another product is the chimeric mRNA. It contains 5’-truncated sense RNA and a covalently bound portion of the antisense RNA (in fact, its cleaved-off 3′-terminal segment). Whereas the antisense end product of the chimeric pathway can be utilized as initial template (progenitor) in the PCR-like mRNA amplification pathway (outside the scope of the present discussion; described in detail in [105,106,156]), the chimeric RNA end product of amplification is a functional mRNA and can be translated into a protein [105,106]. The potential protein product of the chimeric mRNA translation are not necessarily identical to that translated from gene-transcribed mRNA progenitor of amplification; two potential typical outcomes are discussed below.

### 23.2. Chimeric RNA End Products May Retain the Intact Protein Coding Content of the Conventional mRNA Progenitor

Translational outcomes of the chimeric pathway of RNA-dependent mRNA amplification include but are not limited to a protein encoded by the gene-transcribed mRNA progenitor. Chimeric RNA end product of amplification may encode only the C-terminal portion of the corresponding conventionally produced protein. It may join the C-terminal portion of conventionally produced protein with a polypeptide encoded elsewhere in the genome, i.e., the amplification process is able to produce a polypeptide encoded non-contiguously in the genome. Alternatively, the chimeric RNA end product of amplification may encode a polypeptide entirely unrelated to that translated from the conventional mRNA progenitor (for detailed discussion of the above possibilities see [156]). Which translational scenario plays out with a given mRNA species depends principally on the position of the ICE element within the antisense RNA, which, in turn, determines the site of initiation of the extension of the antisense into the sense RNA. The middle panel of Figure 16 illustrates the simplest scenario where the internal complementary element is situated within antisense RNA segment corresponding to the 5′ untranslated region of the gene-transcribed progenitor mRNA. In this scenario, the extension of self-primed antisense RNA would produce a portion of the 5′UTR and the entire coding region of mRNA progenitor. Consequently, the chimeric RNA end product of amplification would differ from corresponding conventionally produced mRNA only in its 5′UTR region and, upon translation, would produce protein identical to the conventionally generated polypeptide.

### 23.3. Chimeric RNA End Products May Encode the C-Terminal Fragment of Conventionally Generated Polypeptide

The 3′ terminal complementary element of the antisense RNA is, by definition, invariably 3′-terminal. On the other hand, the internal complementary element can be literally anywhere within the antisense RNA. A possibility, which is of great interest because it is relevant to the subject of AβPP-independent generation of Aβ, is one whereby the ICE element is located in a portion of the antisense RNA corresponding to the coding region of conventional gene-transcribed mRNA progenitor. This particular scenario is illustrated in the bottom panel of Figure 16. In this case, the extension of self-primed antisense RNA would produce only the 3′ portion of conventional mRNA containing only the 3′ portion of its coding region. Accordingly, the chimeric RNA end product of amplification would contain 5′ truncated coding region of conventional mRNA. The translational product generated from such RNA would be defined by the location of the first, 5′-most AUG or another translation initiation-competent codon. If this codon were in-frame, translation of the chimeric RNA product of mRNA amplification would produce the C-terminal fragment of conventional mRNA progenitor-encoded protein. This type of the chimeric mRNA amplification pathway would be asymmetric: Indeed, only the 3′-terminal segment of the coding region would be amplified, and its translation would generate only the C-terminal segment of conventionally produced protein. Asymmetric chimeric mRNA amplification pathway can also result in several additional interesting translational outcomes, which are outside of the scope of the present discussion and are described elsewhere [156].

## 24. Human AβPP mRNA Is an Eligible RdRp Template: Projected Pathway of Asymmetric Amplification Resulting in Chimeric mRNA Encoding the C100 Fragment of AβPP

The asymmetric chimeric pathway of RNA-dependent mRNA amplification described above is potentially capable, if applicable to AβPP mRNA, of generating mRNA encoding C100 fragment independently of AβPP. The asymmetry involved in producing 5’-truncated AβPP mRNA where the AUG codon for methionine 671 is the 5′-most translation initiation codon is, however, extensive. The AUG encoding methionine 671 of AβPP is located more than two thousand nucleotides downstream from the 5′ end of AβPP mRNA. Consequently, the TCE and ICE complementary elements within the antisense RNA (if they occur in the first place) would be as distant from each other. Provided that the TCE and ICE do occur in suitable positions, the question is: would the required antisense RNA folding be feasible, i.e., would the complementary elements be topologically compatible in folded antisense AβPP conformation? The general approach (i.e., not only for AβPP mRNA but for any mRNA species) to assess this is as follows.

### 24.1. Assessment of the Eligibility of an mRNA for RNA-Dependent Amplification: General Approach

In this general approach, the mRNA of interest is reverse transcribed into an antisense cDNA. The mRNA template is removed by RNase H activity (which cleaves RNA in double-stranded RNA/DNA substrate and usually occurs in preparations of reverse transcriptase, RTase, unless removed genetically) concurrently with the reverse transcription progression and mRNA template’s engagement in a double-stranded structure with newly synthesized cDNA (thus forming RNase H substrate). Provided the mRNA of interest is fully transcribed, provided the TCE and ICE elements occur within the antisense strand, and provided they are topologically compatible, the antisense strand would fold into self-priming conformation and would be extended (by RTase, which is capable of utilizing both RNA and DNA templates) into the sense strand. Such extension could be easily detected by nucleotide sequencing, and the junction between the sense and antisense segments would indicate the position of the ICE element and enable the identification of both TCE and ICE.

### 24.2. Human AβPP mRNA Is Eligible for RNA-Dependent Amplification

**Figure 17:**
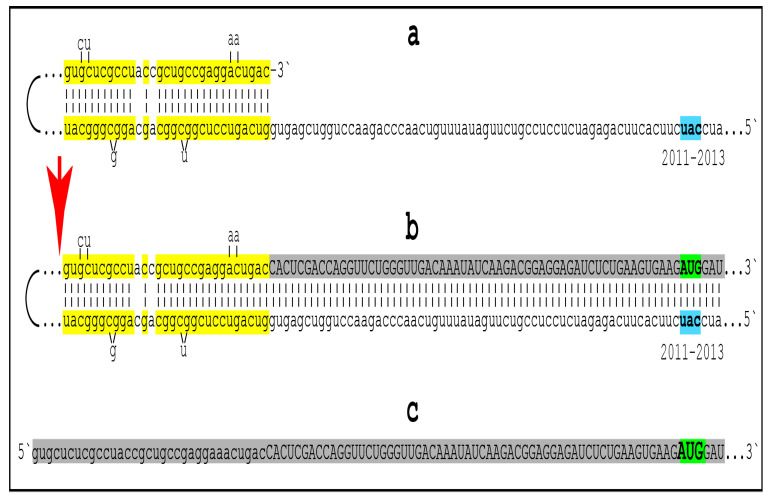
Chimeric pathway of human AβPP mRNA amplification resulting in mRNA encoding the C100 fragment of AβPP: Projected principal stages. *Lowercase letters*: Nucleotide sequence of the antisense RNA. *Uppercase letters*: Nucleotide sequence of the sense RNA. Highlighted *in yellow*: The 3′ terminal (**top**) and the internal (**bottom**) elements of the human antisense AβPP RNA. “*2011*–*2013*”: Nucleotide positions (from the 3′ terminus of the antisense AβPP RNA) of the “uac” (highlighted *in blue*) corresponding to the “AUG” (highlighted *in green*) encoding Met671 in the human AβPP mRNA. *Panel **a***: TCE/ICE-facilitated folding of the human AβPP antisense RNA into self-priming configuration. *Panel **b***: Extension of self-primed AβPP antisense RNA into sense RNA (highlighted *in gray*). *Red arrow*: Cleavage of chimeric RNA intermediate following separation of sense and antisense RNA (not shown). The cleavage is shown at the 5′ end of the TCE element; it may also occur at one of the TCE/ICE mismatches. *Panel* **c**: Chimeric RNA end product of RNA-dependent amplification of human AβPP mRNA (highlighted *in gray*). It consists of antisense portion (the TCE or part thereof) extended into 5′ truncated coding region of human AβPP mRNA. Its first, 5′-most translation initiation codon is the in-frame AUG (highlighted *in green*) that encodes Met671 of human AβPP; when translated, it would produce the C100 fragment of AβPP.

Just such an assessment was actually inadvertently performed for human AβPP mRNA. Soon after human AβPP cDNA was sequenced [141,142,143], but before the genomic sequence upstream of the human AβPP gene was defined, one research group reported the detection and sequencing of much longer human AβPP cDNA, which was significantly extended at its 3′ end [159]. The authors hypothesized that it originated from 5′-extended AβPP mRNA whose transcription was initiated at the alternative transcription initiation site upstream from originally reported 5′ end of the human AβPP gene (based on the AβPP cDNA sequencing, [141,142,143]). Soon after [159] was published, however, the genomic region upstream of human AβPP was sequenced [160], and it became apparent that the observed human AβPP cDNA extension seen in [159] could not have originated by alternative initiation of transcription upstream from AβPP gene. Consequently, following the publication of genomic nucleotide sequence upstream from the human AβPP gene [160], the authors of [159] declared their result an artifact and published a correction to this effect [159]. However, a close analysis of human AβPP cDNA extension obtained in [159] showed that the extended portion is, in fact, a perfect segment of human AβPP sense strand, and that it was derived by the extension of folded and self-primed AβPP cDNA (antisense strand) that occurred about 2000 nucleotides from the 3′ terminus of the AβPP cDNA. The sense/antisense junction within extended AβPP cDNA defined the site of the initiation of extension and enabled the determination of sequences of the TCE and ICE elements [161,162,163]. Moreover, this analysis showed that the first, 5′-most translation initiation codon within the sense segment of the extended AβPP cDNA is, in fact, the ATG encoding Met671 of AβPP [161,162,163]. The projected folding, self-priming and the extension of human antisense RNA AβPP strand, as well as the cleavage generating the mRNA encoding the C100 fragment of human AβPP, are illustrated in Figure 17. Note that stages **a**, **b**, and **c** of asymmetric RNA-dependent AβPP mRNA amplification depicted in Figure 17 correspond to stages **3**′ through **6**′ shown in Figure 16.

As shown in Figure 17, the human antisense AβPP RNA contains the TCE and ICE elements (the TCE becomes such only if it has the complementary and topologically compatible ICE partner; otherwise it is just a 3′ terminal RNA segment). The distance between the TCE and ICE is quite large, about two thousand nucleotides, yet they guide the folding of the antisense RNA into a self-priming conformation. The TCE element acts as a primer and is extended by RdRp into sense RNA strand (transcribing, in fact, a 5′ portion of the antisense RNA template). Sense and antisense RNA strands are then separated by helicase activity (not shown in Figure 2). When helicase reaches the single-stranded portion of the molecule, it, or associated activity, cleaves it. In Figure 2, the cleavage is shown to occur at the 5′ end of the TCE element (i.e., the 3′ end of the single-stranded loop of the hairpin structure), but it could also occur at one of the TCE/ICE mismatches. This cleavage produces the chimeric end product of asymmetric RdRp-mediated, RNA-dependent amplification of human AβPP mRNA. Its sense RNA segment consists of severely 5′ truncated coding region of AβPP mRNA continued into its 3′ untranslated region. In terms of the translational outcome, the most important feature of this amplified RNA is the presence of in-frame translation initiation codon. It occurs 58 nucleotides downstream from the sense/antisense junction, and *it is the AUG codon for methionine 671 of AβPP.* Thus, *the chimeric RNA end product of the asymmetric amplification of human AβPP mRNA would produce, upon its translation, the C100 fragment of AβPP*. Importantly, this mode of production, eventually resulting in Aβ, would be completely independent of AβPP. 

## 25. The Unique Eligibility of Human AβPP mRNA for Asymmetric RNA-Dependent Amplification Provides Explanation for Species-Specificity, Possibly Human-Specificity, of AD

The potential utilization of the asymmetric AβPP mRNA amplification in the second stage of AD provides an explanation as to why AD appears to be human-specific (or, at least, species-specific; indeed, it does not occur even in long-living mammals, such as elephants). As was mentioned above, at least two requirements have to be met for RNA-dependent mRNA amplification to occur: (a) the occurrence of two complementary elements, TCE and ICE, on the antisense strand and, if this is satisfied, (b) topographical compatibility of the TCE and ICE, i.e., the sufficient spatial proximity in the folded antisense configuration allowing their interaction and formation of a self-priming structure. It appears that in non-human mammals even the first requirement is not met [156]. It certainly is not met in mice, where AβPP antisense RNA segments corresponding to the TCE and ICE elements of human AβPP antisense RNA show little, if any, complementarity [161,162,163]. Moreover, this requirement is not met in human AβPP mRNA exogenously overexpressed in mice because its 5’-terminal region is substantially modified during the construction of expression vectors or of transgenes, and therefore it loses its RdRp eligibility. This can be the reason why transgenic AD models do not and apparently cannot exhibit the full spectrum of AD pathology. Importantly, however, as argued above, the absence of the operative AβPP-independent *i*Aβ generation pathway does not preclude in any way the occurrence of AACD.

## 26. Testing the Validity of the ACH2.0 and the Potential of the BACE1/BACE2 Activation-Mediated *i*Aβ Depletion Therapy

### 26.1. Human-Neuronal-Cell-Based AD Model 

#### 26.1.1. Rationale

For two reasons, the best conceivable AD model is, arguably, that based on human neuronal cells. The first reason is that such model utilizes cells originating from the species known to be affected by AD. The second reason is that, as discussed above, AD appears to be human-specific or, at least, species-specific [152,153,154,155,156,157], i.e., human cells seem to possess unique feature(s), possibly the ability to produce *i*Aβ in the AβPP-independent mode [156] or enact some other mechanism(s) enabling the second AD stage, that are, because of the structure of their AβPP mRNA or for other reasons, unavailable in non-human mammalian species [161,162,163]. Since human neurons are intrinsically capable of the molecular processes underlying the disease and, more specifically, are capable of enabling the stage two of AD, the design principles to generate the AD model are relatively straightforward; they aim to trigger the second stage of AD and to activate the AβPP-independent *i*Aβ generation pathway and, consequently, the AD Engine. Once this occurs, the progression of cellular AD pathology, driven by the AβPP-independent *i*Aβ production pathway, would become self-sustaining and irreversible (unless intervened in therapeutically). Moreover, as described below, the ability of a human neuronal cells-based AD model system to support the formation of hyperphosphorylated tau tangles, the major cellular AD hallmark, has been proven experimentally [61].

#### 26.1.2. Cultured Human Neuronal Cells Are Capable of Displaying Full Spectrum of Cellular AD Pathology 

Another decisive advantage of a human neuronal cells-based AD model is its apparent capacity to present the complete spectrum of cellular AD pathology, including the formation of neurofibrillary tangles (NFTs). Exogenous overexpression of Aβ in human neuronal cells cultured in matrigel was indeed shown to trigger the formation of this principal AD hallmark [61]. In the study under discussion, the authors adopted the ACH-based interpretation of the observed phenomenon and ascribed it to the effects of overexpressed extracellular Aβ produced in the AβPP proteolytic pathway. The ACH2.0-based interpretation of the same results offers a completely different picture. It suggests that in their experiments authors inadvertently activated the AβPP-independent *i*Aβ generation pathway and, consequently, ignited the AD Engine. In this study [61], investigators utilized polycistronic lentiviral construct to acutely overexpress human AβPP cDNA carrying the London FAD mutation V717I as well as the Swedish FAD mutation K670N/M671L. From the same construct, they also overexpressed PSEN1 carrying the FAD mutation ∆E9. Following the introduction of lentiviral construct, human neural progenitor cells were cultured and differentiated in matrigel [61]. To understand how the mutations employed in this study could facilitate the activation of the AβPP-independent *i*Aβ generation pathway, it is important to review their function. The Swedish FAD mutation furthers, as discussed above, the gamma-cleavage of C99 on intracellular membranes and, consequently, facilitates the retention of *i*Aβ produced by the AβPP proteolysis [59]. The London FAD mutation significantly increases production of Aβ_42_ isoform and so does the PSEN1 mutation utilized in this study. Both mutations substantially accelerate the accumulation of AβPP-derived *i*Aβ. This occurs for three reasons. First, secreted Aβ does not diffuse in the matrigel. Second, secreted Aβ_42_ is internalized preferentially and with augmented efficiency (versus other Aβ species) [35]. Third, as discussed above, intraneuronal Aβ_42_ lowers (in comparison with other Aβ isoforms) the **T1** threshold and thus facilitates the activation of the AβPP-independent *i*Aβ production pathway. Therefore, *i*Aβ_42_ rapidly reaches the **T1** threshold and triggers the endogenous AβPP-independent generation of *i*Aβ. The drastically increased levels of *i*Aβ drive cellular AD pathology, apparently including the formation of NFTs. The high *i*Aβ levels indeed were shown to inhibit the ubiquitin–proteasome system, facilitate the accumulation and phosphorylation of tau protein and promote the formation of NFTs [134,135,136,137]. Whereas the human-neuronal-cell-based AD model employed in [61] can be utilized for testing (see below) the validity of the ACH2.0 (in fact, it is currently the only existing AD model suitable for this purpose), the following section describes simpler and more streamlined approaches to generate a human-neuronal-cell-based model capable of accomplishing this objective.

### 26.2. Human-Neuronal-Cell-Based AD Model: Principles of Design

Within the framework of the ACH2.0, the most “physiological” (i.e., imitating the processes that occur in the disease) approach to ignite the AD Engine, i.e., to activate the endogenous AβPP-independent *i*Aβ production and to initiate the second stage of AD, is to rapidly accumulate *i*Aβ to the **T1** threshold. Considering, as described above, that in AD-predisposed individuals, the **T1** threshold could be significantly lower than in general population, it may be useful to utilize neurons differentiated from iPSCs of AD patients who developed AD in their middle to late sixties. Rapid accumulation of *i*Aβ can be accomplished by transiently and exogenously expressing Aβ_42_ (because it further lowers the **T1** threshold) from DNA constructs or from transfected mRNA, or by importing it directly by electroporation or by other suitable technique. The expression constructs should encode Aβ_42_ rather than AβPP or C99; the resulting peptide would be *i*Aβ (i.e., it would not be secreted). If, in an exogenous *i*Aβ expression construct, translation would initiate from the AUG contiguously preceding the Aβ segment of the expression vector, the resulting primary product would be Met-Aβ. However, the principal detection assays in the validation procedure would be for the occurrence of endogenously produced C100 and Met-Aβ, and the presence of exogenously generated Met-Aβ would interfere with the detection of its endogenous counterpart. 

A solution to the above problem is to arrange for a co-translational removal of the translation-initiating methionine by MAP1 or MAP2. This can be accomplished by inserting, immediately following the initiating methionine, one of the seven residues (listed above) that are compatible with the operation of MAP1/MAP2 [147]. The best choice appears to be Val because it would confer the longest half-life, according to the N-end rule [146]. However, when the N-terminal methionine is followed by valine, its removal by MAP1/MAP2 depends on the next downstream residue. If it is aspartate, as is the case in Aβ, the initiating methionine cannot be cleaved-off by MAP1/MAP2 [147], and the resulting primary translation product would be Met-Val-Aβ. The next best choice, in accordance with the N-end rule, is Gly. In this case, the translation initiating methionine will be removed co-translationally by MAP1 or MAP2 [147] and the primary translation product would be Gly-Aβ, which can be readily distinguished from the Met-Aβ presumably generated endogenously in the AβPP-independent manner. In a complementary approach, if exogenously expressed *i*Aβ contains one of FAD mutations, say the Swedish mutation, not present in the endogenously produced Aβ, this feature would assist in distinguishing between the two.

On the other hand, however, the identification of the endogenously produced C100 fragment of AβPP would be fully satisfactory, at least initially, for the purpose of the validation of the ACH2.0. If such a procedure is adopted, Met-Aβ can be unreservedly expressed exogenously in human neuronal cells with the aim to activate the endogenous AβPP-independent C100 and *i*Aβ production. Such an approach would not interfere with the C100-based detection procedure. 

Alternative, albeit less “physiological”, approaches, which bypass the *i*Aβ accumulation stage, include the induction of mitochondrial dysfunction resulting in the HRI activation or stressor-specific activation of one of the other eIF2α kinases, all leading to the elicitation of the ISR and, provided that the ISR alone is sufficient to activate the AβPP-independent *i*Aβ generation pathway and, consequently, the AD Engine, resulting in the commencement of the second AD stage.

### 26.3. Testing for the Principal Hallmark of the ACH2.0: AβPP-Independent Generation of iAβ 

As was discussed above, the AβPP-independent generation of *i*Aβ may conceivably occur via four distinct mechanisms: (a) internal initiation of transcription of the AβPP gene, (b) site-specific cleavage of AβPP mRNA, (c) asymmetric RNA-dependent amplification of AβPP mRNA, and (d) internal initiation of translation within AβPP mRNA. All these mechanisms have one common principal feature: in each, the AβPP-independent generation of *i*Aβ occurs via translation initiated at the AUG codon encoding Met671 of AβPP. Accordingly, the primary translation product in every mechanism is the C100 fragment of AβPP, i.e., N-terminal methionine-containing C99. This is because, as described above, the N-terminal methionine cannot be removed co-translationally by MAP1 or MAP2, and its removal is effected only post-translationally by an aminopeptidase(s) with broad specificity. Consequently, in neuronal cells with the activated AβPP-independent *i*Aβ production pathways, steady-state pools of N-terminal-Met-containing C99 and Met-Aβ should be present; either of these pools would constitute the unique identifier of the activity of the pathway. Therefore, the detection of either or both would provide an unambiguous proof of the operation of the AβPP-independent *i*Aβ generation pathway.

If such proof were obtained, a question could be addressed which of the four mechanisms described above enables the operation of the AβPP-independent *i*Aβ production pathway. Three of the four could be identified by the analysis of mRNA encoding the C100 fragment (the only requirement for such mRNA is that the first functional translation initiation codon is the AUG encoding Met671 in AβPP mRNA). If the mRNA in question were capped at its 5′ terminus, it would suggest its origin via the internal initiation of transcription within the AβPP gene. If the mRNA in question were an uncapped suitable fragment of AβPP mRNA, its origin is likely a site-specific cleavage of AβPP mRNA. The observation that RNA of interest is chimeric, i.e., contains an antisense segment at its 5′ portion, with the predictable sequence and position of the sense/antisense junction, identical to or resembling that shown in Figure 17 (cleavage of the pinhead chimeric RNA intermediate could occur at TCE/ICE mismatches, and the remaining antisense self-priming structure could be stable enough to initiate a new extension cycle, thus generating slightly different sense/antisense junction sequences, a phenomenon termed the “Chimeric Junction Shift”; see ref. [156]), would indicate the occurrence of the asymmetric RNA-dependent amplification of AβPP mRNA. If none of the above were detected, the internal initiation of translation of AβPP mRNA would be indicated. The occurrence of the latter could be tested in ways conceptually similar to those employed in the original testing of this notion (when it was arbitrarily “ruled out” [150,151]) but in the proper human neuronal cells-based model system. This can be accomplished by editing-in the endogenous AβPP gene either a frame-changing mutation or a stop codon mutation upstream from the AUG encoding Met671 of AβPP. If the internal initiation of AβPP mRNA translation does occur, these mutations should not interfere with it; if it does not, no Aβ would be produced endogenously. The criteria of the endogenous origin could be the presence of N-terminal Met on C99 or *i*Aβ. Alternatively, as described above, an arbitrary FAD mutation could be introduced within the exogenously produced *i*Aβ to distinguish it from its endogenously derived counterparts. A possible involvement of the internal ribosome entry site (IRES) in the internal initiation of translation of AβPP mRNA can be also studied using standard methods of analysis [164,165,166,167,168,169]. If, on the other hand, no pools of either C100 or Met-Aβ are detected in the human-neuronal-cell-based AD model despite the occurrence of NFTs, the conclusion, apparently unlikely but nevertheless possible, would be that the agent driving the second AD stage is not *i*Aβ, a scenario considered in detail in [2].

### 26.4. Testing the Therapeutic Potential of the BACE1/BACE2 Activation-Mediated iAβ Depletion Therapy

To assess the therapeutic potential of *i*Aβ depletion, BACE1 and/or BACE2 can be exogenously overexpressed from either a constitutive or an inducible promoter (the latter to allow evaluation at the different mechanistic stages). Assaying options for the assessment of the effects and consequences of BACE overexpression would depend on the determination of the pathway underlying operation of the AD Engine, as described in the preceding section. If this pathway is the AβPP-independent *i*Aβ production, the assaying could be extensive. It would include monitoring the levels of the intact *i*Aβ (expected to be reduced by Aβ-cleaving activities of BACE1 and BACE2) and testing the activity of the AβPP-independent *i*Aβ production pathway by examining the occurrence of C100 (Met-C99) and of Met-Aβ. If the *i*Aβ depletion were successful, the AD Engine’s operation would cease. Consequently, C100 influx would stop and it, as well as Met-Aβ, would dissipate (this is why these species cannot be present in postmortem samples: in dying neurons, the production of Met-C99 and Met-*i*Aβ would cease while aminopeptidases are still operational; consequently no N-terminal methionine would remain); thus, the occurrence of C100 and/or Met-Aβ, or lack thereof, would report on the activity of the AβPP-independent pathway of its production. If, on the other hand, the second AD stage is driven not by *i*Aβ but by another, yet unidentified, agent, assaying options would be limited to determining levels of the intact *i*Aβ and to monitoring hyperphosphorylation of tau protein and the formation of NFTs; in any case, the potential therapeutic effects of the *i*Aβ depletion treatment could be quantified. The ability to regulate the activity of the AβPP-independent *i*Aβ generation pathway and, even more importantly, to control the formation of NFTs via BACE-mediated *i*Aβ depletion would constitute a proof of principle for the utilization of BACE activators (or other *i*Aβ-depleting agents) as potential AD drugs and would justify a major effort to develop such agents. 

As for assessing effects of the proposed therapy in AACD, this can be and indeed was performed numerous times with successful outcomes (using drugs suppressing the accumulation of AβPP-derived *i*Aβ) in currently available transgenic mouse models where, as argued above, the second AD stage does not occur. This is because, whereas these models do not develop AD, the neuronal damage and the cognitive dysfunction symptoms that they exhibit are, at least in part and possibly in full, caused by *i*Aβ (produced by AβPP proteolysis and both internalized and retained intraneuronally) and are equivalent to the pathology displayed in AACD. Moreover, whenever the effective BACE1- and/or BACE2-activating (or any suitable *i*Aβ-depleting) candidate drugs are available, their effect on AACD could be tested in mouse models exogenously overexpressing Aβ. Because of the lack of definitive cellular AACD-specific markers, it is currently not feasible to test directly the therapeutic potential of the *i*Aβ depletion in AACD in humans or in the human-neuronal-cell AD model. However, because the age of onset of AACD is apparently greater than that of typical SAD [100,101], any human clinical trials testing the effect of the *i*Aβ depletion in prevention of SAD would, by default, also test the effect of the *i*Aβ depletion therapy in prevention of AACD. 

## 27. Conclusions

The recently posited amyloid cascade hypothesis 2.0 envisions AD as a two-stage disorder. The first stage is a life-long accumulation of intraneuronal AβPP-derived *i*Aβ. This occurs via cellular uptake of secreted Aβ and through retention of a fraction of Aβ produced by AβPP proteolysis. When AβPP-derived *i*Aβ reaches critical **T1** threshold, it activates a self-sustaining production of an agent that drives the second AD stage, i.e., a cascade including tau pathology and culminating in neuronal loss. It is highly probable that this agent is *i*Aβ generated independently of AβPP. The detection of a single AβPP mutation affecting Aβ and causing familial AD [7] was deemed sufficient to formulate the ACH. In the thirty years that followed, many additional FAD mutations were detected. All of them, without a single exception, affect, in one way or another, Aβ. This is consistent with the notion that *i*Aβ, differentially derived, runs the entire course of AD, a concept supported by the occurrence of several cellular mechanisms capable of producing *i*Aβ independently of AβPP. The rate of accumulation of AβPP-derived *i*Aβ and the extent of the **T1** threshold determine the timing of the commencement of the second AD stage and, given the limited lifespan, define the susceptibility to AD. 

The present study analyzes the dynamics of AβPP-derived *i*Aβ and the role of the extent of the **T1** threshold in the disease. It formulates principles of dynamics of AD and of aging-associated cognitive dysfunction and defines AACD as an extended segment of the first AD stage, thus incorporating it into the ACH2.0. It explains why only a fraction of the population develops sporadic AD and AACD and why both pathologies are age-dependent. It provides mechanistic interpretations for all principal aspects of AD and AACD, including the protective effect of Icelandic AβPP mutation, the early onset of FAD and the temporally sequential manifestation of AD in defined regions of the affected brain, and explains mechanisms underlying the observed effect of lecanemab and donanemab at the early symptomatic stage of AD. It offers a therapeutic strategy that emulates and substantially improves upon the mode of operation of the Icelandic AβPP mutation, which confers on its carriers protection from both AD and AACD. It also posits that a single, once-in-a-lifetime-only, administration of *i*Aβ depletion treatment via transient activation of BACE1 and/or BACE2, exploiting their Aβ-cleaving activities, or by any other suitable means, would not only prevent AD and AACD but would also be effective at the symptomatic stages of both disorders. Validation of the ACH2.0 and of the effectiveness of the proposed ACH2.0-based *i*Aβ depletion therapy would justify a major effort to develop operative BACE1 and BACE2 activators, or other suitable *i*Aβ-depleting agents, as potential preventive and curative AD and AACD drugs. 

The postulated greatly accelerated production of the C99 fragment in the AβPP-independent pathway in symptomatic AD is not only responsible for the augmented generation of *i*Aβ but is also consistent with a growing body of evidence indicating the deleterious role of AβPP intracellular domain (AICD) in the disease. AICD is generated by the epsilon cleavage of C99 downstream and, apparently, independently from the gamma cleavage [170]. AICD interacts with numerous regulatory proteins and signaling pathways [171,172,173,174,175,176,177,178,179,180,181]. It is involved in transcriptional regulation, apoptosis, and cytoskeletal dynamics [182,183]. It affects expression of neprilysin [184] and contributes to tau phosphorylation [171,182]. It alters neuron firing, modifies hippocampus oscillations, and impairs spatial memory encoding [185]. Within the framework of the ACH2.0, the levels of AICD increase significantly in the second, symptomatic AD stage due to the operation of the AβPP-independent *i*Aβ production pathway. Indeed, the primary translation product of this pathway is C100, which gives rise not only to *i*Aβ but also to AICD. The levels of the latter increase in parallel with those of the former, hence its deleterious effect. In this sense the increase in AICD levels in symptomatic AD is a direct result of the activity of the AβPP-independent *i*Aβ (and AICD) production pathway and thus the integral part of the ACH2.0. Importantly, the therapeutic strategies proposed in the present study apply to AICD as well as to *i*Aβ. When implemented preventively, they would preclude the activation of the AβPP-independent *i*Aβ *and AICD* generation pathway. Consequently, there would be no increase in the levels of either *i*Aβ or AICD. When employed in symptomatic AD, the targeted *i*Aβ degradation and its consequent depletion would stop the operation of the AβPP-independent *i*Aβ generation pathway; the production of AICD in this pathway would also cease.

Whereas AD and AACD are associated with aging, they are not the typical aging-caused diseases. They are linked with aging due to the slow rate of the accumulation of *i*Aβ produced in the AβPP proteolytic pathway, a process which occurs presumably linearly from the childhood and whereby *i*Aβ levels start reaching the thresholds that trigger sporadic AD or AACD only in the seventh or eighth decade of life. The occurrence of the disease is the function of a sufficient *i*Aβ accumulation, not of the “aging” per se. Indeed, in the past, with shorter average lifespan and the “aging” occurring earlier, a smaller fraction of the population was reaching their sixties and seventies, and, accordingly, the prevalence of AD was lower than currently. Likewise, prolonging the human lifespan would not shift the occurrence of both conditions to the more advanced “new old age”; instead, it would commence at about the same age (sixties and seventies) as now, constituting a “new middle age”. At the current average lifespan of nearly eighty years, *i*Aβ levels reach the AD-triggering threshold in the advanced age in only about 10% of the population; this fraction is, however, the proverbial “tip of the iceberg”. Given a sufficiently long lifetime, the occurrence of both AD and AACD would *inevitably* become nearly universal since, in the absence of preventive treatment, the disease-triggering *i*Aβ thresholds would be eventually crossed in every (or nearly every) individual, i.e., the entire “iceberg” would be eventually affected. Indeed, with the presumed linear rate of accumulation of AβPP-derived *i*Aβ, the anticipated, potentially highly substantial, increase in longevity in the near future would be accompanied by a corresponding increase in the prevalence, possibly approaching the entirety of the population, of AD and AACD. The proposed once-in-a-lifetime (or twice in a 150-year-long lifetime) preventive *i*Aβ depletion treatment provides an attractive solution.

## Data Availability

Not applicable.
